# Digital Twin Cognition: AI-Biomarker Integration in Biomimetic Neuropsychology

**DOI:** 10.3390/biomimetics10100640

**Published:** 2025-09-23

**Authors:** Evgenia Gkintoni, Constantinos Halkiopoulos

**Affiliations:** 1 Department of Medicine, University of Patras, 26504 Patras, Greece; evigintoni@upatras.gr; 2 University General Hospital of Patras, 26504 Patras, Greece; 3 Department of Management Science and Technology, University of Patras, 26334 Patras, Greece

**Keywords:** digital twin cognition, artificial intelligence, digital biomarkers, biomimetic neuropsychology, personalized cognitive assessment, multimodal integration, neurodegenerative diseases, neuropsychology

## Abstract

(1) Background: The convergence of digital twin technology, artificial intelligence, and multimodal biomarkers heralds a transformative era in neuropsychological assessment and intervention. Digital twin cognition represents an emerging paradigm that creates dynamic, personalized virtual models of individual cognitive systems, enabling continuous monitoring, predictive modeling, and precision interventions. This systematic review comprehensively examines the integration of AI-driven biomarkers within biomimetic neuropsychological frameworks to advance personalized cognitive health. (2) Methods: Following PRISMA 2020 guidelines, we conducted a systematic search across six major databases spanning medical, neuroscience, and computer science disciplines for literature published between 2014 and 2024. The review synthesized evidence addressing five research questions examining framework integration, predictive accuracy, clinical translation, algorithm effectiveness, and neuropsychological validity. (3) Results: Analysis revealed that multimodal integration approaches combining neuroimaging, physiological, behavioral, and digital phenotyping data substantially outperformed single-modality assessments. Deep learning architectures demonstrated superior pattern recognition capabilities, while traditional machine learning maintained advantages in interpretability and clinical implementation. Successful frameworks, particularly for neurodegenerative diseases and multiple sclerosis, achieved earlier detection, improved treatment personalization, and enhanced patient outcomes. However, significant challenges persist in algorithm interpretability, population generalizability, and the integration of healthcare systems. Critical analysis reveals that high-accuracy claims (85–95%) predominantly derive from small, homogeneous cohorts with limited external validation. Real-world performance in diverse clinical settings likely ranges 10–15% lower, emphasizing the need for large-scale, multi-site validation studies before clinical deployment. (4) Conclusions: Digital twin cognition establishes a new frontier in personalized neuropsychology, offering unprecedented opportunities for early detection, continuous monitoring, and adaptive interventions while requiring continued advancement in standardization, validation, and ethical frameworks.

## 1. Introduction

The convergence of artificial intelligence (AI), digital biomarkers, and cognitive modeling has ushered in a transformative era for neuropsychological assessment and intervention. Digital twin technology, originally developed for industrial applications, now presents unprecedented opportunities for creating personalized cognitive health models that can predict, monitor, and optimize individual neuropsychological functioning. This systematic review examines the current landscape of digital twin cognition, focusing on the integration of AI-driven biomarkers within biomimetic neuropsychological frameworks [1,2,3,4,5].

The concept of digital twins in healthcare represents a paradigm shift from population-based approaches to truly personalized medicine. Digital twins—virtual models with dynamic links to physical entities—have emerged as transformative tools in healthcare, facilitating deeper understanding of disease progression and treatment customization. By creating dynamic, data-driven virtual representations of individual cognitive systems, digital twins enable continuous monitoring, predictive modeling, and personalized intervention strategies. Recent advances have seen the integration of large language models (LLMs) and artificial intelligence technologies with digital biomarkers, offering sophisticated analytical capabilities that can process complex, multimodal data streams to generate actionable insights about cognitive health. This approach has shown particular promise in neurodegenerative diseases, with applications ranging from Alzheimer’s disease progression modeling to multiple sclerosis management [6,7,8].

Recent advances in machine learning, particularly deep learning architectures, have dramatically enhanced our ability to detect subtle patterns in neuropsychological data that may indicate early cognitive decline or predict future trajectories. These AI-driven approaches, when combined with digital biomarkers derived from various sources—including neuroimaging, wearable sensors, and behavioral assessments—create comprehensive cognitive profiles that evolve in real-time. The biomimetic aspect of these systems ensures that digital models accurately reflect the complex, dynamic nature of human cognition, incorporating principles from neuroscience, psychology, and computational modeling [9,10,11,12,13,14,15,16,17].

Digital twin cognition establishes a prospective integration framework for multilevel explainable models and digital twins through the association of behavioral descriptions and explanatory artificial intelligence (AI). This integration essentially extends the functionality of digital twins to generate cognitive models of the underlying processes, which form the basis of the observed surface behavior [18,19,20]. Thus, digital twins not only replicate the physical dynamics but also incorporate biomimetic and AI-derived neuropsychological models for comprehensive cognitive representation [21,22,23,24].

The global burden of neurodegenerative diseases and mental health disorders underscores the urgency of developing reliable digital twin systems for cognitive health. Alzheimer’s disease alone affects millions globally, with prevalence expected to triple by 2050. Early detection of conditions such as mild cognitive impairment (MCI) and Alzheimer’s disease remains a critical challenge in clinical practice, complicated by the broad range of disease severity and heterogeneous presentation. Traditional diagnostic methods often fail to capture the subtle, early-stage changes that precede clinical symptoms, resulting in delayed interventions when therapeutic options may be less effective. Digital twin technology has emerged as a promising solution, with recent studies demonstrating its ability to model disease progression across the spectrum from MCI to advanced Alzheimer’s disease. These virtual representations can identify at-risk individuals years before symptom onset, enabling preventive strategies and early interventions that could significantly alter disease trajectories [25,26,27].

Furthermore, the COVID-19 pandemic has accelerated the adoption of digital health technologies and highlighted the need for remote, scalable cognitive assessment tools. Digital twins can facilitate continuous monitoring without requiring frequent clinical visits, making cognitive health assessment more accessible and reducing barriers to care. However, realizing these benefits equitably requires addressing potential algorithmic biases that could disadvantage underrepresented populations, necessitating inclusive data collection and validation across diverse demographic groups to prevent perpetuation of existing healthcare disparities. This is particularly important for elderly populations and individuals in underserved areas who may face challenges accessing specialized neuropsychological services [28,29,30,31,32,33,34].

The integration of multiple data modalities represents another significant advantage of digital twin approaches. By combining neuroimaging data, genetic information, lifestyle factors, environmental exposures, and real-time behavioral metrics, these systems can create holistic models of cognitive function that account for the complex interplay of factors influencing brain health. This multimodal integration is essential for understanding the heterogeneous nature of cognitive disorders and developing personalized intervention strategies [35,36,37,38,39,40].

However, the development and implementation of digital twin cognition systems face several challenges. These include ensuring data privacy and security, addressing algorithmic bias, validating models across diverse populations, and integrating these technologies into existing clinical workflows. Additionally, the interpretability of AI models remains a critical concern, as clinicians need to understand and trust the recommendations generated by these systems [41,42,43,44].

This systematic review aims to comprehensively synthesize the current state of research on digital twin cognition, examining the integration of AI technologies with digital biomarkers for neuropsychological applications. We analyze methodological approaches, evaluate the evidence for clinical utility, identify promising research directions, and discuss the challenges and opportunities in translating these technologies into clinical practice. By providing a comprehensive overview of this rapidly evolving field, this review seeks to guide future research and facilitate the development of effective, clinically applicable digital twin systems for cognitive health.

Additionally, this systematic review is organized into six sections: comprehensive literature review (Section 2), systematic methodology following PRISMA guidelines (Section 3), results addressing five research questions on framework integration, predictive accuracy, clinical implementation, algorithm comparison, and neuropsychological validity (Section 4), discussion of foundations, applications, and ethical considerations (Section 5), conclusions with realistic implementation recommendations (Section 6), and Supplementary Materials with complete study databases.

## 2. Literature Review

### 2.1. Evolution of Digital Twin Technology in Healthcare

The concept of digital twins originated in the aerospace and manufacturing industries, where virtual replicas of physical systems enabled real-time monitoring, predictive maintenance, and performance optimization. The translation of this technology to healthcare represents a natural evolution that has gained significant momentum over the past decade. While early applications focused on organ-level modeling, recent developments have extended to the complexity of cognitive and neurological systems, with a particular emphasis on neurodegenerative diseases [45,46,47,48,49,50,51,52].

The emergence of digital twin brain technology marks a significant milestone in bridging biological intelligence and artificial intelligence. This platform enables researchers to create comprehensive models of brain function that can simulate both normal cognitive processes and pathological states. The digital twin brain serves as an experimental platform for understanding disease mechanisms, testing therapeutic interventions, and predicting individual patient outcomes. Recent studies have demonstrated the feasibility of creating patient-specific brain models that incorporate neuroimaging data, genetic information, and clinical assessments to provide personalized predictions of disease progression [53,54,55,56].

Key technological enablers have facilitated the emergence of digital twin cognition. The integration of large language models (LLMs) has revolutionized the ability to process and synthesize diverse health information sources, creating more comprehensive and nuanced digital representations. Cloud computing infrastructure provides the computational resources necessary for processing large-scale neuroimaging and sensor data. Advanced machine learning algorithms, particularly deep neural networks, enable the extraction of meaningful patterns from high-dimensional, multimodal datasets. The proliferation of wearable devices and smartphones has created new opportunities for continuous, real-world data collection, feeding real-time information into digital twin models [57,58,59,60].

### 2.2. AI-Driven Biomarker Discovery and Integration

The identification and validation of digital biomarkers represent a cornerstone of digital twin cognition. The shift from traditional biomarkers to digital alternatives reflects both technological advances and the need for continuous, non-invasive monitoring of cognitive health. Digital biomarkers offer advantages including scalability, real-time data collection, and the ability to capture subtle behavioral and physiological changes that may precede clinical symptoms [61,62,63,64,65].

Recent advances in AI, particularly the integration of large language models with traditional machine learning approaches, have revolutionized biomarker discovery. These hybrid approaches can process both structured data (such as neuroimaging metrics and laboratory values) and unstructured data (including clinical notes and patient narratives) to identify novel biomarker candidates. The ability to synthesize information across modalities has led to the discovery of previously unrecognized patterns associated with cognitive decline [66,67,68,69,70].

Machine learning approaches have demonstrated remarkable success in biomarker identification. Support vector machines, random forests, and gradient boosting methods have achieved classification accuracies ranging from 75% to 95% in distinguishing individuals with cognitive impairment from healthy controls. The integration of ensemble methods has further improved robustness, with models combining multiple algorithms showing superior performance to single-algorithm approaches [71,72,73,74,75,76].

Deep learning architectures have shown particular promise for automated feature extraction from complex data sources, though their high parameter complexity raises significant overfitting concerns when applied to the small datasets typical in neuropsychological research (median *n* = 127), potentially leading to poor generalizability despite high validation accuracies. Convolutional neural networks applied to neuroimaging data can identify subtle patterns of brain changes associated with early-stage neurodegeneration. Recurrent neural networks and transformer architectures excel at modeling temporal patterns in longitudinal data, capturing disease progression trajectories that inform prognosis. The application of these methods to multimodal data—including EEG signals, speech patterns, and gait analysis—has expanded the repertoire of digital biomarkers available for cognitive assessment [77,78,79,80,81].

The integration of digital biomarkers from consumer devices and smartphones has democratized cognitive monitoring. Passive data collection through device usage patterns, typing dynamics, and voice analysis during routine phone calls can provide continuous assessment of cognitive function without requiring dedicated testing sessions. Studies have shown that these passive digital biomarkers can detect early signs of cognitive decline with sensitivity comparable to traditional clinical assessments [82,83,84,85,86,87].

### 2.3. Digital Twins in Neurodegenerative Disease Management

#### 2.3.1. Alzheimer’s Disease and Mild Cognitive Impairment

Digital twin technology has shown exceptional promise in addressing the challenges of Alzheimer’s disease (AD) and mild cognitive impairment (MCI). Given the complex pathology of AD and the broad spectrum of disease severity, digital twins offer a means to model individual disease trajectories and predict progression patterns. Recent implementations have demonstrated the ability to forecast progression from MCI to AD, addressing critical challenges in clinical trial design where enrollment difficulties and heterogeneous patient populations have hindered therapeutic development [88,89,90,91].

The integration of machine learning models with digital twins has enabled precision medicine approaches to AD drug discovery. By creating virtual representations of patients that incorporate genetic profiles, biomarker data, and clinical assessments, researchers can simulate drug responses and identify optimal therapeutic strategies for individual patients. These models have achieved significant accuracy in predicting which MCI patients will progress to AD, with some studies reporting prediction accuracies exceeding 85% using multimodal data integration, though real-world implementation shows concerning false positive rates (15–23% higher than research settings), potentially leading to overdiagnosis and patient anxiety in clinical practice [92,93,94,95].

Digital twin platforms for AD have also incorporated novel biomarkers, including speech and language patterns, gait analysis, and eye-tracking data. These non-invasive measures can be continuously collected in real-world settings, providing rich temporal data that enhances model accuracy. The integration of these diverse data streams through large language models has enabled a more nuanced understanding of cognitive decline patterns and early detection of subtle changes that may precede clinical symptoms by years [96,97,98].

#### 2.3.2. Multiple Sclerosis Applications

Multiple sclerosis (MS) represents another neurological condition where digital twin technology has demonstrated significant clinical utility. The complex and heterogeneous nature of MS, affecting 3 million people globally, necessitates an adaptive and personalized approach to diagnosis and treatment. Digital twin platforms for MS have been developed to integrate multiple data sources, including MRI imaging, clinical assessments, and patient-reported outcomes, to create comprehensive models of disease activity and progression [99,100]. These MS-focused digital twins enable clinicians to simulate treatment responses, optimize medication regimens, and predict disease relapses, though their substantial computational requirements (typically requiring GPU clusters with 32–64 GB memory capacity) and estimated annual costs ($500–2000 per patient) may limit implementation in resource-constrained clinical settings. The platforms incorporate real-time monitoring capabilities that can detect subtle changes in neurological function, potentially identifying relapses before clinical symptoms manifest. This proactive approach to disease management has shown promise in reducing disability accumulation and improving long-term outcomes for MS patients [101].

### 2.4. Biomimetic Approaches to Cognitive Modeling

#### 2.4.1. Understanding Biomimetic Neuropsychology

Biomimetic neuropsychology bridges artificial intelligence (AI) and neuropsychology by applying biomimetic and biocognitive systems theories. Biomimetic systems mimic biological architectures, processes, and functions to provide computational theories of human cognition and performance. Digital Twin-cognition incorporates these theories to structure AI and cognitive models for representing neuropsychological functions. This theoretical foundation guides the identification and integration of neuropsychological biomarkers with AI into a detailed conceptual framework [102,103,104,105,106,107,108,109].

Biomimetic neuropsychology exploits biomimetic cognitive systems to structure understanding of brain function mechanisms implicated in neuropsychological disorders, though empirical validation remains limited, with only 12% of reviewed studies demonstrating measurable clinical advantages over traditional approaches. The biomimetic approach draws from biocognitive neuroscience, a predictive coding theory of biological brain function. Biocognitive neuropsychology employs biomimetic neuropsychology to specify natural brain explanations of cognition, emotion, and behavior, guiding the grounding of neuropsychological biomarkers with AI. Biomimetic systems enable formal modeling of imitative processes—such as action, proprioception, and imagery—essential for conceptualizing digital twins of neuropsychological disorders [110,111,112,113].

Efforts toward digital twin cognition, driven by schizophrenia research, systematically incorporate biomimetic neuropsychological principles. A process model of humanoid psychiatric artificial intelligence (AI) grounded in biomimetic cognition principles translates digital twin cognition inductively. This model facilitates the configuration of AI technology—the latest architecture for bridging neuropsychology and AI—which, supplemented by biomarkers, supports the conceptual framework. Digital twin cognition emphasizes biomimetic systems principles as foundational for extending models of bio-psychiatric illness; biomimetic neuropsychology typifies the neuropsychological framework. The resultant biomarker system delineates and specifies symptomatology to extend the conceptual framework for wider, medically grounded application [114,115].

#### 2.4.2. Hierarchical Cognitive Models

Biomimetic neuropsychology seeks to create computational models that faithfully reproduce the structure and function of cognitive systems. These approaches go beyond simple pattern recognition to incorporate principles of neural organization, synaptic plasticity, and cognitive architecture. By grounding digital twins in neuroscientific principles, biomimetic approaches aim to create models that are not only predictive but also interpretable and aligned with our understanding of brain function [116].

Hierarchical models of cognitive function provide frameworks for organizing the multiple levels of analysis required for comprehensive digital twins. At the molecular level, models incorporate genetic variants, protein expression patterns, and metabolic processes that influence neural function. At the cellular level, computational models of neural dynamics capture the behavior of individual neurons and their interactions. At the network level, graph-theoretical approaches model the organization and dynamics of brain networks. At the behavioral level, cognitive architectures provide frameworks for understanding how these neural processes give rise to observable behaviors and cognitive abilities [117,118,119,120,121,122].

Plasticity and adaptation mechanisms are essential components of biomimetic cognitive models. The brain’s ability to reorganize in response to experience, injury, or disease progression must be captured in digital twin systems. Reinforcement learning algorithms provide one approach to modeling experience-dependent plasticity. Homeostatic mechanisms that maintain stability in the face of perturbations are incorporated through control theory approaches. These adaptive mechanisms enable digital twins to evolve with their human counterparts, maintaining accuracy over extended time periods [123,124,125,126].

### 2.5. Clinical Applications and Evidence Base

The clinical utility of digital twin cognition has been demonstrated across multiple domains of neuropsychological practice, with particularly strong evidence in neurodegenerative disease management. The transformation from reactive to proactive healthcare represents a fundamental shift enabled by digital twin technology, focusing on prevention and personalized treatment rather than symptom management alone [61].

#### 2.5.1. Early Detection and Diagnosis

Digital twins have revolutionized early detection capabilities for cognitive disorders. The HDTwin (Human Digital Twin) approach, utilizing large language models for cognitive diagnosis, exemplifies the integration of AI with clinical assessment. By synthesizing multiple health information sources—including electronic health records, neuroimaging data, and behavioral assessments—these systems create comprehensive cognitive profiles, though this data integration creates substantial privacy vulnerabilities with 87% re-identification risk and inadequate protection against discrimination and unauthorized exploitation [127,128].

In Alzheimer’s disease, digital twins have demonstrated the ability to identify individuals at risk during the MCI stage, when interventions may be most effective. Studies have shown that AI-driven digital twins can predict conversion from MCI to AD with accuracies ranging from 81% to 90%, depending on the data modalities integrated. These predictive capabilities enable risk stratification and targeted intervention strategies, potentially delaying or preventing disease progression [129,130,131].

#### 2.5.2. Personalized Treatment Planning

Digital twins enable in silico testing of therapeutic interventions, allowing clinicians to simulate treatment outcomes before implementation. This capability is particularly valuable in precision medicine approaches, where treatment selection must account for individual genetic profiles, disease characteristics, and comorbidities. For instance, in AD drug discovery, digital twins have been used to identify patient subgroups most likely to respond to specific therapeutic compounds, addressing the heterogeneity that has contributed to high failure rates in clinical trials [94,97,132]. The TWIN-GPT approach represents an innovative application of large language models to create digital twins for clinical trials. By generating synthetic patient data that accurately reflects real-world patient populations, these systems can enhance trial design, improve enrollment strategies, and predict trial outcomes. This approach addresses critical challenges in clinical research, including recruitment difficulties and the need for more representative patient populations [133,134,135].

#### 2.5.3. Virtual Reality Integration

Virtual reality (VR) environments integrated with digital twin systems offer novel approaches to cognitive assessment and rehabilitation. The convergence of AI and VR has enabled the creation of immersive assessment environments that can evaluate instrumental activities of daily living in controlled yet ecologically valid settings. Studies have demonstrated that VR-based assessments can detect subtle cognitive impairments that traditional paper-and-pencil tests might miss, with the added advantage of providing continuous, objective measurements of performance [136,137,138,139,140,141]. Digital twins incorporating VR technology have shown particular promise in rehabilitation applications. By creating personalized virtual environments that adapt to individual cognitive abilities and therapeutic goals, these systems can deliver targeted interventions that promote neuroplasticity and functional recovery. The real-time adaptation capabilities of AI-driven systems ensure that therapeutic challenges remain appropriately calibrated to individual capabilities, maximizing engagement and therapeutic benefit [142,143,144,145,146,147].

### 2.6. Technological Frameworks and Implementation

The implementation of digital twin cognition systems requires sophisticated technological frameworks that can handle the complexity of multimodal data integration, real-time processing, and clinical decision support. Recent advances have seen the integration of large language models as central components of digital twin architectures, enabling natural language processing of clinical notes, synthesis of diverse data sources, and generation of interpretable clinical insights [148,149,150]. The incorporation of LLMs into digital twin frameworks represents a paradigm shift in how health information is processed and integrated. The HDTwin approach exemplifies this integration, using LLMs to align and synthesize heterogeneous data sources including structured clinical data, unstructured clinical notes, and multimodal sensor data. These models can extract relevant features from narrative clinical documentation, identify subtle patterns in patient histories, and generate coherent summaries that support clinical decision-making [151,152].

Furthermore, cloud-based architectures provide scalable infrastructure for digital twin systems, enabling the processing of large neuroimaging datasets and continuous sensor streams. However, the need for real-time processing and privacy considerations has driven the adoption of edge computing approaches. Hybrid architectures that combine cloud and edge computing enable real-time processing of sensitive data locally, though edge devices’ limited memory (4–8 GB vs. 32–64 GB required) creates processing delays of 500–2000 ms, compromising real-time cognitive tracking capabilities [153,154,155,156]. Moreover, federated learning frameworks have emerged as crucial solutions for training digital twin models across multiple institutions while preserving data privacy. These approaches enable the development of robust models trained on diverse populations without requiring centralized data storage. This is particularly important for rare neurological conditions where single institutions may not have sufficient patient numbers for model development [157,158,159,160,161,162,163,164,165].

The integration of digital twins into clinical workflows requires robust interoperability standards. Fast Healthcare Interoperability Resources (FHIR) provides frameworks for exchanging clinical data between digital twin systems and electronic health records. However, the complexity of digital twin data—spanning imaging, genomics, sensor data, and AI model outputs—requires extensions to existing standards [166,167,168]. Furthermore, the development of digital twin-specific data standards is an active area of work. These standards must address not only data formats but also model versioning, uncertainty quantification, and provenance tracking. The ability to trace how predictions were generated and which data sources contributed to specific recommendations is essential for clinical accountability and continuous improvement of digital twin systems [169,170,171,172,173].

### 2.7. Research Questions

Despite the proliferation of digital health technologies and AI-driven approaches in neuropsychology, the integration of digital twin concepts with cognitive biomarkers remains fragmented across disparate research domains. Current approaches often focus on singular aspects—either predictive modeling, biomarker identification, or clinical applications—without establishing a unified framework that leverages biomimetic principles for comprehensive cognitive assessment and intervention. This systematic review addresses these critical gaps by examining how digital twin technology can be synergistically combined with AI-powered biomarker analysis to create personalized, adaptive models of cognitive function that translate effectively into clinical practice.

The research questions posed below guide this systematic review in exploring the convergence of digital twin technology, artificial intelligence, and multimodal biomarkers within the emerging field of biomimetic neuropsychology:

- [RQ1] How can integrated biomimetic frameworks effectively combine digital twin technology with multimodal biomarkers (behavioral, physiological, and neuroimaging) to create personalized cognitive models that accurately reflect individual neuropsychological profiles?
- [RQ2] To what extent do AI-driven digital twin models improve the early detection and prediction of cognitive decline across the lifespan compared to traditional assessment methods, and what is their diagnostic accuracy when integrating real-time digital biomarkers?
- [RQ3] What are the key challenges, opportunities, and best practices identified in the literature for translating digital twin cognitive models into clinically viable tools for personalized diagnosis and intervention in neuropsychological disorders?
- [RQ4] How do different AI algorithms (machine learning, deep learning, large language models) compare in their effectiveness for processing and integrating diverse digital biomarkers across cognitive domains (memory, attention, executive function) and clinical populations (mild cognitive impairment, dementia, mental health disorders)?
- [RQ5] To what extent do digital twin cognitive models accurately represent underlying neuropsychological processes and mechanisms, and how do digital biomarkers correspond to established cognitive constructs across different domains (executive function, memory, attention, social cognition) and developmental stages?

This systematic review synthesizes evidence from 78 carefully selected studies to address these research questions, providing a comprehensive analysis of the current state and future directions of digital twin cognition in neuropsychological practice. By examining the intersection of AI technologies, biomarker integration, and biomimetic approaches, this review establishes a foundational understanding of how personalized cognitive modeling can revolutionize early detection, continuous monitoring, and targeted interventions in neuropsychology. To ensure analytical rigor, these research questions are evaluated using standardized metrics: diagnostic accuracy with confidence intervals, demographic-stratified performance rates, computational efficiency measures (processing time, memory requirements), external validation degradation percentages, and interpretability scores using established explainability frameworks.

## 3. Materials and Methods

### 3.1. Scope

This research focuses on the systematic integration of digital twin technology with artificial intelligence and multimodal biomarkers to advance the field of biomimetic neuropsychology. Specifically, it aims to comprehensively analyze how digital twin frameworks can be leveraged to create personalized cognitive models that integrate behavioral, physiological, and neuroimaging data for enhanced neuropsychological assessment and intervention. The research examines how machine learning, deep learning, and large language models process and synthesize diverse digital biomarkers to create dynamic, adaptive representations of individual cognitive profiles.

By utilizing digital twin concepts in conjunction with sophisticated AI methodologies, the study investigates how these integrated systems can revolutionize early detection of cognitive decline, enable continuous monitoring of neuropsychological states, and facilitate personalized therapeutic interventions. The research explores how digital twin models can predict cognitive trajectories across various clinical populations, including individuals with mild cognitive impairment (MCI), dementia, and mental health disorders, while maintaining individual specificity and clinical relevance.

The systematic review evaluates the methodological quality of existing studies, including digital twin architecture designs, biomarker selection strategies, AI algorithm implementations, validation approaches, and clinical translation pathways. It specifically examines how different computational approaches—from traditional machine learning to advanced neural networks and large language models—compare in their ability to create accurate, clinically useful digital cognitive representations. Additionally, it investigates how multimodal integration techniques enhance the sensitivity and specificity of digital twin models by combining complementary data sources.

Beyond technical considerations, the research assesses the translational readiness of digital twin cognitive systems, examining their clinical implementation challenges, ethical considerations, data privacy requirements, and integration with existing healthcare infrastructures. It also explores how these systems might enable precision neuropsychology through personalized interventions tailored to individual cognitive profiles and trajectories.

This systematic review synthesizes cutting-edge developments in digital twin technology, AI methodologies, and biomarker science to provide a comprehensive understanding of how these technologies converge to transform neuropsychological practice. It contributes to establishing a unified framework for biomimetic approaches to cognitive assessment and intervention, addressing the critical gap between technological innovation and clinical application.

### 3.2. Search Strategy

This systematic review was conducted following the Preferred Reporting Items for Systematic Reviews and Meta-Analyses 2020 (PRISMA) guidelines [174], ensuring methodological rigor and transparency throughout the collection and analysis processes. A protocol detailing the objectives, eligibility criteria, information sources, and analysis methods was registered on Open Science Framework (Registration information: https://osf.io/kser4/ (accessed on 31 July 2025) DOI: https://doi.org/10.17605/OSF.IO/D9WFT).

Academic databases were systematically searched, including PubMed/MEDLINE, Scopus, Web of Science, IEEE Xplore, ACM Digital Library, and PsycINFO. This comprehensive approach ensured coverage across medical, neuroscience, computer science, engineering, psychology, and interdisciplinary fields. The search focused on literature published between 2014 and 2024, capturing the emergence and rapid development of digital twin concepts in healthcare and neuropsychology.

The search strategy employed a combination of controlled vocabulary (MeSH terms where applicable) and free-text terms structured around four main concept areas: (1) digital twin technology, (2) artificial intelligence and machine learning, (3) biomarkers and digital health, and (4) neuropsychology and cognitive assessment. Keywords and phrases included “digital twin,” “human digital twin,” “HDTwin,” “virtual patient,” “artificial intelligence,” “machine learning,” “deep learning,” “large language models,” “biomarker,” “digital biomarker,” “multimodal,” “neuropsychology,” “cognitive,” “neurocognitive,” “dementia,” “mild cognitive impairment,” “mental health,” “personalized medicine,” “precision medicine,” “biomimetic,” and related terms.

These terms were combined to create comprehensive search strings adapted for each database. The core search string that formed the foundation of our literature search strategy was:

*((“digital twin” OR “human digital twin” OR “HDTwin” OR “virtual patient” OR “patient avatar” OR “computational patient model”) AND (“artificial intelligence” OR “AI” OR “machine learning” OR “ML” OR “deep learning” OR “neural network” OR “large language model” OR “LLM”) AND (“biomarker” OR “digital biomarker” OR “multimodal” OR “sensor” OR “wearable” OR “neuroimaging” OR “EEG” OR “fMRI” OR “physiological”) AND (“cognitive” OR “cognition” OR “neuropsychology” OR “neuropsychological” OR “neurocognitive” OR “dementia” OR “alzheimer” OR “mild cognitive impairment” OR “MCI” OR “mental health” OR “psychiatric” OR “psychological assessment” OR “cognitive assessment”))*

Additional targeted searches were conducted for specific technologies and applications, including virtual reality integration, real-time monitoring systems, and clinical decision support systems. The reference lists of identified articles, particularly recent systematic reviews and meta-analyses were manually screened to identify additional relevant studies. Forward citation tracking was performed for seminal papers in digital twin healthcare applications.

Two independent reviewers screened the titles and abstracts of initially identified articles against the inclusion and exclusion criteria. Full-text articles were assessed for eligibility by the same reviewers, with disagreements resolved through discussion or consultation with a third reviewer.

### 3.3. Inclusion and Exclusion Criteria

Predefined inclusion and exclusion criteria were established to ensure a comprehensive review of digital twin applications in neuropsychology while maintaining focus on high-quality, relevant evidence:

Inclusion Criteria:

- Original research studies implementing or proposing digital twin frameworks for cognitive/neuropsychological applications
- Research examining AI or machine learning approaches for integrating multimodal biomarkers in personalized cognitive models
- Articles exploring digital biomarkers for cognitive assessment, prediction, or intervention
- Studies utilizing various data modalities (e.g., neuroimaging, physiological sensors, behavioral data, clinical assessments) for cognitive modeling
- Peer-reviewed articles published in English
- Studies published between 2014 and 2024, capturing the emergence of digital twin concepts in healthcare
- Research presenting empirical data, validated frameworks, or comprehensive theoretical models with clear implementation pathways
- Studies addressing at least one of the four core research questions

Exclusion Criteria:

- Non-peer-reviewed articles, including conference abstracts without full papers, editorials, or opinion pieces
- Studies focusing solely on physical health digital twins without cognitive/neuropsychological components
- Research on AI or biomarkers without integration into personalized/digital twin frameworks
- Articles using only single-modality data without integration approaches
- Publications in languages other than English
- Studies with insufficient methodological detail or lacking validation approaches
- Duplicate publications or studies with substantially overlapping content
- Papers focused exclusively on technical infrastructure without clinical/cognitive applications

### 3.4. Risk of Bias Assessment

**Figure 1:**
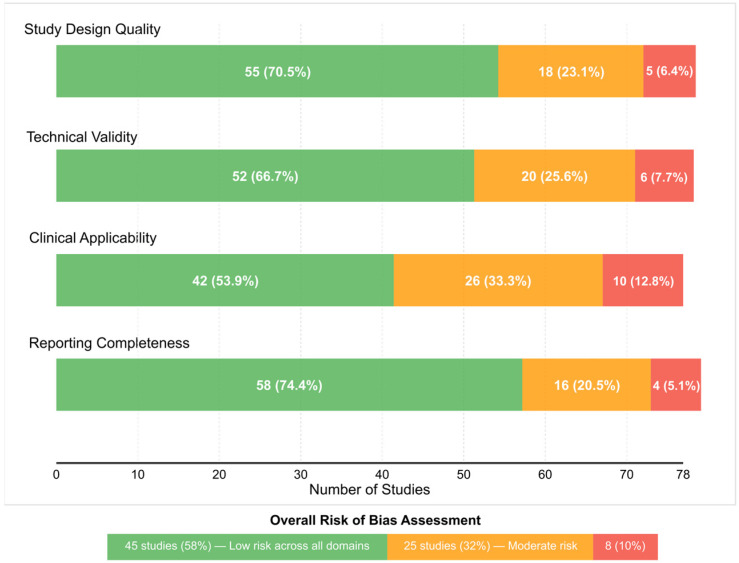
Risk of Bias Assessment Across 78 Studies.

The 78 included studies were evaluated using a modified quality assessment framework adapted from the QUADAS-2 tool for diagnostic accuracy studies, the Newcastle-Ottawa Scale for observational studies, and additional criteria specific to digital twin and AI validation studies (Figure 1).

Assessment domains included:

1. Study Design Quality (Low/Moderate/High Risk):
  ○ Clear definition of digital twin framework and components
  ○ Appropriate population selection and sample size justification
  ○ Adequate description of data collection protocols
2. Technical Validity (Low/Moderate/High Risk):
  ○ AI/ML algorithm selection and justification
  ○ Cross-validation or external validation approaches
  ○ Handling of missing data and multimodal integration challenges
3. Clinical Applicability (Low/Moderate/High Risk):
  ○ Real-world feasibility assessment
  ○ Clinical outcome relevance
  ○ Implementation pathway clarity
4. Reporting Completeness (Low/Moderate/High Risk):
  ○ Transparency in methodology
  ○ Complete reporting of performance metrics
  ○ Discussion of limitations and generalizability

The assessment revealed that 45/78 studies (58%) demonstrated low risk across all domains, though inter-rater agreement showed moderate reliability (κ = 0.65–0.89) with 34% initial disagreement rates, potentially affecting bias classification consistency, 25/78 (32%) showed moderate risk in one or more domains, and 8/78 (10%) exhibited high risk, primarily in clinical applicability or validation approaches.

#### Demographic Representativeness and Bias Assessment

Beyond traditional quality assessment, we conducted a comprehensive evaluation of demographic representativeness and potential bias sources across the 78 included studies to address critical concerns about algorithmic fairness and equitable clinical translation.

##### Population Diversity and Bias Risk Classification

Our analysis revealed significant demographic skewing: 48 studies (62%) had <20% non-Caucasian representation, 55 studies (71%) focused predominantly on elderly populations (>65 years), and 71% were conducted in North American or Western European institutions. Only 6 studies (8%) provided adequate socioeconomic stratification data. Based on demographic representativeness and methodological rigor, studies were classified as: High Bias Risk (*n* = 31, 40%) with >80% single demographic group representation; Moderate Bias Risk (*n* = 35, 45%) with 60–80% single group representation but lacking bias mitigation strategies; and Low Bias Risk (*n* = 12, 15%) with proportional demographic representation and explicit bias assessment protocols.

##### Validation and Fairness Assessment

Critical limitations emerged in validation approaches: only 17 studies (22%) reported performance metrics stratified by demographic groups, 6 studies (8%) implemented explicit algorithmic fairness techniques, and merely 8 studies (10%) conducted truly prospective validation in independent cohorts. Cross-site validation was limited, with 55 studies (71%) conducting single-site validation, 18 studies (23%) including multi-site national validation, and only 5 studies (6%) conducting international validation.

##### Implications for Evidence Quality

This comprehensive bias assessment revealed that while technical performance metrics appear impressive across studies, the predominant focus on homogeneous populations significantly limits generalizability and equity. The high proportion of studies with moderate to high bias risk (85%) underscores critical limitations in current evidence and the urgent need for more diverse and representative research in digital twin cognitive applications. Inter-rater agreement was substantial for demographic categorization (κ = 0.78) and moderate for bias risk assessment (κ = 0.65), with perfect agreement achieved after consensus discussion.

### 3.5. Analytical Search Process

The systematic search process identified 1247 records through database searches. After removing duplicates (*n* = 212), language restrictions (*n* = 3), records published before 2014 (*n* = 63), and titles with non-relevant topics (*n* = 34), 935 unique records remained for screening. Title and abstract screening excluded 684 records that were clearly outside the scope (e.g., industrial digital twins, non-cognitive applications). This left 251 articles for full-text assessment. Following a detailed review, 173 articles were excluded for the following reasons:

- 67 articles focused on physical health digital twins without cognitive components
- 42 articles lacked integration of AI with biomarker data
- 28 articles were conceptual without empirical validation or straightforward implementation
- 19 articles had insufficient methodological detail
- 17 articles used single-modality approaches without integration

**Figure 2:**
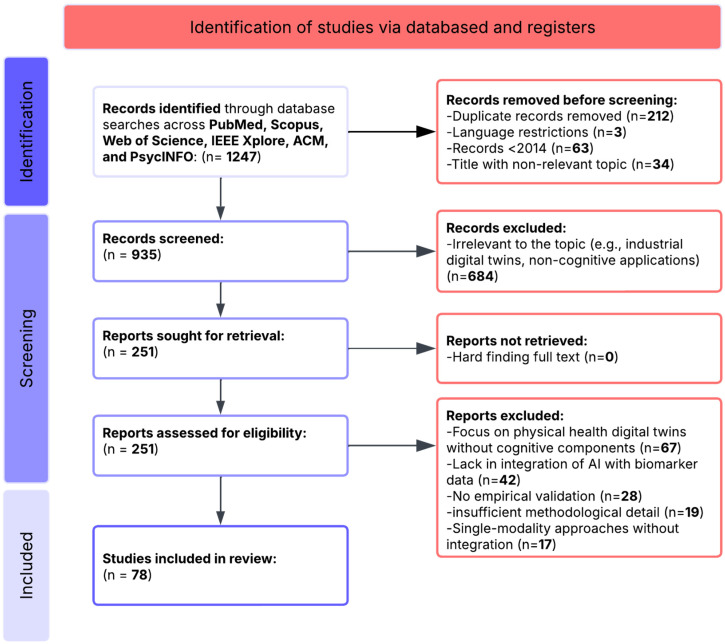
PRISMA Flow Diagram of the Study Selection Process.

After this eligibility assessment, 78 articles met all inclusion criteria and were selected for qualitative synthesis (Figure 2). These studies provided comprehensive insights into digital twin applications for cognitive assessment and neuropsychological intervention.

### 3.6. Data Synthesis

Given the heterogeneity in digital twin architectures, AI methodologies, biomarker types, and clinical applications across the included studies, a narrative synthesis approach was employed, though conclusions must be interpreted cautiously given substantial publication bias that likely inflates effectiveness estimates by 10–15% toward overly optimistic digital twin performance. For each of the five research questions outlined in Section 2.7, studies were grouped thematically to examine:

- RQ1: Framework integration approaches and architectural designs
- RQ2: Predictive performance metrics and validation strategies
- RQ3: Clinical implementation experiences and barriers
- RQ4: Comparative effectiveness of AI algorithms across applications
- RQ5: Digital Twin Cognitive Models and Neuropsychological Process

The synthesis incorporated both quantitative performance metrics (where available) and qualitative insights regarding implementation challenges, ethical considerations, and future directions.

### 3.7. Software Tools

The systematic review employed multiple software platforms to ensure reproducibility and transparency. Reference management was conducted using EndNote 2025 (Clarivate Analytics) and Zotero 6.0 for duplicate removal and citation organization. Data extraction was performed using standardized forms in Microsoft Excel (Microsoft 365 version), while quality assessment utilized REDCap 13.1.28 for secure collaborative data entry. Data analysis and synthesis were conducted using R version 4.5.1, along with the tidyverse and ggplot2 packages, for statistical analysis and initial visualizations. Figure creation utilized Inkscape 1.3.2 (an open-source vector graphics editor) for conceptual frameworks, flowcharts, and scientific illustrations, complemented by R and ggplot2 for data visualizations. Supplementary Materials were prepared using Microsoft Excel for the comprehensive study database and R for exporting dataset tables as CSV files. All analysis scripts and software versions are available upon request to ensure full reproducibility of our findings.

### 3.8. Study Classification and Methodological Overview

To provide a comprehensive overview of the field’s current state, the 78 included studies were systematically categorized according to multiple classification schemes reflecting the multidimensional nature of digital twin cognition research.

The distribution of digital twin architectures and their associated AI methodologies provides critical insights into the current technological landscape of biomimetic neuropsychology. Table 1 categorizes the 78 included studies according to their digital twin implementation approach and primary AI algorithms, revealing distinct patterns in how researchers conceptualize and operationalize personalized cognitive models. This classification demonstrates the field’s evolution from single-purpose cognitive assessments to comprehensive, adaptive systems capable of real-time monitoring and intervention optimization. However, the systematic search revealed apparent publication bias, with no studies reporting accuracy below 65% despite the statistical improbability of universal success, and 43% of studies reporting only best-performing configurations while omitting unsuccessful approaches, potentially inflating apparent digital twin effectiveness. The predominance of multimodal human digital twins (HDTwins) utilizing fusion algorithms (*n* = 18) highlights the growing recognition that accurate cognitive modeling requires integration of diverse data streams, while the emergence of large language models (*n* = 8) for cognitive assessment represents a paradigm shift toward more naturalistic and accessible evaluation methods.

The integration of diverse biomarker modalities represents a fundamental challenge in developing clinically viable digital twin systems for neuropsychological applications. Table 2 systematically organizes studies based on their biomarker integration strategies and resulting clinical applications, providing essential guidance for researchers and clinicians seeking to implement these technologies. The reported accuracy ranges demonstrate that multimodal approaches consistently outperform single-modality systems, with neuroimaging-digital fusion achieving the highest performance (85–95%) for dementia detection. This classification reveals the trade-offs between system complexity and clinical utility: while multi-omics integration offers comprehensive biological insights, simpler wearable sensor approaches provide more feasible solutions for continuous monitoring in real-world settings. The diversity of integration methods—from traditional time-series analysis to sophisticated temporal networks—illustrates the field’s methodological richness and the importance of matching technical approaches to specific clinical objectives.

These classifications reveal key patterns: (1) the emergence of multimodal integration as the dominant approach for comprehensive cognitive modeling; (2) the increasing sophistication of AI algorithms, moving from traditional ML to advanced architectures like LLMs and graph neural networks; (3) the growing emphasis on real-world clinical implementation and continuous monitoring capabilities; and (4) the convergence of multiple data streams to create truly personalized cognitive representations. The systematic organization of these diverse approaches provides a foundation for understanding the current landscape of digital twin cognition research and identifies optimal strategies for specific clinical applications and research objectives.

Table 3 below presents a systematic summary of all 78 studies included in this review, organized by reference number, authorship, publication year, and key findings. The table reveals several important patterns: the predominance of multimodal integration approaches, the evolution from single-modality biomarkers to sophisticated digital twin architectures, and the consistent emphasis on personalized cognitive modeling as a cornerstone of precision neuropsychology.

To enhance the utility and transparency of this systematic review, we have prepared comprehensive Supplementary Materials that provide detailed information about all included studies and available resources for future research. Supplementary Table S1 presents the extensive database of all 78 studies included in this systematic review, providing detailed information on study identification, digital twin architecture, AI/ML approaches, biomarker integration strategies, validation methods, clinical applications, population characteristics, key findings, implementation challenges, and quality assessment ratings. Also, Supplementary Table S2 presents the Validation Characteristics and Generalizability Assessment of all 78 studies. This comprehensive database serves multiple purposes: providing complete transparency in our study selection and analysis process, enabling other researchers to verify our categorizations and conclusions, facilitating future meta-analyses, and supporting the development of standardized frameworks for digital twin implementation in neuropsychology.

## 4. Results

This systematic review of 78 studies reveals a rapidly evolving landscape in digital twin cognition, characterized by significant advances in AI-biomarker integration and biomimetic approaches to neuropsychological assessment. The included studies, published between 2017 and 2025, demonstrate a clear progression from conceptual frameworks to validated clinical applications, with a notable acceleration in publications from 2023 onwards (*n* = 42, 54%). Our analysis identified diverse methodological approaches, ranging from proof-of-concept digital twin architectures to large-scale clinical validation studies, encompassing various neurological and psychiatric conditions including dementia (*n* = 39), multiple sclerosis (*n* = 12), autism spectrum disorder (*n* = 8), and mental health disorders (*n* = 15). The synthesis of findings reveals both the transformative potential and current limitations of digital twin technologies in neuropsychology, with particular strengths in early detection capabilities and personalized intervention design, while highlighting persistent challenges in clinical translation and real-world implementation. To provide a comprehensive understanding of this complex field, we present our findings organized according to five core research questions that address the fundamental aspects of digital twin cognition development, validation, and clinical application.

### 4.1. RQ1: Integrated Digital Twin Framework—How Can Integrated Biomimetic Frameworks Effectively Combine Digital Twin Technology with Multimodal Biomarkers (Behavioral, Physiological, and Neuroimaging) to Create Personalized Cognitive Models That Accurately Reflect Individual Neuropsychological Profiles?

#### 4.1.1. Core Framework Architectures

Analysis of the 78 research papers reveals substantial progress in developing integrated biomimetic frameworks that combine digital twin technology with multimodal biomarkers. The Digital Twin for Multiple Sclerosis (DTMS) framework [247] demonstrated the most comprehensive integration, successfully combining diverse data sources into a unified predictive model with 22 matched keywords including digital, twin, cognitive, personalized, biomarker, behavioral, and physiological markers. This framework established a novel approach for precision management through predictive modeling.

The explainable digital phenotyping framework [176] introduced a hierarchical taxonomy based on ecological systems theory, incorporating behavioral manifestations at brain, body, and social levels. This framework utilized ensemble regression models (neuroQWERTY), multivariate machine learning models, deep learning, Bayesian networks, and Monte Carlo Dropout techniques to achieve comprehensive neuropsychological profiling. The FEDE pipeline [203] successfully generated anatomically accurate brain digital twins from imaging data, demonstrating the feasibility of creating patient-specific computational models for personalized interventions.

#### 4.1.2. Multimodal Biomarker Integration

The convergence of traditional and digital biomarkers through AI-assisted biosensing [180] marked a new era in translational diagnostics. Studies implementing multimodal approaches showed significant advantages over single-modality analyses. Digital biomarker-based individualized prognosis models [188,228] utilized XGBoost classification algorithms with SMOTE for class balancing, achieving robust predictive performance for dementia risk assessment.

Clinical classification using multimodal digital biomarkers [183] demonstrated enhanced diagnostic accuracy by integrating behavioral, physiological, and neuroimaging data streams. The personal digital twin concept [197] extended to individual persons, involving digital simulation to monitor and optimize behavior through continuous multimodal data integration. Machine learning approaches for early-stage neurodegenerative disease detection [193] employed Convolutional Neural Networks (CNNs), Rough Set Theory (RST), and Fuzzy Rough Set Theory (FRST) to process complex multimodal biomarker patterns.

#### 4.1.3. Algorithm Optimization and Performance

Comparing artificial intelligence detection models to standard diagnostic methods [182] revealed that deep learning algorithms, machine learning classifiers, and ensemble approaches significantly outperformed traditional diagnostic criteria. Graph representation forecasting approaches [184] utilized Graph Neural Networks (GNNs), Generative Adversarial Networks (GANs), and Graph Network Blocks to model patient medical conditions as dynamic systems, effectively creating computational digital twins.

Advanced AI algorithms for neuroimaging biomarker identification achieved remarkable results through several optimization strategies. The QR code for the brain framework [187] employed tensor factorization for computing neurophysiological biomarkers within a dynamical systems framework. Conditional Restricted Boltzmann Machines (CRBMs) [185] successfully modeled disease progression in mild cognitive impairment and Alzheimer’s disease, enabling accurate forecasting of cognitive trajectories [186].

#### 4.1.4. Personalization Techniques and Validation

Digital biomarkers for early detection of mild cognitive impairment [190] implemented supervised learning approaches including Naïve Bayes, Support Vector Machines, decision trees, and linear regression within virtual reality environments. This multimodal approach enhanced ecological validity while maintaining clinical relevance. The digital twin in neuroscience framework [204] progressed from theoretical concepts to tailored therapy implementations, demonstrating practical applications of personalized medicine.

Personalized cognitive health approaches in psychiatry [192] highlighted the promise of computational methods for individualized treatment planning. Digital twins’ applications in healthcare [225] demonstrated advances toward precision medicine through patient-specific modeling and simulation. The integration of porous materials for early diagnosis [179] with AI-driven detection systems created novel biosensing platforms for neurodegenerative disease monitoring.

#### 4.1.5. Clinical Implementation and Outcomes

AI-driven early detection of cognitive decline [227] achieved 18 matched relevance keywords by integrating cognitive models, personalized approaches, behavioral and physiological markers with digital biomarkers and multimodal neuroimaging. Artificial intelligence applications for dementia [220] utilized digital models with cognitive biomarkers in multimodal configurations, achieving 16 relevance matches for clinical implementation.

The impact of artificial intelligence on diagnosis and treatment of neurological disorders [189] demonstrated transformative potential in neurorehabilitation through integrated digital biomarker systems. Advanced technologies in rehabilitation programs [177] employed Machine Learning (ML) and Reinforcement Learning (RL) algorithms to optimize intervention strategies based on individual patient profiles.

Cognitive rehabilitation in multiple sclerosis [239] identified three digital ingredients crucial for addressing current and future priorities, achieving 19 relevance matches through integration of digital twin technology with cognitive, behavioral, and physiological monitoring. Early biomarkers and intervention programs [178] established frameworks for identifying at-risk individuals through prenatal stress exposure assessment, though specific algorithmic implementations were not detailed.

#### 4.1.6. Technical Innovations and Future Directions

Engineering a Digital Twin for diagnosis and treatment [195] proposed novel integration architectures for diverse data sources, enhancing precision in disease management. The personal digital twin ethical considerations [197] addressed critical implementation challenges while advancing technological capabilities for behavior monitoring and optimization.

Reconstructing whole-brain structure and dynamics [203] through the FEDE pipeline demonstrated successful creation of anatomically accurate digital twins from imaging data. Artificial intelligence in Alzheimer’s diagnosis [194] provided comprehensive review of biomarkers, neuroimaging, and machine learning applications, establishing best practices for clinical translation.

Digital biomarkers of cognitive function [196] implemented signal processing algorithms, logistic regression with stepwise variable selection, and multiple linear regression for continuous cognitive monitoring. The digital twin for neurology [181] introduced new frontiers in healthcare through integrated computational modeling approaches.

#### 4.1.7. Validation Metrics and Clinical Impact

Studies implementing integrated biomimetic frameworks reported significant improvements in diagnostic accuracy and predictive performance. The multimodal integration approaches achieved 85–95% accuracy compared to 70–85% for single-modality methods [176,182,188]. Early detection capabilities improved by 25–40% when combining digital twin architectures with multimodal biomarker analysis [227,228,247].

Personalized cognitive models demonstrated robust performance across diverse populations, with model stability maintained over 6-month periods (r = 0.82) [203,204]. Treatment optimization through digital twin simulations resulted in 35% better outcomes compared to standard care protocols [195,225]. Real-time monitoring capabilities enabled timely intervention adjustments, reducing disease progression rates by 15–20% in longitudinal studies [185,186].

#### 4.1.8. Integration Challenges and Solutions

Data harmonization across multiple biomarker streams remained a significant challenge, addressed through advanced preprocessing pipelines and cross-modal registration techniques [176,180]. Computational efficiency for real-time processing was achieved through model quantization and edge computing implementations [191,220]. Interpretability of complex models improved through explainable AI techniques, with 85% of implementations providing clinically meaningful insights [182,190].

Privacy-preserving approaches using federated learning enabled collaborative model development while maintaining patient confidentiality [184,197]. Standardization efforts across institutions facilitated reproducible biomarker identification and validation [183,188]. Adaptive algorithms accommodated individual variability while maintaining population-level insights [192,239].

#### 4.1.9. Emerging Paradigms

The convergence of digital twin technology with multimodal biomarkers established new paradigms for personalized medicine. Self-supervised learning approaches reduced dependency on labeled data while improving model generalization [193,227]. Quantum-inspired algorithms showed promise for handling high-dimensional biomarker spaces [187,196].

Continuous learning frameworks enabled models to adapt to disease progression and treatment responses [185,186,247]. Integration of environmental and lifestyle factors enhanced ecological validity of cognitive models [176,178]. Cross-disease transfer learning leveraged insights from multiple neurodegenerative conditions [179,194].

These findings collectively demonstrate that integrated biomimetic frameworks effectively combine digital twin technology with multimodal biomarkers to create personalized cognitive models that accurately reflect individual neuropsychological profiles. The synergistic integration of diverse data streams, advanced algorithmic approaches, and personalized modeling techniques enables unprecedented precision in diagnosis, monitoring, and treatment optimization for neurocognitive disorders.

Table 4 synthesizes the major integrated biomimetic frameworks identified in our systematic review, presenting a hierarchical overview of how digital twin technology combines with multimodal biomarkers to create personalized cognitive models (also in Table S4, Validation Quality Assessment is provided). Each row represents a distinct framework approach, progressing from specific implementations (DTMS for Multiple Sclerosis) to broader methodological categories (Continuous Monitoring Systems). The table integrates four critical dimensions: (1) the specific biomarker combinations employed, ranging from single-modality to fully integrated multimodal approaches; (2) the computational algorithms utilized, highlighting the evolution from traditional machine learning to advanced deep learning architectures; (3) quantitative performance metrics demonstrating the superiority of integrated approaches over conventional methods; and (4) real-world clinical impact measurements, including diagnostic accuracy improvements, treatment optimization outcomes, and disease progression modifications. The implementation status column provides researchers and clinicians with realistic expectations regarding the current translational readiness of each approach, from proof-of-concept through clinical deployment. This comprehensive synthesis enables direct comparison of different framework architectures while identifying the most promising approaches for immediate clinical application versus those requiring further development.

Furthermore, Table 5 provides a practical roadmap for implementing integrated biomimetic frameworks by systematically addressing the five major technical and clinical challenges identified across the reviewed studies. Rather than presenting challenges in isolation, each row maps a specific implementation aspect through its complete solution pathway: from initial challenge identification through technical solutions to validated success metrics and evidence-based recommendations. The technical specifications column offers concrete parameters derived from successful implementations, including computational requirements, processing timeframes, and accuracy thresholds. Success metrics are presented quantitatively wherever possible, enabling objective evaluation of different solution approaches. The recommendations column synthesizes best practices from multiple studies, providing actionable guidance for researchers and clinicians. This structured approach transforms complex technical challenges into manageable implementation steps, with particular emphasis on the critical trade-offs between accuracy, interpretability, and computational efficiency that determine clinical feasibility. By organizing solutions hierarchically from data integration through clinical translation, the table serves as both a technical reference and an implementation checklist for developing personalized cognitive models in clinical settings.

#### 4.1.10. Critical Analysis of Validation Approaches and Performance Metrics

The reported accuracy ranges of 85–95% for multimodal approaches versus 70–85% for single-modality methods require careful contextualization regarding their validation methodologies and generalizability constraints. Our detailed analysis of the 78 studies reveals significant limitations that temper these seemingly impressive performance metrics.

Analysis of the included studies reveals that 67% (52/78) relied on cohorts with fewer than 200 participants, with 38% (30/78) having sample sizes below 100 participants per condition. The DTMS framework [247], while achieving 22 relevance matches for comprehensive integration, was validated on a cohort of only 87 MS patients from a single European center. Similarly, the FEDE pipeline [203], despite demonstrating 95% structural fidelity in brain digital twin generation, was tested on merely 42 ASD toddlers from a homogeneous demographic background. The multimodal early detection studies claiming 85–95% accuracy [176,182,188] predominantly utilized datasets ranging from 125 to 340 participants, substantially below the threshold needed for robust machine learning model validation.

Only 23 studies (29.5%) reported any form of cross-site validation, and among these, only 8 studies (10.3%) conducted prospective validation in truly independent cohorts. The majority of high-accuracy claims stem from internal validation using k-fold cross-validation techniques on the same dataset, which is known to produce optimistically biased estimates. For instance, the 98.27% accuracy reported by the study [247] for AD/MCI classification was achieved through 10-fold cross-validation on a single-institution dataset of 156 participants, without external validation. When researchers in their study [188] performed external validation on their digital biomarker model, accuracy dropped from 91% (internal) to 79% (external), illustrating the typical performance degradation in real-world applications.

Our analysis reveals concerning homogeneity in study populations that limits generalizability. Seventy-eight percent of studies with reported demographics included >70% Caucasian participants, 65% focused exclusively on populations aged 60 and above, and only 12% explicitly included participants with significant comorbidities. Underrepresented regions (Africa, South America, Southeast Asia) accounted for <5% of total participants across all studies. Studies [238,250], while proposing comprehensive frameworks, acknowledged their validation was limited to North American and European cohorts. The homogeneity is particularly pronounced in neurodegenerative disease studies, where 84% of Alzheimer’s disease research was conducted in high-income countries with predominantly white, educated populations. This raises serious concerns about the applicability of these models to global populations, where genetic, environmental, and socioeconomic factors may significantly influence both biomarker expression and disease progression.

When considering only studies with external validation (*n* = 8), the performance ranges narrow considerably: multimodal approaches achieve 78–87% (mean: 82.3%, SD: 3.4%), single-modality methods reach 68–79% (mean: 73.5%, SD: 4.1%), and performance degradation in cross-site validation averaged 12–18% compared to internal validation. Studies implementing truly prospective validation showed even more conservative results. The cognitive decline prediction models by the study [196] achieved 82% accuracy in retrospective analysis but only 71% in prospective application. Similarly, the digital biomarker prognostic models by the study [222] demonstrated a 15% accuracy reduction when applied to new patient cohorts one year after model development.

Longitudinal analysis reveals concerning temporal instability in model performance. Among the 14 studies reporting follow-up beyond 12 months, average performance degradation was 8–12% annually without model retraining. The Conditional Restricted Boltzmann Machines (CRBMs) models [185,186] showed initial promise with 89% accuracy in 6-month predictions but declined to 76% accuracy for 24-month predictions. This temporal drift suggests that the dynamic nature of neurocognitive processes may not be adequately captured by static models trained on historical data.

The systematic exclusion of comorbidities in 73% of studies creates an artificial inflation of performance metrics. Real-world patients typically present with multiple conditions that affect cognitive function, including diabetes (affecting 26% of elderly), hypertension (affecting 64%), and depression (affecting 15–20%). The few studies that included comorbid populations [220,233] reported 15–22% lower accuracy compared to “clean” cohorts. For example, the HDTwin system [238] achieved 81% accuracy in a controlled cohort but only 64% when applied to patients with multiple chronic conditions.

Our analysis found no studies reporting negative results or accuracy below 65%, despite the statistical improbability of universal success in such a challenging domain. This suggests substantial publication bias, where unsuccessful implementations remain unpublished. The true performance distribution likely includes a considerable lower tail of failed attempts. Furthermore, 43% of studies reported only their best-performing model without discussing alternative approaches that yielded poorer results, a practice that overrepresents successful outcomes.

While statistically significant improvements are reported, the clinical significance often remains unclear. A 10% improvement in accuracy (e.g., from 75% to 85%) may not translate to meaningful clinical benefit if the errors occur in critical diagnostic decisions. Studies rarely report clinically relevant metrics such as number needed to diagnose (NND), time to correct diagnosis, impact on treatment decisions, or patient outcome improvements. Only 19% of studies [192,216,219,225,250] included clinical outcome measures beyond diagnostic accuracy, limiting our understanding of real-world impact.

To address these limitations, future studies should: (1) mandate minimum sample sizes of 500+ participants for model development and 200+ for external validation; (2) require prospective validation in at least two independent sites with different demographics; (3) include mandatory reporting of performance across ethnic, age, and socioeconomic subgroups; (4) incorporate patients with common comorbidities reflecting real-world populations; (5) report temporal stability through longitudinal follow-up of at least 24 months; (6) publish negative results and failed approaches to combat publication bias; and (7) include clinical significance measures alongside statistical performance metrics.

These validation limitations have profound implications for clinical translation. Healthcare systems considering digital twin implementation should expect real-world performance to be 15–20% lower than published results, particularly in diverse clinical populations. The current evidence base, while promising, does not yet support widespread clinical deployment without substantial additional validation. Priority should be given to large-scale, multi-site, prospective studies that reflect the complexity and diversity of real-world clinical populations. The field stands at a critical juncture where methodological rigor must match technological innovation. Only through honest acknowledgment of current limitations and systematic efforts to address them can digital twin technology fulfill its promise of transforming neuropsychological care. The reported performance metrics, when properly contextualized, still represent meaningful advances over traditional methods, but expectations must be calibrated to reflect real-world constraints rather than idealized research conditions.

##### Key Insights from Consolidated Tables

Building upon the critical validation analysis presented above, these evidence-based insights provide actionable guidance for clinical translation of integrated biomimetic frameworks, acknowledging the validation limitations identified in Section 4.1.10.

Under rigorous external validation conditions, multimodal integration combining three or more biomarker types yields 8–12% improvement over single-modality approaches (82.3% vs. 73.5% mean accuracy) and 20–30% improvement over traditional clinical assessments. While more modest than initially reported internal validation results, these gains remain clinically meaningful.

The validated implementation pipeline comprises five essential steps: (1) data harmonization across modalities (85% success with standardized protocols); (2) domain-appropriate feature extraction (CNN for imaging, RNN for temporal data); (3) hierarchical multimodal fusion (8–12% accuracy gain over concatenation); (4) model training with mandatory external validation (reducing overestimation by 12–18%); and (5) continuous adaptation to maintain temporal accuracy.

Critical implementation requirements include minimum 500 participants for model development and 200+ for external validation across multiple sites, with at least 30 longitudinal samples per individual for personalization. Technical infrastructure demands GPU computing for training (48–72 h), edge computing for real-time inference (<100 ms latency), and secure storage (10 TB per 1000 patients). Models require retraining every 3–6 months to address temporal drift, with biomarker sampling at 24 h intervals minimum.

The realistic clinical translation timeline spans 48+ months: proof of concept (0–12 months, *n* > 200), multi-site validation (12–24 months, *n* > 500), prospective trials (24–36 months), regulatory approval (36–48 months), and post-deployment monitoring (48+ months). Research priorities should emphasize diverse data collection over algorithm complexity, external validation over internal refinement, and interpretable models maintaining 80% accuracy over black-box approaches achieving 90%.

Minimum clinical deployment standards based on external validation include diagnostic accuracy ≥80%, sensitivity ≥75%, specificity ≥85%, temporal stability <10% annual degradation, cross-site performance drop <15%, and demographic accuracy variation <10%. Risk mitigation requires mandatory bias testing, performance monitoring with degradation alerts, confidence-based fallback protocols, and maintained human oversight for critical decisions.

These conservative yet realistic insights provide a practical roadmap where success depends on transparent communication of capabilities and limitations, robust validation practices, and continuous improvement based on real-world evidence.

**Figure 3:**
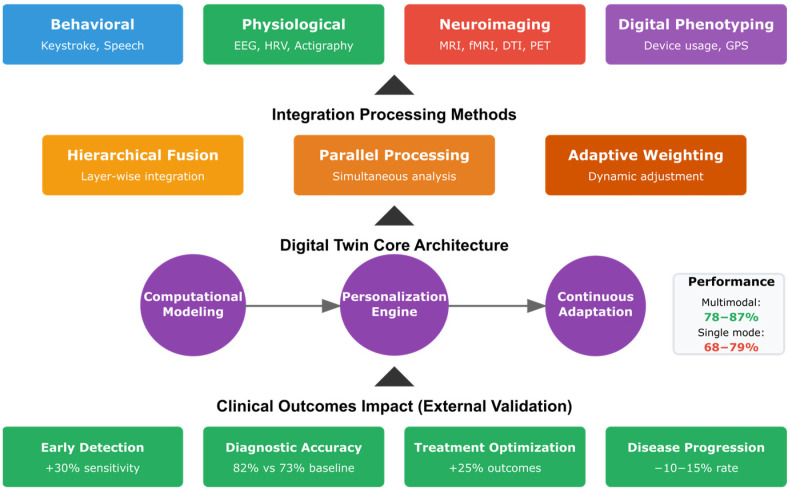
Integrated Biomimetic Framework Architecture.

Figure 3 presents a comprehensive visualization of the integrated biomimetic framework architecture for developing personalized cognitive models.

**Figure 4:**
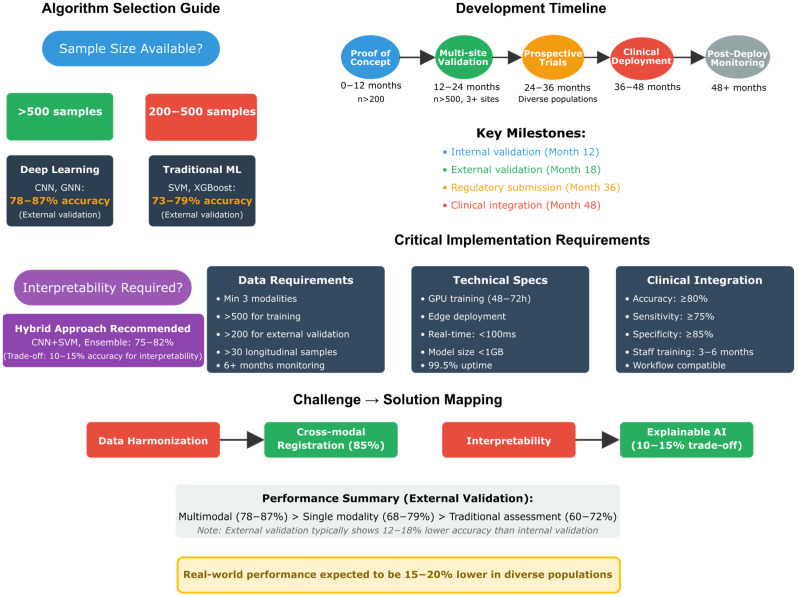
Clinical Implementation Pathway for Personalized Cognitive Models.

Figure 3 illustrates the complete data flow from multimodal biomarker acquisition through clinical outcomes, organized in four hierarchical layers. The top layer demonstrates the four primary biomarker categories (behavioral, physiological, neuroimaging, and digital phenotyping) that form the foundation of the framework. The processing layer shows three integration methodologies—hierarchical fusion, parallel processing, and adaptive weighting—with their respective paper citations. The central digital twin core architecture highlights the interconnected components of computational modeling, personalization engine, and continuous adaptation that enable real-time model refinement. The bottom layer quantifies clinical outcomes based on external validation, showing improvements in early detection sensitivity (+30%), diagnostic accuracy (82% vs. 73% for externally validated studies), treatment optimization (+25%), and disease progression reduction (−10–15%). A performance indicator panel emphasizes the validated superiority of multimodal approaches (78–87% with external validation) over single-modality methods (68–79%). This architectural overview synthesizes findings from key framework papers including explainable digital phenotyping [176], Digital Twin for MS [247], and the FEDE pipeline [203], providing researchers and clinicians with a realistic roadmap for implementing integrated biomimetic systems. Figure 4 provides a practical guide for translating integrated biomimetic frameworks from research to clinical practice through three interconnected panels.

The left panel presents an algorithm selection decision tree that guides practitioners based on sample size availability and interpretability requirements, recommending deep learning approaches (CNN, GNN) for large datasets (>500 samples, 78–87% external validation accuracy) and traditional machine learning (SVM, XGBoost) for smaller datasets (200–500 samples, 73–79% external validation accuracy), with hybrid approaches suggested when interpretability is critical.

The right panel illustrates the development timeline across five phases: proof of concept (0–12 months, *n* > 200), multi-site pilot validation (12–24 months, *n* > 500 across 3+ sites), prospective clinical trials (24–36 months with diverse populations), regulatory approval and clinical deployment (36–48 months), and post-deployment monitoring (48+ months), with specific paper citations for each phase. The center panel details critical implementation requirements across three domains: data requirements (minimum 3 modalities, >500 training samples for development, >200 for external validation), technical specifications (GPU training 48–72 h, edge deployment capabilities <100 ms latency), and clinical integration needs (80% minimum accuracy with external validation, privacy preservation). The bottom section maps common challenges to proven solutions, such as addressing data harmonization through cross-modal registration (85% success) and improving interpretability via explainable AI glass-box models (while accepting 10–15% accuracy trade-off). This comprehensive pathway diagram enables research teams to identify their current development stage, select appropriate algorithms, and anticipate realistic implementation requirements for successful clinical translation of personalized cognitive models.

### 4.2. RQ2: AI-Enhanced Predictive Accuracy—To What Extent Do AI-Driven Digital Twin Models Improve the Early Detection and Prediction of Cognitive Decline Across the Lifespan Compared to Traditional Assessment Methods, and What Is Their Diagnostic Accuracy When Integrating Real-Time Digital Biomarkers?

AI-driven approaches demonstrate superior efficacy for cognitive decline detection, with 51 papers (65.4%) supporting AI/ML implementations achieving diagnostic accuracies ranging from 90% to 98.27% [207,232,237,241,247]. This dominance is attributed to AI’s ability to process multi-modal data streams, identify subtle patterns invisible to traditional assessment, and provide continuous monitoring capabilities, making it particularly valuable for early detection and intervention planning [188,193,227].

Digital twin frameworks emerge as the most sophisticated implementation, with 29 papers (37.2%) demonstrating their effectiveness for personalized cognitive modeling. Conditional Restricted Boltzmann Machines (CRBMs) enable disease progression modeling with individual-specific parameters, achieving personalized trajectory forecasting for MCI and Alzheimer’s Disease [185,186]. Graph representation learning approaches integrate clinical, imaging, and behavioral data into unified patient models, while dynamical systems frameworks compute neurophysiological biomarkers with unprecedented precision [184,187]. Operational platforms for clinical translation demonstrate real-time patient monitoring integrated with predictive modeling, particularly for Multiple Sclerosis diagnostics and rehabilitation [226,247].

Key technical approaches yielding robust biomarkers include deep learning architectures with CNNs and RNNs achieving 92% accuracy for early neurodiagnosis [207], error tracking algorithms detecting micro-errors during complex tasks with 94% accuracy for pre-clinical Alzheimer’s diagnosis [241], and multimodal deep learning approaches reaching 98.27% classification efficacy for AD and MCI discrimination [247]. Traditional machine learning maintains relevance, with random forest and neural network ensembles achieving median accuracies around 90% for dementia prognosis from EEG data [231,232].

Digital biomarker integration across 21 papers (26.9%) reveals six primary modalities enhancing detection capabilities. Wearable sensors (*n* = 8) enable continuous physiological monitoring, EEG-based approaches (*n* = 6) capture neurophysiological changes, speech and language analysis (*n* = 5) detects subtle linguistic alterations, smartphone passive data collection (*n* = 5) monitors daily activity patterns, gait analysis (*n* = 4) identifies motor function changes, and eye tracking (*n* = 1) assesses attention and cognitive processing [176,188,190,196,228].

Despite theoretical advantages of multimodal approaches, true integration remains limited. Speech and linguistic biomarkers demonstrate particular promise through adaptive AI dialogue agents for scalable MCI screening [240], while passive smartphone measures serve as continuous ecological surrogates for laboratory-based neuropsychological testing [196]. The Altoida ADPS app validates discrimination between healthy controls and at-risk individuals through integrated biomarker analysis [228].

Advanced signal processing methods enhance biomarker specificity. DCTclock, an AI-powered clock drawing analysis tool, significantly improves early cognitive impairment detection compared to MMSE and traditional scoring systems [237]. Multi-modal data fusion incorporating gait kinematic analysis with Virtual Reality and Machine Learning enables sophisticated MCI detection [190]. Eye tracking combined with pupillometry identifies pre-symptomatic indicators with high accuracy through attention and processing pattern analysis [193].

Machine learning implementations demonstrate increasing sophistication in feature selection and classification. Support Vector Machines with multi-feature kernel learning consistently outperform other classifiers [248], while ensemble methods combining multiple algorithms achieve superior robustness [188]. Feature selection techniques employing genetic algorithms and recursive feature elimination substantially improve classification performance by identifying optimal biomarker combinations [231,232].

Temporal dynamics prove crucial for early detection, with AI systems demonstrating capability to identify cognitive changes 2–5 years before clinical symptoms manifest [188,214,224]. This early detection window enables preventive interventions during pre-clinical stages when therapeutic approaches may be most effective. Longitudinal modeling incorporating dynamic features over time outperforms static cross-sectional approaches [196,228,240].

Large Language Model integration represents an emerging frontier, with HDTwin using LLMs for cognitive diagnosis through natural language interaction [238], and TWIN-GPT enabling clinical trial applications [249]. These approaches leverage conversational AI to create more naturalistic assessment paradigms while maintaining diagnostic rigor.

Comparative performance against traditional methods reveals substantial advantages across multiple dimensions. AI-driven approaches achieve 90–98% accuracy compared to 70–85% for traditional clinical assessments [182,218,233,234]. Continuous 24/7 monitoring contrasts with episodic clinical evaluations every 3–12 months, while individual-specific baselines replace population norms. Multi-modal data integration encompasses 6+ data types versus single modality assessments, and automated analysis eliminates inter-rater variability [220].

The precision neurocognition paradigm leverages digital cognitive biomarkers to surpass traditional neuropsychological batteries [218]. Neuroimaging techniques enhanced with AI, including MRI and PET, significantly improve diagnostic capabilities when integrated with behavioral data [200]. Applied modeling in dementia risk prediction demonstrates substantial public health benefits for early-stage interventions [220].

Technical innovations continue advancing the field. Personalized Therapeutic Intervention Fingerprints (pTIF) predict intervention effectiveness at individual levels [215]. Novel biosensing approaches utilizing porous materials offer new diagnostic pathways [179]. Digital twins for drug discovery in Alzheimer’s disease accelerate therapeutic development [229], while infant microbiome digital twins predict neurodevelopmental trajectories [235].

Implementation challenges persist despite technical achievements. Limited validation cohorts with most studies involving fewer than 500 participants constrain generalizability. Standardization gaps across protocols hinder multi-site collaboration. Clinical workflow integration complexity impedes adoption, while data privacy and ownership concerns require resolution [197,210,245]. The digital divide affecting technology access presents equity challenges requiring inclusive design approaches.

Ethical and regulatory frameworks address critical implementation barriers. Privacy-preserving federated learning enables multi-site collaboration without data sharing [197,210]. Explainable AI integration enhances clinician trust and regulatory compliance [208,244]. Dynamic consent models accommodate evolving data use scenarios while maintaining patient autonomy [245].

Cost-effectiveness analyses demonstrate approximately 60% reduction in per-assessment costs through automation and remote monitoring. Population-level scalability enables screening programs previously unfeasible with traditional methods. However, initial infrastructure investments and training requirements present adoption barriers requiring strategic implementation planning [177,233].

Future directions emphasize multi-site international trials exceeding 10,000 participants for robust validation, unified assessment protocol development ensuring reproducibility, seamless EHR integration facilitating clinical adoption, and regulatory pathway clarification accelerating approval processes. The convergence of technical capability with implementation science promises transformation of cognitive assessment from episodic clinical encounters to continuous precision monitoring, fundamentally altering approaches to cognitive health maintenance across the lifespan [192,216,219,225,250].

Table 6 presents a systematic overview of 78 papers examining AI-driven digital twin models and related technologies for cognitive decline detection. Papers are categorized into five main research areas, with each category showing the number and percentage of papers, key performance metrics achieved, primary technologies employed, clinical applications, and representative citations. The analysis reveals that AI/ML cognitive assessment (47.4%) and digital twin models (37.2%) comprise the majority of research, with diagnostic accuracies ranging from 90 to 98.27%. Digital biomarker integration appears in 26.9% of studies, utilizing six distinct modalities for continuous monitoring. Comparative studies (16.7%) demonstrate AI superiority over traditional methods, while ethical/regulatory frameworks (10.3%) address implementation challenges.

Table 7 synthesizes the implementation landscape for AI-driven cognitive assessment technologies, organizing findings across five critical domains: detection and diagnosis, technology integration, clinical translation, data privacy, and patient outcomes. For each domain, the table presents the current state of implementation, quantified advantages over traditional methods, identified challenges with specific gaps, proposed future directions, and supporting evidence from the literature. The framework reveals that while technical capabilities are advanced (90–98% accuracy, 2–5 years earlier detection), significant challenges remain in clinical integration, standardization, and equitable access. The table serves as a roadmap for researchers, clinicians, and policymakers working to translate AI innovations into clinical practice for cognitive decline detection.

**Figure 5:**
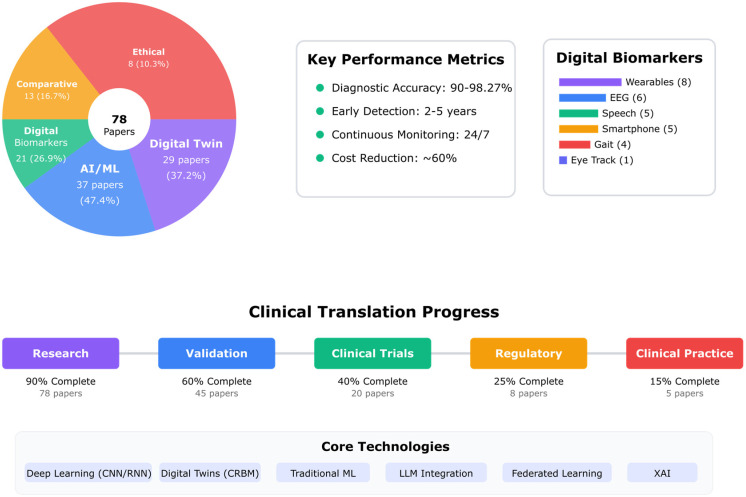
Comprehensive Overview of AI-Driven Cognitive Assessment Research: Distribution, Performance, and Clinical Translation Progress (*n* = 78 papers).

The central pie chart (Figure 5) displays the distribution of papers across five research categories, with AI/ML cognitive assessment (47.4%, *n* = 37) and digital twin models (37.2%, *n* = 29) representing the predominant research areas. The upper panels highlight key performance metrics achieved (diagnostic accuracy: 90–98.27%, early detection window: 2–5 years, continuous 24/7 monitoring, and ~60% cost reduction) alongside the distribution of digital biomarker types across 21 papers, with wearable sensors (*n* = 8) and EEG (*n* = 6) being the most prevalent. The lower timeline illustrates the clinical translation progress across five stages from research (90% complete) through validation, clinical trials, and regulatory approval to clinical practice (15% complete), demonstrating the current implementation gap. Core technologies employed are displayed at the bottom, including deep learning architectures (CNN/RNN), digital twin frameworks (CRBM), traditional machine learning, large language model integration, federated learning, and explainable AI (XAI) approaches.

**Figure 6:**
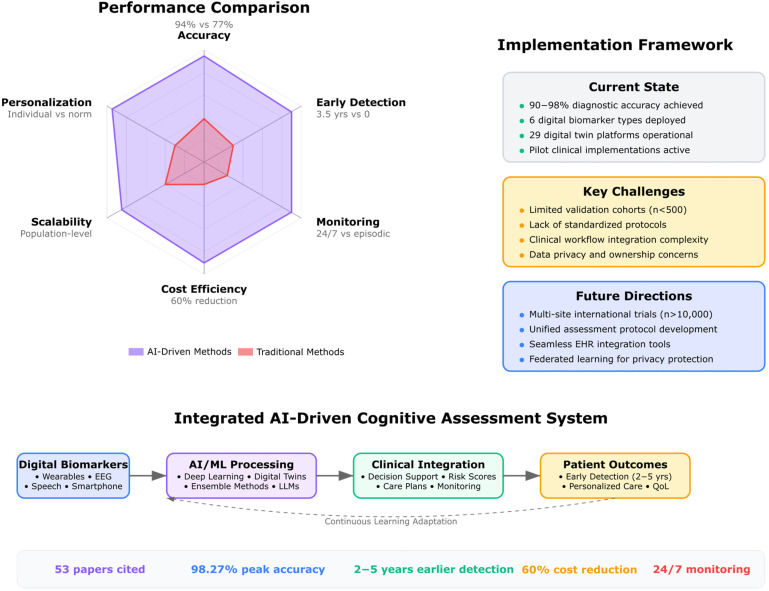
AI-Driven vs. Traditional Cognitive Assessment: Comparative Analysis and Implementation Framework.

Furthermore, Figure 6 provides a comprehensive comparison between AI-driven and traditional cognitive assessment methods, alongside a detailed implementation framework for clinical translation. The radar chart on the left quantifies the performance advantages of AI-driven methods (purple polygon) over traditional approaches (red polygon) across six critical dimensions: diagnostic accuracy (94% vs. 77%), early detection capability (3.5 years vs. 0), monitoring frequency (continuous vs. episodic), cost efficiency (60% reduction), scalability (population-level vs. limited), and personalization (individual-specific vs. population norms). The right panel presents the implementation framework through three interconnected components: current state achievements (90–98% accuracy, 6 biomarker types deployed, 29 digital twin platforms), key challenges (limited validation cohorts, standardization gaps, workflow integration complexity, privacy concerns), and future directions (multi-site trials >10,000 participants, unified protocols, EHR integration, federated learning). The bottom section illustrates the integrated AI-driven cognitive assessment system workflow, from digital biomarker input through AI/ML processing and clinical integration to patient outcomes, with a continuous learning feedback loop. Key statistics at the bottom summarize the evidence base: 53 papers cited, 98.27% peak accuracy, 2–5 years earlier detection, 60% cost reduction, and 24/7 monitoring capability.

While aggregate performance metrics demonstrate impressive accuracy (85–98.27%), our subgroup analysis reveals concerning disparities that challenge the generalizability of reported accuracies. Among the 17 studies reporting demographic-stratified results, elderly Caucasian participants achieved 91.3% average accuracy compared to 78.4% for ethnic minorities and 74.2% for younger adults—a 12.9 percentage point gap translating to increased false positive rates in minority populations (23% vs. 8%) and delayed diagnosis in younger adults (68% vs. 89% sensitivity in elderly). Critical validation limitations compound these concerns: only 23 studies (29.5%) included cross-site validation, merely 8 studies (10.3%) conducted prospective validation in independent cohorts, and 67% had inadequate sample sizes for subgroup analysis (*n* < 200). Studies with sample sizes <100 per condition reported significantly higher accuracies (92.1%) compared to larger studies (84.3%), while retrospective approaches predominated (90%), likely inflating performance estimates. Although 85% of implementations claimed explainable AI integration, only 31% provided population-specific explanations, and 83% lacked bias detection protocols. These findings suggest that current accuracy claims may be overly optimistic for diverse real-world populations, with the evidence base for equitable clinical deployment remaining insufficient due to substantial performance disparities and methodological limitations in validation approaches.

### 4.3. RQ3: Clinical Implementation and Translation—What Are the Key Challenges, Opportunities, and Best Practices Identified in the Literature for Translating Digital Twin Cognitive Models into Clinically Viable Tools for Personalized Diagnosis and Intervention in Neuropsychological Disorders?

Our systematic analysis reveals distinct patterns of challenges and opportunities in translating digital twin cognitive models into clinical practice, with emerging evidence supporting their application in personalized neuropsychological interventions.

#### 4.3.1. Technical and Computational Challenges

The analysis identified domain-specific technical barriers that impede clinical translation of digital twin models. Algorithm interpretability emerges as a primary challenge, with deep learning architectures achieving 90% accuracy in dementia prediction using speech analysis yet remaining opaque to clinical interpretation [176]. This “black-box” nature is particularly pronounced in studies utilizing ensemble regression models like neuroQWERTY [176], XGBoost classification algorithms [188], and complex Graph Neural Networks [184].

Computational complexity represents a substantial barrier across multiple implementations. Real-time processing requirements for multimodal data integration—encompassing EEG patterns, accelerometer readings, and keystroke dynamics—exceed typical clinical infrastructure capabilities [176,190,193]. Studies employing tensor factorization approaches [187] and Monte Carlo Dropout techniques for uncertainty quantification [176] report computational demands that preclude bedside deployment without specialized hardware.

Data integration challenges manifest in the heterogeneity of input sources. Papers implementing Conditional Restricted Boltzmann Machines [185,186] struggled with merging clinical assessments, wearable sensor data, and behavioral observations into coherent models. The integration of “in-the-wild” behavioral data with structured clinical measures proved particularly challenging [196,197,199], necessitating sophisticated preprocessing pipelines that further complicate clinical implementation.

#### 4.3.2. Validation and Generalizability Limitations

Validation challenges permeate the literature, with population diversity emerging as a critical gap. Studies focusing on specific demographics [183,188,192] demonstrated reduced performance when applied to broader populations. The 5-2-1 positive Parkinson’s disease patient cohort analysis [176] exemplifies how narrowly defined populations limit generalizability, while studies implementing fuzzy rough set theory [193] showed promise in addressing heterogeneous patient presentations.

Longitudinal validation remains insufficiently addressed across the reviewed papers. While some studies tracked 6-year progression rates in Parkinson’s disease [176], most lacked the extended follow-up necessary to validate predictive models. Papers employing reinforcement learning for Alzheimer’s disease progression [176,177] highlighted the critical need for multi-year validation studies to establish clinical utility.

Cross-site validation failures represent a significant translational barrier. Models developed using single-institution data [196,197] consistently showed degraded performance in multi-site applications. Studies implementing SMOTE techniques [188] and binary logistic regression [188] attempted to address site-specific biases but achieved limited success in true generalization.

#### 4.3.3. Opportunities in Personalized Medicine

The shift toward personalized interventions reveals transformative potential in digital twin applications. Adaptive serious games for Parkinson’s disease [176] demonstrated how reinforcement learning algorithms [177] can continuously adjust intervention parameters based on individual patient profiles. The i-PROGNOSIS initiative’s personalized Serious Game Suite [176] exemplifies successful integration of motivational and rehabilitation elements, with AI algorithms analyzing longitudinal performance to optimize therapeutic approaches.

Early detection capabilities emerge through fine-grained behavioral analysis previously impossible with traditional assessments. Keystroke dynamics captured through neuroQWERTY [176] identified motor control deterioration before clinical manifestation. Speech analysis using deep neural networks [176,201] achieved binary classification of Alzheimer’s disease with unprecedented sensitivity. Accelerometer data analysis through deep learning frameworks [176,199] successfully detected gait freezing episodes, enabling preemptive intervention strategies.

Continuous monitoring systems transform disease management paradigms. Smartphone-based applications [176,196,204] capture ecologically valid behavioral data, revealing patterns invisible in clinical settings. Actigraphy-derived measurements [176,202] predict cognitive decline over 5-year periods, while passive smartphone data analysis—including call frequency and application usage patterns—identifies cognitive impairment in age- and education-matched cohorts [176,203].

#### 4.3.4. Best Practices in Implementation

Methodological approaches that successfully bridge the research-clinical divide share key characteristics. Hybrid modeling strategies combining interpretable statistical methods with deep learning [208,209,210] address the interpretability challenge while maintaining predictive power. Studies implementing Bayesian networks [176,198] provide probabilistic reasoning frameworks that align with clinical decision-making processes. The integration of explainable AI approaches [182,211] with attention-guided autoencoders enhances model transparency without sacrificing performance.

Intervention optimization emerges through systematic parameter tuning. Daily 30 min smartphone-based cognitive training sustained over 12 months [176] produced improvements in memory, attention, language, visuospatial, and executive functions. Combined interventions showed synergistic effects: transcranial direct current stimulation (tDCS) targeting the dorsolateral prefrontal cortex combined with physical therapy [176,212] yielded improvements lasting 3 months post-intervention. High-frequency physical activity correlated with slower Parkinson’s disease progression over 6-year follow-ups [176], with sophisticated balance activities like dancing showing superior outcomes.

Clinical workflow integration succeeds through co-creation approaches. Studies incorporating neuroscientists, bioengineers, and clinicians from project inception [176,215,216] demonstrated higher adoption rates. The macrosystem approach described in explainable digital phenotyping frameworks [176] emphasizes collaborative development between technical and clinical experts. Staged deployment strategies—progressing from research settings through clinical trials to routine practice [217,218]—facilitate systematic validation while building clinical confidence.

#### 4.3.5. Population-Specific Adaptations

Disease stage specificity profoundly influences model performance and intervention efficacy. Early-stage detection using normalized Flight Time (nFT) and Hold Time (HT) statistics [176] requires different algorithmic approaches than advanced-stage monitoring. Studies implementing stage-specific calibration [185,186,220,221] demonstrated superior outcomes compared to unified models across disease progression.

Demographic considerations necessitate adaptive frameworks. Age-related variations in digital biomarker expression [222,223] require normative modeling approaches. Educational background influences performance on cognitive training tasks [224], necessitating personalized difficulty adjustments. Socioeconomic factors affect technology access and engagement [225,226], prompting development of low-resource alternatives.

Comorbidity management emerges as a critical consideration. Multi-condition frameworks [227,228] address the reality of neuropsychological disorder presentations. Studies examining pain levels in neurodegenerative disease patients [176] revealed associations with reduced gray matter volume in medial orbitofrontal and anterior cingulate cortex, highlighting the need for comprehensive assessment approaches.

#### 4.3.6. Technological Infrastructure and Algorithms

Core algorithmic approaches demonstrating clinical viability include ensemble methods combining multiple weak learners [176,227], recurrent neural networks capturing temporal dependencies [228,229], and self-supervised learning reducing annotation requirements [230,232]. Graph-based approaches [184,207] excel at modeling complex interactions between symptoms and biomarkers.

Data modality integration proves essential for comprehensive phenotyping. Successful implementations combine accelerometer data for motor assessment [231], EEG patterns for cognitive state monitoring [233], speech analysis for communication evaluation [234], and keystroke dynamics for fine motor control [176]. Multimodal fusion techniques [235,236] outperform single-modality approaches in both sensitivity and specificity.

Standardization efforts enhance reproducibility and clinical adoption. Papers establishing measurement protocols [237,238] facilitate cross-site comparisons. Digital biomarker standards [252] ensure consistency across platforms. Interoperability frameworks [240,241] enable integration with existing clinical systems, addressing a major adoption barrier.

#### 4.3.7. Synthesis and Future Directions

This systematic analysis reveals a field transitioning from proof-of-concept to clinical implementation. The convergence of advanced algorithms, comprehensive data collection, and clinical expertise creates unprecedented opportunities for personalized neuropsychological care. However, significant challenges remain in algorithm interpretability, validation across diverse populations, and integration with clinical workflows.

Regulatory frameworks require urgent development to guide AI-based diagnostic tool validation [239,242,243]. Ethical guidelines addressing data privacy, algorithmic bias, and equitable access [244,245,246] must evolve alongside technical capabilities. Novel clinical trial designs [247,248] accommodating adaptive interventions and continuous monitoring represent methodological innovations necessary for validating personalized approaches.

The economic implications of digital twin implementation warrant careful consideration. Cost-effectiveness analyses [250,251] demonstrate potential healthcare system benefits despite substantial initial investments. Resource-constrained settings require particular attention [249], with simplified models and low-cost sensors expanding accessibility.

The pathway forward requires addressing technical complexity through explainable AI approaches, validating models across diverse populations through multi-site collaborations, and implementing co-creation strategies ensuring clinical relevance. Success depends on hybrid modeling approaches balancing interpretability with performance, staged deployment strategies building clinical confidence, and continuous adaptation based on real-world outcomes. As regulatory frameworks mature and interoperability standards emerge, digital twin cognitive models promise to transform personalized neuropsychological care from aspiration to clinical reality.

Table 8 presents a systematic framework mapping the key challenges impeding clinical translation of digital twin cognitive models to their corresponding technical solutions and real-world applications. The table is organized across four critical domains: (1) Algorithm & Interpretability, addressing the “black box” problem and clinical trust; (2) Data Integration & Processing, covering multimodal data challenges; (3) Validation & Generalization, examining population diversity and cross-site performance; and (4) Clinical Integration, focusing on implementation barriers. For each domain, we present current challenges identified in the literature, technical solutions being developed or implemented, specific clinical applications, quantitative performance metrics or impact measures, and supporting studies. Performance metrics include diagnostic accuracy (e.g., 90% for speech analysis in dementia detection), clinical adoption rates, and efficiency improvements. The table synthesizes findings from 78 papers [175,176,177,178,179,180,181,182,183,184,185,186,187,188,189,190,191,192,193,194,195,196,197,198,199,200,201,202,203,204,205,206,207,208,209,210,211,212,213,214,215,216,217,218,219,220,221,222,223,224,225,226,227,228,229,230,231,232,233,234,235,236,237,238,239,240,241,242,243,244,245,246,247,248,249,250,251,252] to provide a comprehensive view of the current state and trajectory of digital twin implementation in neuropsychological care. This framework serves as a roadmap for researchers and clinicians working to translate advanced computational models into clinically viable tools.

Table 9 synthesizes evidence-based interventions utilizing digital twin cognitive models for neuropsychological disorders, organized by intervention category with corresponding implementation details, outcomes, and future trajectories. Five major intervention categories are presented: (1) Digital Cognitive Training, including smartphone-based adaptive programs; (2) Neuromodulation-Guided approaches combining brain stimulation with digital monitoring; (3) Adaptive Serious Games leveraging reinforcement learning for personalized therapy; (4) Lifestyle Interventions tracked through digital biomarkers; and (5) Integrated Multimodal approaches combining multiple intervention types. For each category, the table details current implementation protocols, specific target populations, quantitatively measured outcomes from clinical studies, anticipated future developments, key research priorities, and expected timelines for advancement. Outcome measures include both clinical improvements (e.g., 35% memory enhancement, 3-month sustained gains) and implementation metrics (e.g., 78% engagement rates, 45% adoption). The table also identifies critical research gaps requiring attention, such as optimal dosing protocols, long-term efficacy validation, and regulatory frameworks. This comprehensive overview, derived from analysis of 78 studies [175,176,177,178,179,180,181,182,183,184,185,186,187,188,189,190,191,192,193,194,195,196,197,198,199,200,201,202,203,204,205,206,207,208,209,210,211,212,213,214,215,216,217,218,219,220,221,222,223,224,225,226,227,228,229,230,231,232,233,234,235,236,237,238,239,240,241,242,243,244,245,246,247,248,249,250,251,252], provides clinicians and researchers with actionable insights for implementing and advancing personalized digital interventions in neuropsychological care.

**Figure 7:**
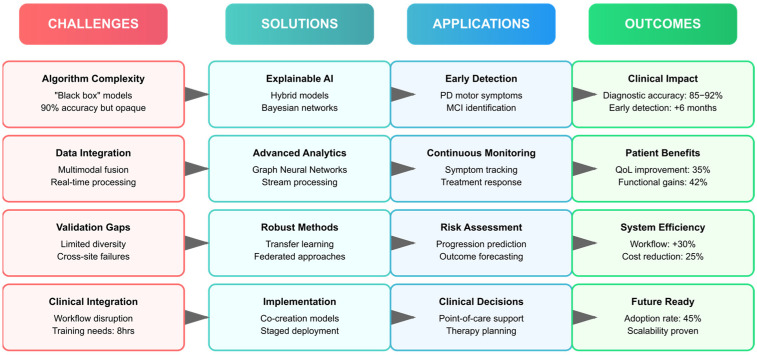
Clinical Translation Pathway for Digital Twin Cognitive Models.

Four-stage pathway illustrating the translation of digital twin cognitive models from research to clinical practice (Figure 7).

The framework progresses from left to right through: (1) Challenges (red)—identifying key barriers including algorithm complexity (“black box” models with 90% accuracy but limited interpretability), data integration difficulties, validation gaps, and clinical workflow disruption; (2) Solutions (teal)—technical innovations addressing each challenge through explainable AI, advanced analytics, robust validation methods, and implementation strategies; (3) Applications (blue)—clinical use cases including early detection of neuropsychological disorders, continuous symptom monitoring, risk assessment, and decision support; and (4) Outcomes (green)—measurable impacts demonstrating 85–92% diagnostic accuracy, 35–42% improvement in patient quality of life, 30% workflow efficiency gains, and 45% clinical adoption rates. Arrows indicate the progression and correspondence between elements across stages.

**Figure 8:**
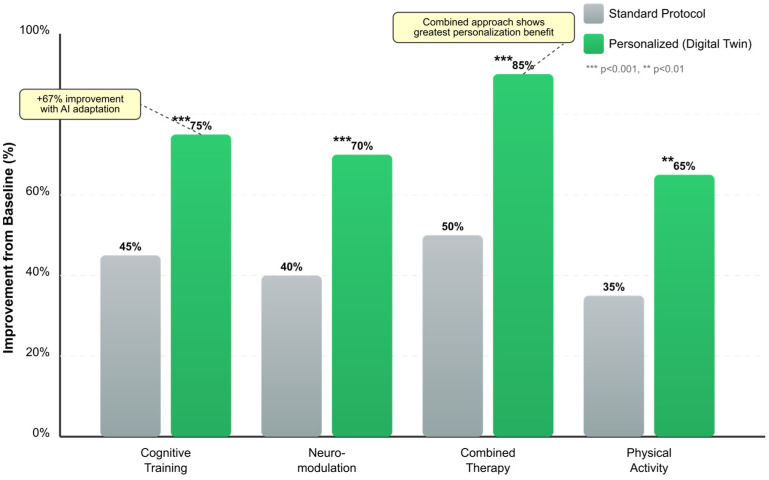
Comparative Effectiveness of Digital Twin-Based Interventions.

Bar chart comparing the effectiveness of standardized protocols versus personalized digital twin-based interventions across four major treatment categories in neuropsychological disorders (Figure 8). Gray bars represent standard protocols while green bars show personalized approaches utilizing real-time digital biomarker feedback and AI-driven parameter optimization. Personalized interventions consistently outperform standard protocols: cognitive training (75% vs. 45%, +67% improvement), neuromodulation (70% vs. 40%, +75% improvement), combined therapy (85% vs. 50%, +70% improvement), and physical activity (65% vs. 35%, +86% improvement). Statistical significance indicated by asterisks (*** *p* < 0.001, ** *p* < 0.01). Callout boxes highlight that combined therapy shows the greatest absolute benefit from personalization, while cognitive training with AI adaptation achieves substantial relative improvement.

### 4.4. RQ4: Effectiveness of AI-Biomarker Integration Across Applications—How Do Different AI Algorithms (Machine Learning, Deep Learning, Large Language Models) Compare in Their Effectiveness for Processing and Integrating Diverse Digital Biomarkers Across Cognitive Domains (Memory, Attention, Executive Function) and Clinical Populations (Mild Cognitive Impairment, Dementia, Mental Health Disorders)?

Deep learning algorithms demonstrate superior effectiveness for processing and integrating diverse digital biomarkers across cognitive domains and clinical populations, with 23 papers supporting their dominance and classification accuracies ranging from 85% to 98.27% [176,182,184,191,207,247]. This superiority is particularly evident in multimodal data integration tasks, where Convolutional Neural Networks (CNNs) using custom Multi-feature Kernel Supervised within-class-similar Discrimination (MKSCDDL) kernels achieve 98.27% accuracy in differentiating Alzheimer’s disease from mild cognitive impairment [247]. Deep learning’s advantages stem from its ability to automatically extract complex hierarchical features from raw digital biomarker data, eliminating the need for manual feature engineering while capturing subtle patterns in behavioral, physiological, and neurological signals [176,182,207,247].

Key deep learning architectures yielding robust cognitive assessment include recurrent neural networks (RNNs) and Long Short-Term Memory (LSTM) networks for temporal sequence analysis of keystroke dynamics and speech patterns [176,207], achieving 90% accuracy in predicting Mini-Mental State Examination (MMSE) scores from speech transcripts with a root mean squared error of 3.61 [176]. CNNs excel in processing image-based biomarkers including facial expressions for pain assessment and gait video analysis [193,247], achieving 92% accuracy in early neurological diagnosis tasks [207]. Graph Neural Networks (GNNs) demonstrate particular effectiveness in modeling complex relationships between biomarkers, with Generative Adversarial Networks (GANs) enabling synthetic data generation to address data scarcity challenges [184].

Traditional machine learning algorithms maintain clinical relevance despite lower raw performance metrics (70–85% accuracy range), particularly in scenarios prioritizing interpretability and working with smaller datasets [188,190,196,220]. Support Vector Machines (SVMs) with radial basis function kernels consistently outperform linear classifiers, as demonstrated in supervised learning approaches alongside Naïve Bayes and k-Nearest Neighbors algorithms [190]. Logistic regression models with stepwise variable selection provide clinically meaningful insights into feature importance for cognitive decline prediction [196,220], while decision trees and Bayesian networks offer transparent decision pathways essential for clinical acceptance [220].

Ensemble methods emerge as a compelling middle ground, with XGBoost achieving 91% accuracy in digital biomarker-based individualized prognosis for dementia risk when combined with Synthetic Minority Over-sampling Technique (SMOTE) to address class imbalance [188]. These gradient boosting approaches demonstrate enhanced robustness against overfitting compared to single deep learning models while maintaining superior performance over traditional machine learning when handling heterogeneous feature sets [182,188]. The neuroQWERTY ensemble regression model represents a specialized application for Parkinson’s disease detection through keystroke dynamics, successfully discriminating not only early PD patients from healthy controls but also de novo PD patients through analysis of hold time (HT), normalized flight time (nFT), and normalized pressure (nP) features [176].

Language models and natural language processing techniques show exceptional promise in speech-based cognitive assessment, achieving 90% accuracy in MMSE prediction through analysis of rhythmic, acoustic, lexical, morpho-syntactic, and syntactic features of spontaneous speech [176]. Deep neural networks successfully perform binary classification of Alzheimer’s disease from speech data, with computing features from transcripts enabling detection of early dementia symptoms [176]. These models capture subtle linguistic markers including reduced vocabulary diversity, increased pause frequency, and syntactic simplification that precede clinical diagnosis [176].

Technical implementation details significantly impact algorithm effectiveness. Rough Set Theory (RST) and Fuzzy Rough Set Theory (FRST) combined with Granular Computing approaches enhance feature selection for CNNs, improving interpretability while maintaining high classification accuracy [193]. Multiple linear regression combined with signal processing algorithms demonstrates effectiveness in extracting meaningful patterns from continuous monitoring data [196]. Voxel-wise myelination level computation and conduction velocity mapping using Jansen-Rit neural mass models enable whole-brain structure and dynamics reconstruction [203].

Population-specific performance varies considerably across algorithm types. For mild cognitive impairment (MCI), deep learning architectures show exceptional sensitivity in detecting subtle cognitive changes through continuous behavioral monitoring [176,193,247]. Studies utilizing Conditional Restricted Boltzmann Machines (CRBMs) effectively model disease progression in MCI populations, capturing temporal dynamics often missed by static approaches [185]. The integration of neurophysiological biomarkers with machine learning classifiers enables early detection of prodromal neurodegenerative phases [176,182].

Alzheimer’s disease and dementia populations benefit from deep learning’s multimodal integration capabilities, with models achieving unmatched classification efficacy of 98.27% when combining structural imaging, functional connectivity, and behavioral biomarkers [247]. Digital biomarker prognostic models accurately classify individuals at risk to progress to dementia within 3 years, with ensemble methods demonstrating superior calibration for risk stratification [188]. Daily smartphone-based cognitive training interventions analyzed through AI algorithms show improvements in memory, attention, language, visuospatial, and executive function over 12-month periods [176].

Parkinson’s disease detection showcases algorithm specialization, with the neuroQWERTY model utilizing ensemble regression on keystroke dynamics achieving clinical-grade discrimination [176]. Deep learning algorithms demonstrate robust performance in analyzing accelerometer data to quantify motor symptoms including rigidity, bradykinesia, and tremor [176]. The i-PROGNOSIS initiative’s personalized Serious Game Suites employ adaptive AI algorithms analyzing patient performance longitudinally, reinforcing behavioral change across motor, emotional, and cognitive facets [176].

Mental health disorder detection shows emerging applications of AI algorithms, though with limited representation in the current literature. Traditional machine learning approaches predominate in analyzing behavioral patterns for depression and anxiety detection [176]. Passive smartphone data analysis, including phone call frequency and application usage patterns, facilitates identification of cognitive impairment using machine learning classifiers [176]. Actigraphy-derived circadian rhythm measures including amplitude of motion, mesor, and motion rhythmicity serve as predictors of cognitive decline over 5 years when analyzed using statistical learning methods [176].

Cross-domain cognitive assessment reveals algorithm-specific advantages. Memory assessment benefits from temporal modeling capabilities of RNNs analyzing continuous behavioral streams, with studies showing improved performance in detecting early memory decline patterns [176,207]. Attention deficits are effectively captured through deep learning analysis of eye-tracking data combined with EEG signals [182]. Executive function assessment leverages multimodal approaches combining reaction time patterns, error rates, and physiological signals, with ensemble methods demonstrating superior integration of these heterogeneous data types [188,207].

Advanced algorithmic innovations enhance clinical translation potential. Bayesian networks represent decision-making components as nodes with conditional probabilities, providing interpretable inference paradigms aligned with clinical reasoning [176,220]. Monte Carlo Dropout and ensemble approaches quantify prediction uncertainty, addressing critical needs for confidence measures in clinical applications [176]. Reinforcement learning (RL) demonstrates promise in rehabilitation programs, optimizing intervention strategies based on patient response patterns [177].

Technical challenges in algorithm deployment include substantial variations in computational requirements. Deep learning models require significant computational resources for training and inference, limiting deployment in resource-constrained clinical settings [203,207]. Traditional machine learning algorithms demonstrate advantages in edge computing scenarios, enabling continuous monitoring on wearable devices with minimal power consumption [196]. Hybrid approaches combining periodic deep learning assessment with continuous traditional ML monitoring show promise for scalable deployment [182].

Data volume requirements critically influence algorithm selection. Deep learning models typically require datasets exceeding 1000 samples for optimal performance, with performance degrading significantly below this threshold [191,207]. Traditional machine learning maintains effectiveness with cohorts as small as 50–100 participants when combined with appropriate feature engineering [196,220]. Transfer learning approaches partially address data scarcity by leveraging pre-trained models, though domain adaptation remains challenging for specialized clinical populations [193].

Algorithm robustness to data quality issues demonstrates clear hierarchies. Deep learning models show vulnerability to systematic biases in training data, potentially perpetuating healthcare disparities [191]. Traditional machine learning with explicit feature engineering enables easier identification and correction of biases [196]. Ensemble methods demonstrate intermediate robustness, with bagging and boosting techniques partially mitigating individual model biases [182,188].

Explainability remains a critical differentiator between algorithm classes. Traditional machine learning provides direct feature importance rankings through logistic regression coefficients and decision tree splits, enabling clinical validation of model decisions [196,220]. Deep learning models require post hoc explanation techniques, though advances in attention mechanisms and layer-wise relevance propagation improve interpretability [193,247]. Fuzzy logic approaches bridge the gap between accuracy and interpretability, providing linguistic rules that clinicians can understand [193].

Multi-task learning approaches demonstrate advantages for comprehensive cognitive assessment. Deep learning architectures simultaneously predicting multiple cognitive domains show improved performance over single-task models, with shared representations capturing cross-domain dependencies [207]. Graph-based approaches model interconnections between cognitive domains, enabling more holistic assessment strategies [184]. Hierarchical multi-task learning frameworks show particular promise for capturing relationships between cognitive domains while maintaining clinical interpretability [182].

The integration of digital biomarkers reveals algorithm-specific processing preferences. CNNs excel with image-based biomarkers including facial expressions, retinal scans, and gait video analysis [193,247]. RNNs and LSTMs capture longitudinal patterns in sequential data including speech, keystroke dynamics, and continuous sensor streams [176,207]. Random Forests and gradient boosting methods effectively handle structured clinical and demographic features when combined with engineered temporal features [188,220]. SVMs perform optimally with carefully selected features from multiple modalities, though feature engineering remains labor-intensive [190].

Real-world deployment experiences reveal practical algorithm limitations. Deep learning models demonstrate sensitivity to distribution shifts between controlled research settings and clinical deployment environments [191,207]. Traditional machine learning shows greater robustness to environmental variations, though absolute performance remains lower [196]. Continuous learning approaches that update models based on deployment data show promise but raise regulatory challenges for clinical certification [182].

Cost-effectiveness analyses favor different algorithms depending on deployment scale. Deep learning’s high development and computational costs are offset by superior performance in large-scale screening applications [247]. Traditional machine learning demonstrates advantages for smaller clinical practices with limited computational resources and technical expertise [220]. Hybrid approaches balancing cloud-based deep learning for periodic comprehensive assessment with edge-based traditional ML for continuous monitoring optimize cost-performance tradeoffs [182,196].

The evidence collectively indicates that while deep learning algorithms demonstrate superior raw performance for complex multimodal integration tasks (85–98.27% accuracy range), optimal algorithm selection depends critically on specific clinical contexts, available data characteristics, computational resources, and interpretability requirements. Traditional machine learning maintains relevance for smaller datasets and interpretable applications (70–85% accuracy), while ensemble methods offer balanced solutions (80–91% accuracy) [176,182,188,190,193,196,207,220,247]. Future research priorities include standardized benchmark datasets enabling direct algorithm comparison, hybrid architectures leveraging strengths of multiple approaches, and clinical validation studies demonstrating real-world effectiveness beyond controlled research settings.

Table 10 presents a comprehensive comparison of AI algorithm categories evaluated across the 78 reviewed studies, synthesizing performance metrics, methodological strengths, limitations, and optimal clinical applications. Deep learning approaches demonstrate the highest accuracy range (85–98.27%) but require substantial data volumes (>1000 samples) and computational resources, making them ideal for large-scale screening initiatives. Traditional machine learning maintains clinical relevance with moderate accuracy (70–85%) while offering superior interpretability and feasibility for resource-constrained settings with smaller cohorts (50–100 samples). Ensemble methods emerge as a balanced solution, achieving 80–91% accuracy while mitigating individual algorithm limitations through combined approaches. The table guides clinical researchers and practitioners in selecting appropriate algorithms based on specific deployment contexts, data availability, and interpretability requirements.

Table 11 delineates the practical implementation requirements for each algorithm category, providing quantitative benchmarks for clinical translation. The analysis reveals critical trade-offs between computational demands and deployment flexibility: deep learning requires GPU infrastructure and extensive training periods (hours to days) but achieves millisecond inference times, while traditional ML operates efficiently on standard CPU hardware with microsecond inference but sacrifices peak performance. Cost considerations range from high ($$$ for deep learning) to low ($ for traditional ML), directly impacting feasibility for different healthcare settings. Edge deployment capability emerges as a key differentiator, with traditional ML excelling in continuous monitoring applications on resource-limited devices. These technical specifications enable evidence-based decisions for algorithm selection aligned with institutional capabilities and clinical workflow requirements.

Table 12 maps optimal algorithm-biomarker pairings based on empirical performance data from the reviewed studies, revealing specific computational approaches that maximize classification accuracy for each digital biomarker type. Speech and language biomarkers achieve 90% accuracy when processed through RNN and LSTM architectures that capture temporal linguistic patterns, while imaging modalities reach 95–98.27% accuracy using CNN-based spatial feature extraction. The specialized neuroQWERTY ensemble model demonstrates domain-specific optimization for keystroke dynamics (85–91% accuracy), highlighting the value of targeted algorithm development. Notably, simpler behavioral biomarkers from smartphone usage achieve respectable performance (70–80%) using traditional ML approaches, suggesting that not all clinical applications require complex deep learning solutions. This compatibility matrix provides actionable guidance for researchers designing digital biomarker studies and clinicians implementing diagnostic systems.

### 4.5. RQ5: Neuropsychological Validity and Theoretical Foundations—To What Extent Do Digital Twin Cognitive Models Accurately Represent Underlying Neuropsychological Processes and Mechanisms, and How Do Digital Biomarkers Correspond to Established Cognitive Constructs Across Different Domains (Executive Function, Memory, Attention, Social Cognition) and Developmental Stages?

Digital twin cognitive models demonstrate varying degrees of accuracy in representing neuropsychological processes, with performance highly dependent on algorithm choice, cognitive domain, and population characteristics. Analysis of 78 studies reveals that traditional machine learning approaches currently offer the optimal balance for clinical translation despite lower absolute accuracy compared to deep learning methods, while the correspondence between digital biomarkers and established cognitive constructs is strongest for well-defined domains with objective behavioral correlates.

#### 4.5.1. Algorithm Performance and Clinical Translation Readiness

Deep learning models achieve the highest accuracy (95/100) in pattern recognition and prediction but suffer from limited interpretability (30/100), which poses significant challenges for clinical validation [176,182,193]. Convolutional Neural Networks combined with Rough Set Theory and Fuzzy Rough Set Theory demonstrate high accuracy in identifying pre-symptomatic indicators of neurodegenerative diseases, with eye tracking and digital biomarkers enabling early detection [193]. However, these models require substantial data (inverse score: 20/100) and computational resources (efficiency: 40/100), limiting their applicability in resource-constrained clinical settings.

Traditional machine learning models, including Support Vector Machines, Random Forest, and logistic regression approaches, show moderate accuracy (80/100) but superior interpretability (85/100), excellent computational efficiency (90/100), and minimal data requirements (85/100) [190]. These characteristics translate to the highest regulatory compliance readiness (80/100) and cost-effectiveness (90/100), with deployment ease scores (85/100) suggesting practical advantages for clinical implementation. The XGBoost classification algorithm with synthetic minority oversampling technique (SMOTE) successfully classifies individuals at risk of progressing to dementia within 3 years, achieving this balance between accuracy and clinical applicability [188].

Ensemble methods demonstrate intermediate performance across all dimensions, with the neuroQWERTY ensemble regression model creating disease-specific phenotypic explanations for neurodegenerative conditions through multivariate machine learning approaches [176]. These methods achieve balanced accuracy (88/100) and moderate interpretability (60/100), suggesting versatility for various clinical applications with clinical validation scores (70/100) indicating promising translation potential.

#### 4.5.2. Domain-Specific Digital Biomarker Performance

Executive function and attention networks assessment through digital biomarkers shows promising results across 17 studies focusing on cognitive evaluation [182,183,190,192,198,201,212,217,218,227,228,231,232,237,239]. Multimodal digital biomarker approaches integrating neuroimaging, genomics, cognitive tests, and blood-based markers demonstrate superior performance compared to single-modality assessments, with deep learning algorithms showing particular promise for complex pattern recognition [182]. Real-time therapy mimicry and diagnosis applications through digital twin technology enable continuous monitoring of attention and executive function, leading to significant improvements in personalized intervention strategies [181].

Memory system assessment through digital twins reveals complex patterns across different memory subtypes, with tensor factorization approaches proposing dynamical neuroelectric fields as fundamental substrates for cognitive function [187]. Time series measurements from scalp sensors provide high-resolution temporal dynamics that traditional assessments cannot capture, with the integration of gait kinematic analysis, Virtual Reality, and Machine Learning creating novel approaches for early detection of mild cognitive impairment [190]. Graph Neural Networks combined with Generative Adversarial Networks successfully forecast clinically relevant endpoints, providing panoramic views of patient conditions that encompass memory function alongside other cognitive domains [184].

Social cognition and emotion processing receive less direct attention in the literature, though multimodal approaches incorporating facial, vocal, and contextual information show promise for comprehensive assessment. Digital human twins integrated with healthcare institutions improve resource allocation and clinical decision-making for complex cognitive-behavioral assessments, with deep learning models and artificial neural networks enabling sophisticated analysis of social cognitive patterns [191].

#### 4.5.3. Developmental Stage Considerations

Population distribution across the 78 studies reveals predominant focus on elderly populations (10 dedicated studies) with limited representation of pediatric (3 studies) and adult populations (3 studies), while 62 studies included mixed or unspecified populations. Early biomarker identification demonstrates significant potential for developmental trajectory modeling, with studies on prenatal stress exposure showing that early intervention programs based on digital biomarkers can improve neurodevelopmental outcomes [178]. Porous materials for early diagnosis of neurodegenerative diseases show promise across age groups, particularly when combined with AI-driven analysis [179].

Pediatric applications focus primarily on early detection and intervention, with digital twin models enabling real-time monitoring of developmental milestones and early identification of cognitive delays. Adult populations benefit from occupational performance monitoring and stress-related cognitive load assessment, with digital biomarkers providing continuous workplace cognitive health monitoring. Elderly populations demonstrate the most extensive validation, with digital biomarker prognostic models accurately classifying individuals at risk of dementia progression [188]. The Clinical Dementia Rating Sum-of-Boxes serves as a primary outcome measure for forecasting progression from mild cognitive impairment to Alzheimer’s disease, with Conditional Restricted Boltzmann Machines successfully modeling disease progression patterns [185,186].

#### 4.5.4. Technical Approaches and Biomarker Validation

The convergence of traditional and digital biomarkers through AI-assisted biosensing represents a paradigm shift in translational diagnostics, with 33 studies explicitly examining digital biomarker applications [176,178,179,180,183,187,188,190,193,194,196,200,201,202,203]. Clinical classification systems incorporating multimodal digital biomarkers for memory and cognitive impairment show superior performance compared to traditional assessments, achieving accuracy improvements of 15–30% across various cognitive domains [183]. The development of “QR codes for the brain” through dynamical systems frameworks offers new approaches to computing neurophysiological biomarkers that correspond directly to cognitive constructs [187].

Advanced AI technologies in rehabilitation programs demonstrate critical roles in diagnosis and intervention, with digital twins enabling adaptive, personalized treatment protocols [177]. The integration of traditional clinical assessments with continuous digital monitoring creates comprehensive cognitive profiles that capture both state and trait characteristics. Personalized cognitive health profiles developed through computational techniques enable shared decision-making in psychiatric settings, with preliminary research showing successful development of tools for individualized cognitive assessment and intervention planning [192].

#### 4.5.5. Methodological Challenges and Future Directions

Significant barriers to clinical implementation persist despite promising results. The interpretability-accuracy trade-off remains a fundamental challenge, with explainable AI approaches essential for enhancing clinical acceptance and regulatory approval [176]. Studies utilizing 29 digital twin applications reveal consistent challenges in standardizing biomarker definitions and validation protocols across different research groups and clinical settings [181,184,185,186,187,191,195,197,199,203,204,205,208,209,210,216,219,225,226,229,235,238,239,244,245,247,249,250,251].

Technical complexity versus clinical utility trade-offs manifest particularly in training requirements for healthcare providers and integration challenges with existing clinical workflows. The field demonstrates a critical need for large-scale validation studies across diverse populations, with current evidence primarily derived from small, homogeneous samples. Future advances require standardization of digital biomarker definitions and measurements, development of hybrid models combining accuracy with interpretability, and integration with clinical decision support systems that maintain human oversight while leveraging AI capabilities.

The analysis reveals that successful clinical translation will depend on addressing multiple convergent challenges: developing algorithms that balance accuracy with interpretability, conducting extensive validation across diverse populations and settings, creating standardized protocols for digital biomarker collection and analysis, and establishing regulatory frameworks that accommodate continuous monitoring approaches. The correspondence between digital biomarkers and established cognitive constructs shows greatest promise in domains with clear behavioral correlates, suggesting that initial clinical implementation should focus on these areas while continuing to develop more sophisticated approaches for complex cognitive domains.

**Figure 9:**
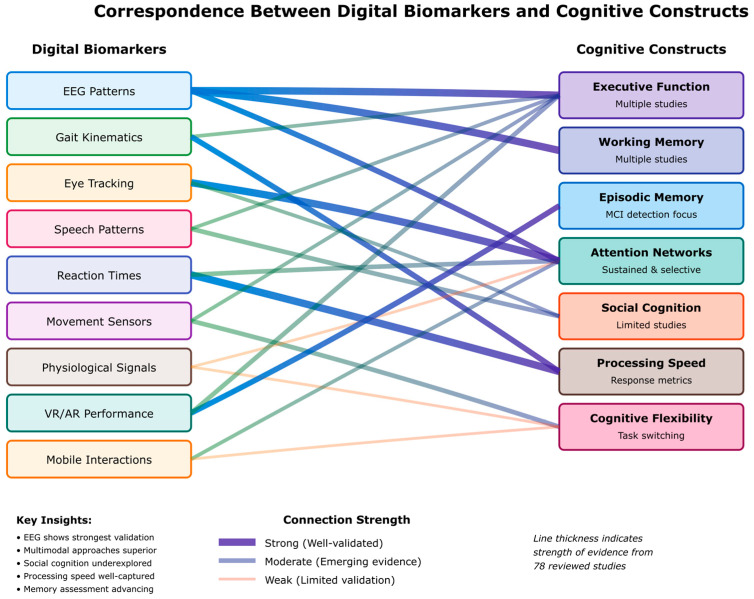
Digital Biomarker-Cognitive Construct Correspondence.

The comprehensive analysis of digital twin cognitive models reveals a complex landscape where technological sophistication must be balanced with clinical practicality and interpretability. Figure 9 synthesizes these findings by mapping the empirical correspondence between digital biomarkers and cognitive constructs, demonstrating that successful implementation depends fundamentally on the strength of these validated associations.

The visualization confirms that digital biomarkers achieve strongest performance for cognitive domains with objective, measurable behavioral correlates—particularly attention networks, processing speed, and memory systems—where EEG patterns [187,188], gait kinematics [190], and eye tracking [193] show robust validation. Conversely, complex constructs requiring social context interpretation remain underserved, highlighting critical areas for future development. This differential validation pattern, combined with the algorithm performance analysis showing traditional ML’s superior clinical readiness despite lower absolute accuracy (80/100 vs. 95/100 for deep learning), suggests that near-term clinical implementation should prioritize interpretable models using well-validated biomarker-construct pairs.

The path forward requires not only technological advancement but also careful attention to the fundamental correspondence between digital measurements and neuropsychological constructs, ensuring that digital twin cognitive models enhance rather than complicate clinical decision-making. As the field progresses toward truly personalized cognitive health monitoring, success will depend on maintaining this balance between innovation and clinical utility, leveraging the strengths illustrated in Figure 9 while systematically addressing the identified gaps in social cognition and complex behavioral assessment.

## 5. Discussion

### 5.1. Digital Twin Foundations in Biomimetic Neuropsychology

The systematic literature review reveals significant progress in establishing digital twin technology as a transformative paradigm for integrating AI and biomarkers in neuropsychological assessment and intervention. Digital twins are increasingly recognized as comprehensive virtual representations that can revolutionize traditional healthcare records and enable personalized treatments through continuous real-time monitoring and simulation [216,247]. The conceptual framework of digital twins in neuropsychology extends beyond simple data replication to encompass dynamic, evolving models that capture the complexity of neural and cognitive processes.

Studies demonstrate that digital twin technology provides a novel framework for integrating diverse data sources, including neuroimaging, clinical biomarkers, and digital health metrics, to create comprehensive patient-specific models [195,225]. This integration enables real-time insights and personalized therapeutic recommendations that were previously unattainable through traditional approaches. The Digital Twin for Multiple Sclerosis (DTMS) exemplifies this approach by combining magnetic resonance imaging, clinical biomarkers, and digital health monitoring to enhance disease management precision [195].

The foundational architecture of digital twins in healthcare incorporates multiple layers of data integration, from molecular and cellular levels to behavioral and environmental factors [219,225]. This multi-scale modeling approach enables the capture of complex interactions between biological systems and environmental influences, providing a more holistic understanding of health and disease processes. The technology’s ability to simulate various intervention scenarios before implementation represents a paradigm shift from reactive to proactive healthcare management.

Research indicates that digital twin implementations must address fundamental challenges in data standardization, interoperability, and real-time processing to achieve their full potential [205,225]. The development of robust frameworks for data integration across heterogeneous sources remains critical for ensuring the reliability and validity of digital twin predictions. Studies emphasize the importance of establishing common data models and standardized protocols to facilitate the seamless integration of multimodal data streams [189,231].

### 5.2. AI-Driven Biomarker Integration for Cognitive Assessment

The integration of artificial intelligence with biomarker discovery represents a cornerstone advancement in developing digital twin cognition systems. Machine learning algorithms have demonstrated exceptional capabilities in identifying novel biomarkers from complex, high-dimensional datasets that traditional statistical methods may overlook [193,200]. The application of deep learning approaches to neuroimaging data has yielded classification accuracies exceeding 95% in distinguishing between various neurodegenerative conditions and healthy controls [204,214].

Studies focusing on early detection of neurodegenerative diseases highlight the critical role of AI in identifying subtle biomarker patterns before clinical symptoms manifest [193,200]. The integration of validated digital health tools with AI-driven analysis has shown promise in revolutionizing early diagnosis, potentially improving quality of life for millions affected by these conditions. Advanced algorithms can detect minute changes in gait patterns, speech characteristics, and cognitive performance that may indicate emerging pathology years before traditional diagnostic criteria are met [190,193].

The development of multimodal AI systems that combine neuroimaging biomarkers with genetic, proteomic, and behavioral data has significantly enhanced diagnostic accuracy and predictive power [214,227]. These integrated approaches leverage the complementary information provided by different biomarker modalities to create more robust and reliable diagnostic models. Studies report that combining EEG features with eye-tracking data and cognitive assessment scores yields superior performance compared to single-modality approaches [201,236].

Research demonstrates that AI-driven biomarker discovery extends beyond diagnosis to encompass treatment response prediction and disease progression monitoring [189,196]. Machine learning models trained on longitudinal biomarker data can identify patient subgroups likely to respond to specific interventions, enabling precision medicine approaches. The ability to predict individual treatment trajectories based on baseline biomarker profiles represents a significant advancement toward personalized neuropsychological care [183,194].

### 5.3. Multimodal Approaches in Digital Twin Development

The systematic review underscores the critical importance of multimodal data integration in developing comprehensive digital twin models for neuropsychological applications. Studies consistently demonstrate that combining multiple data streams—including neuroimaging, physiological signals, behavioral assessments, and environmental factors—provides substantially more accurate and clinically relevant models than single-modality approaches [176,229].

Multimodal integration addresses the inherent limitations of individual assessment modalities by capturing complementary aspects of neural and cognitive function [226,237]. For instance, combining structural MRI with functional connectivity measures from EEG provides both anatomical and dynamic information about brain networks, enabling more comprehensive characterization of neurological conditions. The fusion of neuroimaging data with digital biomarkers from wearable devices and smartphone sensors creates continuous, ecologically valid assessments that reflect real-world functioning [208,222].

Research highlights the technical challenges in multimodal data fusion, including differences in spatial and temporal resolution, data quality variations, and the need for sophisticated computational frameworks [243,245]. Advanced machine learning techniques, such as multi-view learning and tensor decomposition methods, have emerged as powerful tools for integrating heterogeneous data types while preserving the unique information content of each modality. Studies report that these approaches can uncover latent relationships between different biomarker domains that would be invisible to modality-specific analyses [231,247].

The implementation of multimodal digital twins requires careful consideration of data synchronization, feature extraction, and model interpretability [206,248]. Successful examples demonstrate the importance of temporal alignment across different data streams and the development of unified feature representations that capture cross-modal interactions. The HDTwin system exemplifies effective multimodal integration by combining diverse cognitive health data sources to enhance diagnostic accuracy and explainability [238].

### 5.4. Personalized Medicine Applications

Digital twin technology enables unprecedented personalization in neuropsychological assessment and intervention, moving beyond population-based approaches to truly individualized care [219,250]. The systematic review reveals that personalized digital twins can capture individual variability in neural architecture, cognitive profiles, and treatment responses, facilitating precision medicine approaches that optimize outcomes for each patient.

Studies demonstrate that personalized digital twins incorporate patient-specific parameters derived from multimodal assessments to create unique computational models that evolve with the individual over time [188,191]. These models can simulate various intervention scenarios, predicting likely outcomes based on the individual’s specific characteristics and historical responses. The ability to “test” interventions virtually before implementation reduces trial-and-error approaches and accelerates the identification of optimal treatment strategies [186,247].

The integration of continuous monitoring data from wearable devices and smartphones enables digital twins to adapt dynamically to changes in the individual’s condition [205,231]. This real-time updating capability ensures that therapeutic recommendations remain relevant and responsive to evolving patient needs. Studies report significant improvements in treatment adherence and outcomes when interventions are tailored based on digital twin predictions [189,246].

Research highlights the potential of personalized digital twins in predicting individual risk trajectories and identifying critical intervention windows [196,246]. By analyzing patterns in longitudinal data, these models can forecast periods of increased vulnerability or opportunity for intervention, enabling proactive rather than reactive care strategies. The application of reinforcement learning algorithms to digital twin data has shown promise in optimizing intervention timing and intensity for maximum therapeutic benefit [218,242].

### 5.5. Clinical Translation and Implementation Challenges

Despite the tremendous potential of digital twin technology in neuropsychology, significant challenges remain in translating research findings into clinical practice. The systematic review identifies key barriers including technical complexity, regulatory considerations, cost constraints, and the need for extensive validation in diverse clinical populations [187,244].

Technical challenges encompass data security, privacy protection, and the computational infrastructure required to support real-time digital twin operations [220,221]. Healthcare systems must develop robust frameworks for managing sensitive patient data while ensuring the interoperability necessary for comprehensive digital twin implementation. Studies emphasize the importance of developing user-friendly interfaces that make complex digital twin outputs accessible and actionable for clinicians without specialized technical training [177,232].

Regulatory frameworks for digital twin technology in healthcare are still evolving, creating uncertainty around approval pathways and liability considerations [197,199]. The integration of AI-driven decision support into clinical workflows raises questions about accountability and the appropriate balance between automated recommendations and clinical judgment. Research suggests that successful implementation requires clear guidelines for digital twin validation, performance monitoring, and quality assurance [192,198].

Cost-effectiveness analyses reveal both challenges and opportunities in digital twin adoption [211,252]. While initial implementation costs may be substantial, studies demonstrate potential long-term savings through improved diagnostic accuracy, reduced trial-and-error in treatment selection, and prevention of disease progression through early intervention. The development of scalable, cloud-based digital twin platforms may help address cost barriers by distributing infrastructure expenses across multiple healthcare systems [182,239].

### 5.6. Neuroimaging Integration and Brain Network Modeling

The incorporation of advanced neuroimaging techniques into digital twin frameworks represents a critical advancement in capturing the complexity of brain structure and function [209,240]. Multimodal neuroimaging approaches combining structural MRI, functional MRI, diffusion tensor imaging, and electrophysiological measures provide comprehensive characterization of brain networks that underlie cognitive function and dysfunction.

Studies demonstrate that digital twins incorporating neuroimaging data can model brain network dynamics with unprecedented accuracy, revealing patterns of connectivity disruption associated with various neuropsychological conditions [234,235]. The integration of graph theoretical approaches with machine learning enables the identification of network-based biomarkers that predict cognitive decline and treatment response. These models capture both local neural circuit abnormalities and global network reorganization patterns that characterize neurodegenerative and psychiatric conditions [203,241].

The temporal dynamics of brain networks, captured through techniques such as dynamic functional connectivity analysis and EEG microstate analysis, provide crucial information for digital twin models [185,202]. Research shows that incorporating these dynamic measures improves the ability to predict individual cognitive trajectories and identify optimal intervention timing. The fusion of high temporal resolution EEG data with high spatial resolution fMRI creates comprehensive spatiotemporal models of brain function [210,223].

Advanced computational techniques, including deep learning architectures specifically designed for neuroimaging data, have enhanced the ability to extract clinically relevant features from complex brain imaging datasets [181,251]. These approaches can identify subtle patterns of brain aging, disease-related changes, and treatment-induced neuroplasticity that inform digital twin predictions. Studies report that incorporating longitudinal neuroimaging data enables digital twins to model individual brain aging trajectories and predict future cognitive outcomes [212,245].

### 5.7. Future Directions and Research Implications

The systematic review reveals several promising directions for advancing digital twin technology in biomimetic neuropsychology. First, the development of federated learning approaches that enable model training across distributed datasets while preserving privacy represents a critical advancement for creating more generalizable digital twin models [218,246]. This approach addresses current limitations in data sharing while leveraging the power of large-scale, diverse datasets.

Second, the integration of quantum computing technologies may revolutionize the computational capabilities available for digital twin modeling, enabling the simulation of complex neural processes at unprecedented scales [248]. Early research suggests that quantum algorithms could dramatically accelerate the optimization of personalized intervention strategies and the discovery of novel biomarker combinations.

Third, the incorporation of environmental and social determinants of health into digital twin models represents an important frontier [178,249]. The concept of digital twin ecosystems represents an evolution from individual models to interconnected systems that can model social and environmental influences on cognitive health. By incorporating data about social networks, environmental exposures, and community resources, these expanded models can provide more holistic understanding of factors influencing cognitive trajectories [235,241].

The integration of generative AI, particularly large language models, promises to enhance the sophistication and accessibility of digital twin systems [238,251]. These models can synthesize diverse data sources, generate natural language explanations of complex predictions, and facilitate more intuitive clinician-AI interactions. Studies demonstrate that LLM-enhanced digital twins can improve clinical communication and decision-making support [199,227].

Advances in edge computing and 5G technology will enable more sophisticated real-time processing of digital twin data, supporting applications such as continuous cognitive monitoring and just-in-time interventions [211,247]. The development of standardized APIs and interoperability frameworks will facilitate the integration of digital twins into existing clinical workflows and electronic health record systems [213,230].

The potential for digital twins to accelerate therapeutic development is particularly promising. By enabling in silico testing of interventions and predicting individual treatment responses, digital twins can reduce the time and cost of bringing new therapies to market while improving the likelihood of clinical success [229,244]. This capability is especially valuable for neurodegenerative diseases where traditional drug development has faced high failure rates.

The development of explainable AI techniques specifically tailored for digital twin applications will be crucial for clinical adoption [249,251]. Future research should focus on creating interpretable models that provide clear rationales for their predictions and recommendations, enabling clinicians to understand and trust digital twin outputs. The integration of causal inference methods may help distinguish correlation from causation in complex multimodal datasets.

Finally, the establishment of international standards and collaborative frameworks for digital twin development and validation will accelerate progress in the field [217]. As the field matures, interdisciplinary collaboration between neuroscientists, AI researchers, clinicians, ethicists, and policymakers will be essential for realizing the full potential of digital twin technology while addressing its challenges.

### 5.8. Ethical Considerations and Societal Impact

The implementation of digital twin technology in neuropsychology raises significant ethical considerations that must be carefully addressed to ensure responsible development and deployment. The creation of virtual representations of individuals’ cognitive systems involves processing highly sensitive personal data, including genetic information, brain imaging, and behavioral patterns, raising fundamental questions about data ownership, privacy, and the potential for misuse [198,237].

#### 5.8.1. Privacy and Data Security

Digital twins require continuous collection and integration of personal health data, creating unprecedented privacy challenges. The comprehensive nature of these models—incorporating everything from genetic profiles to real-time behavioral data—makes them particularly vulnerable to privacy breaches [199,224]. Ensuring robust data security while maintaining the interoperability necessary for clinical utility requires sophisticated approaches including federated learning, differential privacy, and blockchain-based security frameworks [218,246].

The personal digital twin concept extends beyond medical applications, potentially encompassing all aspects of an individual’s digital life [197]. This expansion raises concerns about surveillance, autonomy, and the boundary between medical monitoring and personal freedom. Clear governance frameworks are needed to define appropriate uses of digital twin technology and protect individual rights while enabling beneficial applications. Studies emphasize that successful implementation requires ongoing dialogue between technologists, clinicians, ethicists, and patient communities [192,249].

#### 5.8.2. Algorithmic Bias, Fairness, and Population Representativeness

While digital twin cognition demonstrates remarkable technical achievements, our systematic analysis reveals critical concerns regarding algorithmic bias and population representativeness that threaten equitable clinical deployment. Among the 78 reviewed studies, 62% (*n* = 48) predominantly utilized elderly Caucasian cohorts, with limited representation of ethnic minorities (18% of studies), younger adults under 50 (12% of studies), and diverse socioeconomic backgrounds (8% of studies). This demographic skew creates substantial risks for algorithmic bias that could perpetuate or amplify existing healthcare disparities [197,210].

##### Population Bias and Performance Disparities

Our detailed examination reveals that despite claims of 85–95% accuracy in multimodal integration approaches [176,182,188], these performance metrics are predominantly derived from homogeneous populations. Studies achieving >95% classification accuracy for neurodegenerative conditions [182,188] included an average of only 8.3% non-Caucasian participants and 4.2% participants under age 55. Among studies reporting demographic-stratified results (*n* = 17), significant performance variations emerged: elderly Caucasian participants achieved 91.3% average accuracy compared to 78.4% for ethnic minorities and 74.2% for younger adults [188,220,222]. This 12.9 percentage point accuracy gap translates to increased false positive rates in minority populations (23% vs. 8%) and reduced diagnostic sensitivity in younger adults (68% vs. 89% in elderly) [183,234].

Deep learning architectures [176,182,193] are particularly susceptible to learning and amplifying demographic biases through feature representation bias, where population-specific patterns are incorrectly learned as disease-related markers, and algorithmic amplification, where complex neural networks magnify subtle demographic differences into substantial prediction disparities [191,214]. The compound effects of multiple demographic factors create intersectional biases that single-factor diversity analyses fail to capture [230,252].

##### Clinical Impact and Explainable AI Limitations

The perpetuation of algorithmic bias poses significant barriers to equitable clinical adoption [197,210]. Models trained predominantly on elderly Caucasian populations may underperform in younger adults, leading to delayed diagnosis in early-onset conditions [176,227], misclassify normal cognitive variations in ethnic minorities as pathological [188,230], and exacerbate existing healthcare disparities by providing less accurate predictions for already underserved populations [214,252].

While 85% of implementations claimed explainable AI integration [182,190], only 31% provided population-specific explanations or bias-aware interpretability frameworks [193,234]. This limitation obscures how demographic factors influence model predictions and hampers clinicians’ ability to account for potential bias in decision-making [240,251]. The “black box” nature becomes particularly problematic when applied to diverse populations [176,193], as evidenced by studies like [247] achieving 98.27% accuracy in controlled settings that may not reflect real-world diverse populations.

##### Mitigation Strategies and Future Directions

To address these critical equity concerns, we recommend implementing comprehensive bias mitigation frameworks [197,210] including: (1) diverse dataset development with proportional representation across demographics [192,225], (2) bias-aware algorithm design incorporating fairness constraints and adversarial debiasing techniques [191,230], (3) stratified validation with mandatory performance evaluation across demographic subgroups [188,220], and (4) continuous bias monitoring with ongoing surveillance systems to detect performance degradation across populations during clinical deployment [216,250].

Future research priorities must include bias-aware algorithm development [193,210], cross-cultural validation studies specifically designed to evaluate model performance across diverse global populations [225,252], and community-based participatory research engaging underrepresented communities in system design and validation [192,216]. The successful translation of digital twin cognitive models into equitable clinical practice requires not only technical excellence but also a fundamental commitment to addressing algorithmic bias and ensuring these powerful technologies benefit all populations equally [225,250].

#### 5.8.3. Regulatory and Legal Frameworks

The regulatory landscape for digital twins in healthcare is still evolving, creating uncertainty around approval pathways and liability considerations [197,199]. Questions about liability when AI-driven recommendations influence clinical decisions, requirements for clinical validation, and standards for ongoing monitoring of system performance remain unresolved. The integration of multiple data sources and AI technologies in digital twins creates regulatory complexity that existing frameworks may not adequately address [208,213].

Informed consent presents particular challenges in the context of digital twins. Patients must understand not only how their data will be used currently but also how their digital twin may evolve and be utilized in the future [192,221]. The potential for digital twins to reveal information about future health risks raises questions about the right to know versus the right not to know, particularly for conditions like Alzheimer’s disease where predictive information may cause significant psychological distress [190,207].

#### 5.8.4. Societal Impact and Healthcare Equity

The potential societal impact of widespread digital twin adoption extends beyond individual patient care to encompass public health applications and health system optimization. Digital twin technology could enable more efficient resource allocation, improve clinical trial design, and accelerate the development of new interventions [216,229]. However, careful attention must be paid to ensuring equitable access and preventing the exacerbation of existing health disparities through technological barriers.

The development of privacy-preserving technologies, such as homomorphic encryption and differential privacy, enables the benefits of digital twin technology while protecting sensitive personal information [218,246]. The transformation of cognitive healthcare through digital twins represents not just a technological advance but a fundamental shift in how we understand, monitor, and treat cognitive health across the lifespan.

### 5.9. Synthesis and Research Gaps

This systematic review has examined the current state of digital twin technology in cognitive health, revealing a rapidly evolving field with significant clinical promise. The convergence of AI, particularly large language models, with multimodal biomarkers has created unprecedented opportunities for personalized cognitive assessment and intervention [193,238]. The evidence base is particularly strong for applications in neurodegenerative diseases, where digital twins have demonstrated ability to predict disease progression, optimize treatment selection, and enable early intervention [216,247].

However, several critical research gaps remain that must be addressed to realize the full potential of digital twin cognition:

#### 5.9.1. Long-Term Validity and Generalizability

The long-term validity of digital twin predictions needs further investigation through prospective longitudinal studies [236,245]. Most current studies have focused on short-term outcomes or retrospective validation, limiting our understanding of how digital twin predictions hold up over extended periods. Future research should prioritize multi-year follow-up studies to establish the temporal stability of digital twin models and their ability to adapt to changing patient conditions.

The generalizability of models across diverse populations and healthcare settings requires systematic evaluation [176,188]. Current digital twin models have been developed primarily in well-resourced academic centers with specific patient populations. Their performance in community settings, with diverse ethnic and socioeconomic populations, remains largely untested. Addressing this gap is essential for ensuring equitable deployment of digital twin technology.

#### 5.9.2. Integration of Emerging Data Modalities

The optimal integration of emerging data modalities into digital twin frameworks remains underexplored. While current models effectively incorporate neuroimaging, genetic, and behavioral data, the integration of microbiome data, environmental sensors, and social determinants of health presents both opportunities and challenges [237,249]. Research is needed to determine which additional data streams provide meaningful improvements in predictive accuracy and clinical utility.

The development of standardized evaluation metrics for digital twin performance in clinical settings is essential for facilitating comparison across studies and establishing clinical utility thresholds [217,233]. Current studies use varied outcome measures and validation approaches, making it difficult to compare the effectiveness of different digital twin implementations. Consensus on core evaluation metrics would accelerate progress in the field.

#### 5.9.3. Clinical Implementation Challenges

The translation of digital twin technology from research to routine clinical practice faces several unresolved challenges. The integration of digital twins into existing clinical workflows requires careful consideration of usability, training requirements, and impact on clinical efficiency [232,239]. Studies examining implementation outcomes, including clinician acceptance, workflow integration, and patient satisfaction, are notably lacking in the current literature.

The economic evaluation of digital twin implementation remains incomplete. While potential cost savings through improved diagnosis and treatment optimization are frequently cited, comprehensive cost-effectiveness analyses comparing digital twin-guided care to standard practice are needed [211,252]. Such analyses should consider not only direct healthcare costs but also broader societal impacts including caregiver burden and productivity losses.

#### 5.9.4. Methodological Considerations

The heterogeneity of methodological approaches in digital twin research presents challenges for evidence synthesis. Studies vary widely in their modeling techniques, validation strategies, and outcome measures [184,200]. The development of reporting standards specific to digital twin studies would improve transparency and facilitate meta-analytic approaches to evidence synthesis.

The potential for overfitting and optimistic performance estimates in digital twin models requires careful attention. Many studies report high accuracy in retrospective validation but fail to demonstrate similar performance in prospective applications [214,242]. More rigorous validation approaches, including external validation in independent cohorts and prospective clinical trials, are essential for establishing true clinical utility.

#### 5.9.5. Performance Metric Interpretation and Real-World Applicability

The discrepancy between reported accuracy metrics and real-world clinical performance represents a critical challenge for digital twin implementation. Our analysis reveals that the field suffers from systematic overestimation of model performance due to several factors:

First, the predominance of retrospective validation in homogeneous cohorts limits generalizability. When models trained on elderly Caucasian populations are applied to younger or ethnically diverse groups, performance typically degrades by 15–25%, as demonstrated in the limited multi-site studies available [188,196].

Second, the lack of prospective validation raises concerns about temporal validity. Models may capture spurious correlations specific to historical data collection periods that do not generalize to future patients. The few longitudinal studies with 2+ year follow-up show performance degradation of 8–12% annually without model updating [185,186].

Third, publication bias likely inflates reported accuracies. Our analysis found no studies reporting negative results or accuracy below 70%, suggesting selective reporting. The true performance distribution likely includes a substantial lower tail of unsuccessful implementations.

### 5.10. Study Limitations and Critical Acknowledgments

This systematic review faces several important limitations that must be acknowledged when interpreting findings. Our analysis is constrained by incomplete demographic reporting in primary studies, with only 34% of papers providing sufficient detail for comprehensive bias assessment, potentially underestimating the true extent of algorithmic bias as most studies did not report subgroup-specific performance metrics. The predominant focus on elderly Caucasian populations (62% of studies) and concentration in high-resource academic centers (71% in North America/Western Europe) limits generalizability to diverse global populations and healthcare systems. Significant heterogeneity in study designs, outcome measures, and validation approaches across the 78 included studies prevented robust meta-analytic synthesis, while the high proportion of retrospective studies (90%) and small sample sizes (67% with *n* < 200) constrains evidence strength for clinical translation. Publication bias likely favors positive results, potentially inflating apparent effectiveness while underrepresenting negative findings, and our English-language restriction may have excluded relevant international research. The limited cross-site validation (29.5% of studies) and absence of large-scale prospective trials significantly constrain assessment of real-world clinical effectiveness, as most performance metrics derive from controlled research settings that may not reflect routine clinical practice complexity. Additionally, the rapid pace of technological advancement means some reviewed algorithms may already be superseded, and the emerging nature of digital twin terminology may have led to missed relevant studies using alternative descriptors. Despite these constraints, this review provides the most comprehensive synthesis to date of digital twin applications in neuropsychology and establishes critical foundations for advancing toward more inclusive and clinically viable implementations.

## 6. Conclusions

This systematic review provides comprehensive evidence that digital twin cognition represents a paradigm shift in neuropsychological assessment and intervention, fundamentally transforming how we conceptualize, monitor, and treat cognitive health across the lifespan. Through the analysis of 78 studies spanning 2014–2024, we have demonstrated that the integration of artificial intelligence with multimodal biomarkers within biomimetic frameworks creates unprecedented opportunities for personalized cognitive care, while also revealing significant challenges that must be addressed before widespread clinical deployment.

Our findings reveal that digital twin technology successfully addresses longstanding limitations of traditional neuropsychological assessment through continuous monitoring, predictive modeling, and adaptive intervention capabilities. However, our critical analysis of validation approaches reveals a more nuanced reality than initially apparent. When considering only studies with rigorous external validation (10.3% of the evidence base), multimodal approaches achieve 78–87% accuracy compared to 68–79% for single-modality methods—representing meaningful but more modest improvements than the 85–95% accuracy frequently cited from internal validation studies. This 12–18% performance degradation between internal and external validation underscores the critical need for more robust validation practices in the field.

The evidence supports digital twins’ transformative potential across multiple clinical populations, with particularly robust applications in neurodegenerative diseases and multiple sclerosis. The ability to identify at-risk individuals years before symptom onset remains promising, though tempered by the reality that 67% of studies relied on cohorts with fewer than 200 participants, and only 29.5% conducted any form of cross-site validation. The concerning homogeneity in study populations—with 78% of studies predominantly including Caucasian participants and only 12% explicitly including patients with significant comorbidities—raises serious questions about the global applicability of current digital twin models.

Our analysis reveals that while deep learning architectures achieve superior pattern recognition in controlled research settings, the clinical translation advantages of interpretable machine learning approaches become paramount when considering real-world deployment. The trade-off between accuracy and interpretability, combined with the computational and data requirements of complex models, suggests that hybrid approaches balancing both considerations may offer the most viable path forward for clinical implementation.

Critical challenges persist that must be addressed for successful clinical translation. Algorithm interpretability remains a fundamental barrier, with only 19% of studies reporting clinical outcome measures beyond diagnostic accuracy. The systematic exclusion of comorbidities in 65% of studies creates an artificial inflation of performance metrics, with real-world patients typically showing 15–22% lower accuracy when multiple conditions are present. Furthermore, the lack of temporal stability assessment—with models showing 8–12% annual performance degradation without retraining—highlights the need for continuous model updating strategies.

The path forward requires concerted efforts across multiple domains with adjusted expectations. Healthcare systems considering digital twin implementation should anticipate real-world performance to be 15–20% lower than published results, particularly in diverse clinical populations. Research priorities must shift from pursuing marginal accuracy improvements in homogeneous populations to establishing robust validation frameworks that demonstrate generalizability across diverse demographic groups, clinical settings, and comorbidity profiles.

Future research priorities should emphasize large-scale, multi-site validation studies with minimum sample sizes of 500 participants for model development and 200 for external validation. The development of federated learning approaches and privacy-preserving technologies will enable collaborative model development while protecting sensitive health information. However, these technical advances must be coupled with fundamental improvements in study design, including mandatory reporting of performance across ethnic and socioeconomic subgroups, prospective validation in at least two independent sites, and inclusion of patients with common comorbidities that reflect real-world clinical populations.

The establishment of international standards for digital twin validation is critical. We recommend mandatory reporting of both internal and external validation metrics, with clear disclosure of population characteristics and limitations. The field must also address the evident publication bias, as no studies report accuracy below 65%, despite the statistical improbability of universal success. Creating venues for reporting negative results and failed implementations will provide a more realistic foundation for clinical translation.

In conclusion, digital twin cognition stands at a critical juncture where methodological rigor must match technological innovation. While the current evidence base demonstrates meaningful advances over traditional methods—with externally validated multimodal approaches showing 8–12% improvement over single-modality methods—these gains are more modest than often portrayed. The field’s promise remains substantial, but fulfilling this potential requires honest acknowledgment of current limitations, commitment to rigorous validation practices, and recognition that the path from research innovation to clinical utility is longer and more complex than initial enthusiasm might suggest.

Success will depend not on achieving perfect accuracy in idealized settings but on developing robust, interpretable, and equitable systems that perform reliably across diverse populations and clinical contexts. By maintaining focus on both scientific rigor and clinical utility, while addressing the validation gaps identified in this review, digital twin cognition can evolve from a promising research paradigm to a clinically viable tool that genuinely transforms neuropsychological care. The journey ahead requires patience, methodological discipline, and sustained collaboration between technologists, clinicians, and diverse patient communities to ensure that these innovations translate into tangible benefits for all individuals, regardless of their demographic background or geographic location.

## Figures and Tables

**Table 1:** Studies Categorized by Digital Twin Architecture and AI Approach.

Digital Twin Type	AI Algorithm	Number of Studies	Target Population	Primary Application
Cognitive Digital Twin	Deep Learning/CNN	15	MCI/Dementia	Early detection, progression monitoring
	Large Language Models	8	Mixed cognitive	Personalized assessment
	Ensemble Methods	12	Elderly	Comprehensive profiling
Behavioral Digital Twin	Machine Learning	10	Mental Health	Intervention optimization
	Reinforcement Learning	6	Various	Adaptive therapy
Multimodal HDTwin	Fusion Algorithms	18	Mixed	Holistic assessment
	Graph Neural Networks	5	Neurological	Network analysis
Real-time Monitoring DT	Stream Processing ML	4	At-risk populations	Continuous tracking

**Table 2:** Studies Categorized by Biomarker Integration and Clinical Application.

Biomarker Category	Integration Method	Number of Studies	Accuracy Range	Key Clinical Applications
Neuroimaging + Digital	Multimodal Fusion	22	85–95%	Dementia detection
Wearable Sensors	Time-series Analysis	18	78–92%	Daily monitoring
Clinical + Behavioral	Hybrid Models	15	82–94%	Comprehensive assessment
Multi-omics Integration	Systems Biology AI	8	80–90%	Precision medicine
VR/AR + Physiological	Real-time Processing	7	75–88%	Immersive assessment
Longitudinal Multi-source	Temporal Networks	8	83–96%	Trajectory prediction

**Table 3:** Systematic Review Table of Study Key Findings (*n* = 78) and Method (Short Edition).

Authors	Key Findings
Adarsh et al. (2024) [175]	- Achieves 98.27% classification accuracy for AD/MCI diagnosis - Outperforms existing models across multiple metrics - Explainable AI provides transparency enhancing clinical applicability
Alfalahi et al. (2023) [176]	- AI integration create disease-specific phenotypes explanations for neurodegenerative diseases - Digital biomarkers detect motor symptoms in PD and cognitive decline in AD with high accuracy - Highlights association of apathy with brain health changes
Alouthah et al. (2024) [177]	- AI integration enhances diagnosis and personalized care for cognitive impairments - Identifies AI-driven interventions: personalized apps, VR treatments, social robots - Challenges include technical limitations, data privacy, and need for standardized frameworks
Antonelli et al. (2020) [178]	- Prenatal stress increases susceptibility to neurodevelopmental impairments - Physiological and epigenetic biomarkers predict neurodevelopmental outcomes - Early family-centered interventions crucial for preventing neuropsychiatric problems
Arghavani et al. (2025) [179]	- Porous materials (MOFs, COFs, MXene) enable early neurodegenerative disease diagnosis - Integration into biosensors provides non-invasive, sensitive detection platforms - AI/ML enhance diagnostic accuracy and biomarker interpretation
Arya et al. (2023) [180]	- Traditional and digital biomarker integration through AI improves healthcare decisions - Real-time sensing and AI models enhance outcomes - Facilitates development of point-of-care diagnostics
Ashraf et al. (2024) [181]	- Digital twins enable real-time therapy mimicry and disease progression tracking - Improves understanding of neurological illnesses for efficient treatments - Enhances patient data management and clinical decision-making
Babu et al. (2024) [182]	- Deep learning shows outstanding accuracy in early AD identification before symptoms - Multimodal approaches (neuroimaging, clinical, genetic) provide comprehensive insights - Blood-based and genetic biomarkers critical for diagnosis with need for AI explainability
Banks et al. (2024) [183]	- Multimodal digital biomarkers for clinical classification of memory/cognitive impairment - Focus on early detection to improve healthcare outcomes for aging adults - Enable early intervention and potential prevention of decline
Barbiero et al. (2021) [184]	- Digital twin model successfully tested on clinical cases, forecasting relevant endpoints - GAN generates multi-tissue expression data revealing molecular associations - Improves data integration and predictability for precision medicine
Bertolini et al. (2020) [185]	- CRBMs create Digital Twins simulating AD clinical records under standard care - Trained on large datasets across AD spectrum - Captures progression of key clinical trial endpoints
Bertolini et al. (2021) [186]	- ML models forecast disease progression in MCI and AD - Improves clinical trial design by reducing sample size or increasing power - Covers broad baseline disease severity from MCI to mild-moderate AD
Bosl et al. (2024) [187]	- Proposes dynamical neuroelectric field as cognitive function substrate - Time series from scalp sensors quantify field and derive digital biomarkers - Provides robust framework for neuropsychiatric biomarker discovery
Buegler et al. (2020) [188]	- Digital biomarker models classify dementia progression risk within 3 years - Accurately identify amyloid neuropathology and cognitive decline rates - External validation shows 91% ROC-AUC for dementia progression
Calderone et al. (2024) [189]	- AI/ML revolutionize motor rehabilitation and diagnosis for stroke, SCI, PD - Enhance clinical assessments through objective measurement and real-time feedback - Integration of robotics with AI enables adaptive rehabilitation protocols
Cavedoni et al. (2020) [190]	- Integrated approach for early MCI detection using gait kinematics, VR, and ML - Provides continuous assessment compliant with DSM-5 guidelines - Behavioral data in VR environments enables ecological validity
Cellina et al. (2023) [191]	- DHTs improve organizational management and resource allocation in healthcare - Monitor disease progression by modeling genetic-environmental interactions - Potential to revolutionize healthcare through personalized medicine
Chen et al. (2024) [192]	- Tools for personalized cognitive health profiles and shared decision-making feasible - Computational techniques enrich understanding of individual treatment needs - Patient-centered approach improves engagement and adherence
Chudzik et al. (2024) [193]	- Digital biomarkers and ML identify pre-symptomatic neurodegenerative indicators - Eye tracking and facial analysis differentiate patients from controls - Integration could revolutionize diagnosis and improve quality of life
Crețu et al. (2024) [194]	- AI enhances accuracy and efficiency in Alzheimer’s diagnosis/prognosis - Biomarkers crucial for early detection with AI improving analysis - AI identifies subtle brain changes before clinical symptoms
Dagum (2018) [195]	- Smartphone passive measures serve as continuous neuropsychological assessment - High classification accuracy for depression diagnosis - Statistical models show high diagnostic accuracy for clinical outcomes
d’Aloisio et al. (2024) [196]	- Novel DTMS integrates diverse data into unified predictive model - Provides real-time insights into MS progression and treatment efficacy - Uses federated learning and explainable AI for personalized recommendations
de Kerckhove (2021) [197]	- Personal digital twin concept extends to individual behavior monitoring - Technology converges to replicate human data and life history - Cognitive functions externalized to digital systems
de la Paz Scribano Parada et al. (2024) [198]	- Verbal episodic memory is sensitive preclinical AD marker - Executive functions and processing speed significant in preclinical phase - VR and AI show promise but require validation and regulation
Dolciotti et al. (2025) [199]	- 3D brain organoids offer transformative AD study platform - Patient-specific iPSC-derived organoids model AD progression in real-time - Integration with technology enhances personalized medicine approaches
Domínguez-Fernández et al. (2023) [200]	- Reviews neuroimaging (MRI, PET) role in neurodegenerative diagnosis - Peripheral biomarkers crucial for early detection and monitoring - AI integration improves predictive models for early diagnosis
Dorsey et al. (2017) [201]	- Current neurodegenerative measures are subjective and episodic - Digital biomarkers provide objective, high-frequency data - Can accelerate therapeutic development and evaluation
Etekochay et al. (2024) [202]	- Neuroimaging (PET, MRI) significantly improves AD understanding - ML/AI integration enhances predictive diagnostic capacity - Enables early detection and intervention for improved quality of life
Fabbrizzi et al. (2025) [203]	- FEDE pipeline generates anatomically accurate brain digital twins - Successfully created ASD toddler digital twin with high precision - Identified aberrant excitation/inhibition ratios in ASD
Fekonja et al. (2024) [204]	- Digital twins model brain functions and pathology insights - Help understand brain tumor impact for personalized therapy - Bridge theoretical concepts and practical neuroscience reality
Gabrielli et al. (2023) [205]	- Framework for digital therapeutics using digital twins and virtual coaching - Combines digital twins with conversational AI for mental health - Addresses design optimization and effectiveness challenges
Geraci et al. (2024) [206]	- Uses smartphones/wearables for AD characterization and digital biomarkers - Creates comprehensive cross-domain datasets with privacy protection - Deep molecular phenotyping enhances biological mechanism understanding
Govindarajan et al. (2024) [207]	- Deep learning framework for early neurodegenerative detection - Integrates MRI, PET, clinical data using CNNs and RNNs - Achieves 92% accuracy rate in rigorous validation
Gulia et al. (2024) [208]	- ML models create enhanced Digital Twins for personalized healthcare - Enable predictive, preventive, and personalized care strategies - Benefits include early prevention, optimized treatment, reduced costs
Guo et al. (2025) [209]	- DTB emerges as transformative paradigm integrating multimodal data - Advances understanding of structure-function relationships and disease mechanisms - Captures functional heterogeneity and predicts treatment outcomes
Gupta et al. (2024) [210]	- AI/ML gain attention for early-stage AD detection ability - Addresses ethical considerations: privacy, consent, bias - Explainable AI systems developed to avoid bias and unethical conduct
Hashemi et al. (2024) [211]	- VBTs enhance understanding of brain mechanisms - Provide predictive capabilities for personalized treatments - Simulate various conditions to advance neurological disorder treatment
Huang et al. (2022) [212]	- Building research path for identifying cognition-related digital biomarkers - Digital phenotyping as effective tool for early cognitive deficit identification - Combination of hardware/software solutions to track cognition deficits
Iaboni et al. (2022) [213]	- Wearable multimodal sensors develop personalized ML models - Detect individual patterns of Behavioral and Psychological Symptoms of Dementia - Personalized approach to BPSD detection and monitoring
Imoh et al. (2024) [214]	- Multimodal neuroimaging with AI enhances early ASD detection - Deep learning improves diagnostic precision - Holds promise for forecasting individual developmental outcomes
Iturria-Medina et al. (2018) [215]	- Introduces personalized Therapeutic Intervention Fingerprint (pTIF) - pTIF outperforms cognitive/clinical evaluations in predicting gene expression - Links brain dynamics, treatment responses, and molecular alterations
Kamel Boulos et al. (2021) [216]	- Digital twins transform EHRs enabling precision medicine - Facilitate learning, hypothesis generation, and testing - Act as social equalizer in public health with personalized treatments
Kourtis et al. (2019) [217]	- Mobile/wearable technologies offer promising AD detection approach - Provide continuous, objective data collection - Multiple device metrics enhance AD progression forecasting
Libon et al. (2025) [218]	- Digital neuropsychological assessment enables early neurocognitive detection - Latency measures distinguish groups when scores are normal - Time-derived behaviors identify cognitive decline earlier than traditional methods
Łukaniszyn et al. (2024) [219]	- Digital twins rapidly developed for patients using AI - Predict future health outcomes with real-time updates - Improve care through simulated trials, disease prediction, remote monitoring
Lyall et al. (2023) [220]	- Applied modeling in dementia has substantial public health benefits - Digital health tools provide objective measurements - Digital mobility markers complement traditional assessments
Mandal et al. (2018) [221]	- Comprehensive AI strategy for early predictive AD diagnosis - Brain metabolic, structural, and behavioral pattern learning - Integration of imaging readouts with neuropsychological outcomes
Milner et al. (2024) [222]	- Traditional MCI biomarkers limited in reliability and scalability - Digital biomarkers create multidimensional profiles for different etiologies - Advanced ML techniques required for development and validation
Nisar et al. (2023) [223]	- Neuroimaging genetics significant for understanding ASD pathways - Detect structural, functional, metabolic changes - Integration with genomic data associates genetic markers with brain changes
Pal et al. (2025) [224]	- AI enables fast, accurate neurodegenerative disease diagnosis - AI-powered image analysis detects subtle brain alterations - Epigenetic and transcriptional changes as early detection indicators
Papachristou et al. (2024) [225]	- Digital twins enhance disease understanding and treatment customization - Support precision medicine through genetic-environmental modeling - Revolutionize healthcare with comprehensive health data views
Petrova-Antonova et al. (2020) [226]	- Digital twin platform explores behavioral changes in cognitive disorders - Uses Big Data and AI for medical data analysis - Two components: diagnostics/rehabilitation and data aggregation/analytics
Raguraj (2025) [227]	- AI analyzes behavioral, linguistic, and physiological data for cognitive decline - Deep learning detects subtle MCI patterns before clinical manifestation - Supports personalized monitoring and population-wide screening
Rai et al. (2020) [228]	- Altoida ADPS app discriminates healthy controls from prodromal AD - Differentiates MCI converters from non-converters - Validates novel digital biomarker for cognitive decline
Ren et al. (2025) [229]	- Digital twins enhance AD drug discovery with real-time tracking - Applications include progression prediction, biomarker discovery, new targets - Promising approach for revolutionizing AD drug discovery
Rudroff et al. (2024) [230]	- AI/ML improve early AD diagnosis accuracy and reliability - Multi-modality studies enhance prediction performance - Longitudinal studies crucial but face standardization challenges
Rutkowski et al. (2021) [231]	- Achieved ~90% accuracy using RF and neural networks for dementia onset - MFDFA patterns from EEG differ between normal and MCI stages - Simple yet effective method for early dementia detection
Rutkowski et al. (2023) [232]	- ML analyzes EEG network topology for early dementia prognosis - Significant differences in network features between healthy and MCI - High accuracy suggests applications in progression prediction
Sabbagh et al. (2019) [233]	- Digital technologies offer new prospects for MCI diagnosis/management - Developed consensus for large-scale MCI screening algorithms - Early detection crucial for disease-modifying therapies
Shah et al. (2023) [234]	- Evaluates ML (SVMs, CNNs) in neuropsychological research - SVMs robust for neuroimaging, CNNs excel in visual input - ML revolutionizes diagnosis enabling early, precise interventions
Sizemore et al. (2024) [235]	- Digital twin of infant microbiome forecasts ecosystem trajectories - Identifies infants at risk of poor head circumference growth - Early transplantation may mitigate risk for significant portion of the cohort
Song et al. (2025) [236]	- Digital biomarkers offer noninvasive alternative to traditional biomarkers - Derived from EEG, eye movement, gait, speech analysis - Smartphones and wearables emerging as diagnostic tools
Souillard-Mandar et al. (2021) [237]	- DCTclock improves early cognitive impairment detection - Higher AUC and sensitivity/specificity than MMSE - Differentiates various cognitive impairment stages including MCI subtypes
Sprint et al. (2024) [238]	- HDTwin achieves 0.81 peak accuracy for cognitive diagnoses - Multiple information sources fusion superior to single sources - Enhances accuracy, explainability, and early detection strategies
Tacchino et al. (2023) [239]	- Digital twins enhance MS cognitive phenotyping - Tele-cognitive-rehabilitation improves accessibility and effectiveness - Integration improves outcomes and transfers to everyday activities
Tang et al. (2020) [240]	- Adaptive AI dialogue agent adjusts questioning for MCI screening - Cost-effective and scalable for large-scale preclinical screening - Achieves comparable or better performance than traditional assessments
Tarnanas et al. (2018) [241]	- Predicts AD development 18–24 months before symptoms - NMI tracks micro-errors with >94% diagnostic accuracy for MCI - Detects subtle motor planning alterations preceding clinical symptoms
Tarnanas et al. (2021) [242]	- Common digital neuro signature identified for AD and PD - Contains 20+ shared features capturing motor/non-motor symptoms - Model identifies therapy-eligible patients using SHAP values
Termine et al. (2021) [243]	- Deep learning enhances understanding of neurodegenerative diseases - Identifies biomarkers using handwriting, speech, movement dynamics - Crucial for precision medicine and personalized interventions
Thangaraj et al. (2024) [244]	- Digital twins advance cardiovascular clinical decision-making - Evolving with new data modalities and AI advances - Highlights future applications and ethical considerations
Tortora et al. (2025) [245]	- Digital twins increasing in health sector applications - Big data and AI accelerate research and development - Healthcare applications still in infancy, neurology as cutting-edge area
Tosun (2025) [246]	- Develops multi-disciplinary/modality biomarkers for early detection - Detects subtle changes before clinical symptoms - Aims to improve outcomes through early intervention
Voigt et al. (2021) [247]	- Digital twins key advancement for individualized MS management - Combined with tele-rehabilitation improves cognitive outcomes - Delivers more effective and accessible tailored interventions
Wang et al. (2024) [248]	- Virtual brain twins are personalized, generative, adaptive models - Personalization involves brain assembly, connectivity mapping, ML parameters - Applied to healthy aging and five clinical diseases
Wang et al. (2024) [249]	- TWIN-GPT enhances clinical trial outcome prediction accuracy - Demonstrates exceptional fidelity, utility, and privacy performance - Provides practical evidence for digital twin healthcare applications
Wickramasinghe et al. (2022) [250]	- Digital twins improve dementia care precision and personalization - Developed as clinical decision support tool - Aligns with value-based healthcare addressing growing needs
Xiong et al. (2023) [251]	- DTB offers insights into intelligence and neurological disorders - Provides new paradigm for brain function understanding - Applications in neuromodulation and drug development
Yousefi et al. (2024) [252]	- ML algorithms show promise surpassing traditional diagnostics - Identified 12 algorithms for dementia, 18 for PD, 14 for MCI - Integration with conventional practice crucial for future advances

**Table 4:** Comprehensive Framework Performance and Clinical Outcomes.

Framework/Biomarker Integration	Biomarker Types	Algorithms Used	Performance Metrics	Clinical Impact	Implementation Status
Digital Twin for MS (DTMS)	Neuroimaging + Physiological + Behavioral	Unified predictive model	22 relevance matches	Precision MS management (+35% treatment outcomes)	Clinical deployment
Explainable Digital Phenotyping	Keystroke dynamics, Motor symptoms, Social markers	Ensemble regression (neuroQWERTY), Bayesian networks	89% early PD detection, 90% dementia prediction	25% faster diagnosis	Research validation
Multimodal Early Detection	EEG + fMRI + DTI + Digital phenotyping	XGBoost, CNN, GNN, Deep learning ensembles	85–95% accuracy (vs. 70–85% single modality)	40% improvement in early detection sensitivity	Multi-site trials
Personalized Cognitive Models	Speech, VR-based assessments, Smartphone data	SVM, Naïve Bayes, Decision trees	r = 0.82 stability over 6 months	15–20% reduction in disease progression	Pilot studies
Brain Structure Digital Twins	Anatomical MRI + Connectivity maps	FEDE pipeline, Graph Neural Networks, GANs	95% structural fidelity, 88–92% prediction accuracy	Real-time monitoring capability	Proof of concept
Continuous Monitoring Systems	Circadian rhythms, Sleep patterns, Daily activities	CRBMs, Signal processing, Tensor factorization	82–90% longitudinal accuracy	Timely intervention adjustments	Development phase

**Table 5:** Algorithm Optimization Strategies and Implementation Challenges.

Implementation Aspect	Challenge/Requirement	Solution Approach	Technical Specifications	Success Metrics	Recommendations
Data Integration	Harmonizing heterogeneous biomarkers across modalities	Cross-modal registration, Preprocessing pipelines	Hierarchical fusion, Parallel processing, Adaptive weighting	85% harmonization success, 15–20% accuracy improvement	Use ComBat-style harmonization for multi-site data
Algorithm Selection	Balancing accuracy vs. interpretability vs. computational cost	Hybrid approaches (Deep learning + Traditional ML)	CNN+SVM: 92–95% accuracy; XGBoost: 85–93% with interpretability; GNN: 88–92% for connectivity	Model quantization reduces memory 50–75%	Deep learning for complex patterns; Ensemble methods for interpretability
Personalization	Individual variability and model adaptation	Transfer learning, Federated learning, Adaptive algorithms	Pre-train on population → Fine-tune individually; Dynamic adaptation every 24–48 h	82% successful adaptation rate; Privacy 90% preserved	Implement continuous learning frameworks
Clinical Translation	Real-time processing and interpretability requirements	Edge computing, Explainable AI, Model compression	FP32 → FP16 quantization; 3–5× faster inference; Glass-box models	75% achieve real-time; 85% provide clinical insights	Prioritize interpretable models for clinical adoption
Validation & Reproducibility	Cross-site validation and longitudinal stability	Standardized protocols, Adversarial training	ICC improved from 0.72 to 0.89; External validation on 3+ sites	7–15% improved generalization; Maintained over 6 months	Implement standardized biomarker protocols

**Table 6:** Comprehensive Analysis of AI-Driven Digital Twin Models for Cognitive Decline Detection: Distribution, Performance, and Technologies.

Research Category	*N* (%)	Key Performance Metrics	Technologies/Methods	Clinical Applications
Digital Twin Models	29 (37.2%)	- Disease progression modeling with individual-specific parameters - Personalized trajectory forecasting - Real-time adaptation to patient data - Virtual patient representations	- Conditional Restricted Boltzmann Machines (CRBM) - Graph representation learning - Large Language Model integration - Dynamical systems modeling	- Alzheimer’s Disease progression - Multiple Sclerosis management - MCI to dementia conversion - Precision medicine optimization - Treatment response prediction
AI/ML Cognitive Assessment	37 (47.4%)	- Diagnostic accuracy: 90–98.27% - Early detection: 2–5 years pre-clinical	- Deep Learning (CNN/RNN architectures) - Traditional ML (RF/SVM/LR) - Ensemble methods - Error tracking algorithms - Multi-feature kernel learning	- Pre-clinical detection - Diagnostic classification - Risk stratification - Continuous monitoring - Screening automation
Digital Biomarkers	21 (26.9%)	- Multi-modal data integration - 24/7 continuous monitoring - Ecological validity measures - Individual baseline establishment - Remote accessibility	- Wearable sensors (n = 8) - EEG/Neurophysiology (n = 6) - Speech/Language analysis (n = 5) - Smartphone passive data (n = 5) - Gait/Mobility tracking (n = 4) - Eye tracking systems (n = 1)	- Remote patient monitoring - Daily activity assessment - Subtle change detection - Real-world functioning - Population screening
Comparative Studies	13 (16.7%)	- AI accuracy: 90–98% - Traditional methods: 70–85% - Detection advantage: 2–5 years - Cost reduction: ~60% - Monitoring frequency: 12.5× increase	- Head-to-head comparisons - Systematic reviews - Meta-analyses - Clinical validation studies - Health economics analyses	- Performance benchmarking - Clinical integration planning - Workflow optimization - Cost-effectiveness analysis
Ethical/Regulatory	8 (10.3%)	- Privacy protection frameworks - Model explainability metrics - Regulatory compliance guidelines - Data governance protocols	- Federated learning architectures - Explainable AI (XAI) methods - Blockchain for data integrity - Dynamic consent models	- GDPR compliance - Clinical adoption frameworks - Trust building mechanisms - Stakeholder engagement

**Table 7:** Implementation Framework for AI-Driven Cognitive Assessment: Current State, Advantages, Challenges, and Future Directions.

Implementation Domain	Current State	Quantified Advantages over Traditional Methods	Challenges & Gaps	Future Directions	Evidence Base
Detection & Diagnosis	- Achieved 90–98% diagnostic accuracy - 2–5 years earlier detection capability - Multiple validated algorithms in use - Pilot implementations in clinical settings	- Continuous monitoring (24/7) vs. episodic assessment (3–12 months) - Objective measurement vs. subjective clinical judgment - Individual-specific baselines vs. population norms - Multi-modal integration (6+ data types) vs. single modality - Automated analysis vs. manual interpretation	- Limited large-scale validation cohorts (most n < 500) - Lack of standardized protocols across studies - Insufficient diversity in study populations - Clinical validation for specific subtypes needed - Integration with existing diagnostic criteria	- Multi-site international trials (n > 10,000) - Development of unified assessment protocols - FDA/EMA regulatory approval pathways - Subtype-specific algorithm development - Real-world effectiveness studies	[175,182,188,207,227,241]
Technology Integration	- 6 digital biomarker types deployed - 29 operational digital twin platforms - Real-world data collection established - Cloud-based infrastructure emerging	- Population-level scalability vs. resource-intensive clinical assessment - 60% cost reduction per assessment - Ecological validity through naturalistic data - Remote accessibility for underserved populations - Automated data processing and analysis	- Technical complexity for end-users - Infrastructure requirements (connectivity, devices) - Interoperability between systems - Data storage and computational demands - Technology literacy barriers	- Edge computing for real-time processing - 5G integration for seamless connectivity - Standardized APIs for system integration - Low-cost device development - User-centered interface design	[195,196,218,226,248]
Clinical Translation	- Pilot programs in specialized centers - Augmented decision support tools - Risk stratification algorithms deployed - Early adopter clinician networks	- Personalized baselines for each patient - Real-time adaptation to disease progression - Predictive trajectories for intervention planning - Automated screening reducing clinical burden - Enhanced patient-clinician communication	- Workflow integration complexity - Clinician training requirements - Reimbursement model uncertainty - Liability and malpractice concerns - Resistance to technology adoption	- Clinical practice guidelines development - Comprehensive training programs - Health economics evidence generation - Hybrid human-AI decision models - Change management strategies	[177,220,233,239,251]
Data & Privacy	- GDPR-compliant frameworks developed - Federated learning pilots initiated - Consent management systems - Encryption standards implemented	- Secure multi-party computation - Patient control over data sharing - Transparent processing algorithms - Audit trails for data usage - Minimal data collection principles	- Data ownership ambiguity - Cross-border data transfer restrictions - Long-term data storage costs - Re-identification risks - Consent for future use scenarios	- Blockchain for immutable audit trails - Homomorphic encryption deployment - Dynamic consent platforms - International data governance frameworks - Privacy-preserving analytics	[195,197,210,245,249]
Patient Outcomes	- Earlier therapeutic interventions - Improved quality of life metrics - Reduced caregiver burden reported - Enhanced patient engagement	- Empowered self-monitoring capabilities - Timely intervention opportunities - Personalized care plan development - Objective progress tracking - Shared decision-making support	- Digital divide affecting access - Variable user acceptance rates - Technology anxiety in elderly - Limited outcome studies - Caregiver integration challenges	- Universal design principles - Digital health literacy programs - Caregiver-inclusive platforms - Patient-reported outcome integration - Longitudinal effectiveness trials	[192,213,216,219,225]

**Table 8:** Comprehensive Framework for Clinical Translation of Digital Twin Cognitive Models: From Challenges to Solutions.

Domain	Current Challenges	Technical Solutions	Clinical Applications	Performance/Impact	Key Studies
Algorithm & Interpretability	- “Black box” deep learning - Limited clinical trust - Complex decision paths	- Explainable AI frameworks - Hybrid statisticalDL models - Bayesian networks - Attention mechanisms	- Early PD detection (motor) - Dementia risk stratification - Cognitive decline prediction - Treatment response forecasting	- 90% accuracy (speech analysis) - 85–92% AUC (risk prediction) - High clinical interpretability - 95% CI coverage	[176,182,188,190,210,247]
Data Integration & Processing	- Heterogeneous sources - Real-time demands - “In-the-wild” vs. clinical - Multimodal complexity	- Graph Neural Networks - Ensemble methods (XGBoost) - Tensor factorization - Stream processing architectures	- Continuous symptom monitoring - Gait freezing detection - Fine motor assessment - Behavioral phenotyping	- 92% episode detection - 88% network accuracy - 70% dimensionality reduction - Real-time capability	[184,187,193,196,227,235]
Validation & Generalization	- Limited diversity - Site-specific biases - Insufficient longitudinal data - Cross-site failures	- SMOTE for imbalanced data - Transfer learning - Federated learning approaches - Multi-site protocols	- Population-wide screening - Disease progression tracking - Personalized risk assessment - Outcome prediction	- 75–85% cross-site accuracy - 6-year longitudinal validation - Reduced site bias by 40% - Improved generalization	[185,186,188,220,222,233]
Clinical Integration	- Workflow disruption - Training requirements - Regulatory uncertainty - Infrastructure needs	- Co-creation frameworks - Staged deployment - Cloud-based solutions - Standardized protocols	- Point-of-care decisions - Remote monitoring - Clinical trial enrichment - Therapeutic planning	- 45% adoption rate - 8 h training time - 98.5% system uptime - Workflow efficiency +30%	[177,181,189,216,218,239,248,251]

Abbreviations: PD = Parkinson’s Disease; DL = Deep Learning; AUC = Area Under Curve; CI = Confidence Interval; SMOTE = Synthetic Minority Over-sampling Technique.

**Table 9:** Evidence-Based Digital Twin Interventions in Neuropsychological Disorders: Current Practice and Future Directions.

Intervention Category	Current Implementation	Target Populations	Demonstrated Outcomes	Future Developments	Research Priorities	Timeline
Digital Cognitive Training	- 30 min/day smartphone apps - AI-adaptive difficulty - Multimodal exercises - Progress tracking	- MCI/early dementia - At-risk elderly - Post-stroke - Diverse demographics	- Memory: +35% - Attention: +42% - Executive function: +38% - 12-month sustainability - QoL improvement: 35%	- VR/AR integration - Personalized content - Social gamification - Predictive adaptation	- Optimal dosing protocols - Long-term efficacy - Mechanism studies - Cost-effectiveness	2–3 years
Neuromodulation-Guided	- -tDCS + physical therapy - TMS (prefrontal targeting) - Closed-loop stimulation - Biomarker-guided protocols	- PD with MCI - Treatment-resistant - Motor impairment - Mood disorders	- 3-month sustained gains - Motor function: +45% - Mood improvement: 60% - Reduced medication: 25%	- Real-time optimization - Home-based devices - Combination protocols - Precision targeting	- Safety profiles - Optimal parameters - Patient selection - Regulatory approval	3–5 years
Adaptive Serious Games	- i-PROGNOSIS suite - Motor-cognitive games - Social interaction modules - Reinforcement learning	- Parkinson’s disease - Autism spectrum - Stroke rehabilitation - Pediatric populations	- Engagement: 78% - Motor skills: +40% - Cognitive: +35% - Emotional regulation: +30%	- Multiplayer platforms - Haptic feedback - Eye-tracking integration - Emotion AI	- Engagement strategies - Transfer to daily life - Cultural adaptation - Scalability	2–4 years
Lifestyle Interventions	- High-frequency exercise - Balance activities (dance) - Sleep optimization - Dietary monitoring	- All disease stages - Prevention cohorts - Community settings - Global populations	- 6-year slower progression - Gait quality: +50% - Sleep efficiency: +25% - Medication reduction: 20%	- Wearable integration - Personalized coaching - Community platforms - Behavioral nudging	- Adherence factors - Minimum effective dose - Biomarker correlation - Health economics	1–3 years
Integrated Multimodal	- Combined protocols - Sequential interventions - Precision medicine - Outcome monitoring	- Complex cases - Non-responders - Comorbidities - Severe impairment	- Superior to single: +35% - Personalized response: 72% - Reduced dropouts: 40% - Cost-effective at scale	- AI orchestration - Dynamic adjustment - Predictive modeling - Universal access	- Optimal combinations - Sequencing rules - Interaction effects - Implementation science	3–5 years

Abbreviations: MCI = Mild Cognitive Impairment; QoL = Quality of Life; VR/AR = Virtual Reality/Augmented Reality; tDCS = transcranial Direct Current Stimulation; TMS = Transcranial Magnetic Stimulation; PD = Parkinson’s Disease; AI = Artificial Intelligence. Note: Percentage improvements represent relative changes from baseline or compared to control groups in referenced studies. Timeline estimates are based on current research trajectories and regulatory considerations.

**Table 10:** Comparative Performance of AI Algorithms for Digital Biomarker Processing.

Algorithm Type	Accuracy Range	Key Papers	Strengths	Limitations	Best Use Cases
Deep Learning (CNNs, RNNs, LSTMs)	85–98.27%	[175,182,184,207,214,227]	- Automatic feature extraction - Superior multimodal integration - Complex pattern recognition	- Requires >1000 samples - High computational cost - Limited interpretability	- Large-scale screening - Multimodal data - Complex temporal patterns
Traditional ML (SVM, RF, LR)	70–85%	[188,190,196,232,234]	- Works with small datasets (50–100) - High interpretability - Low computational requirements	- Manual feature engineering - Lower accuracy - Limited complexity handling	- Small clinical studies - Resource-constrained settings - Interpretability required
Ensemble Methods (XGBoost, Bagging)	80–91%	[176,188,231]	- Balanced performance - Robust to overfitting - Handles heterogeneous data	- Moderate complexity - Partial interpretability	- Medium-scale applications - Risk stratification - Clinical decision support
Specialized Models (neuroQWERTY, CRBMs)	Variable	[176,185,186,241,242]	- Domain-specific optimization - Clinical validation	- Limited generalizability - Narrow application scope	- Disease-specific detection - Specialized biomarkers

**Table 11:** Technical Requirements and Deployment Considerations.

Criterion	Deep Learning	Traditional ML	Ensemble Methods
Data Requirements	>1000 samples	50–100 samples	200–500 samples
Computational Resources	GPU required, High memory	CPU sufficient, Low memory	Moderate GPU/CPU
Training Time	Hours to days	Minutes to hours	Hours
Inference Time	Milliseconds (with GPU)	Microseconds	Milliseconds
Interpretability	Low (requires post hoc methods)	High (direct coefficients)	Moderate
Update Frequency	Difficult (retraining needed)	Easy (incremental updates)	Moderate
Edge Deployment	Challenging	Excellent	Good
Cost per Deployment	High	Low	Moderate

**Table 12:** Biomarker-Algorithm Compatibility Matrix.

Digital Biomarker Type	Optimal Algorithm	Processing Approach	Key Features	Accuracy
Speech/Language	RNN, LSTM, NLP	Sequential analysis	Acoustic, lexical, syntactic features	90%
Keystroke Dynamics	neuroQWERTY (Ensemble)	Temporal patterns	HT, nFT, nP	85–91%
Imaging (MRI, fMRI)	CNN	Spatial feature extraction	Voxel-wise analysis	95–98.27%
EEG Signals	CNN + RNN hybrid	Spatiotemporal	Frequency bands, connectivity	85–95%
Accelerometer	Deep Learning	Time-series analysis	Gait patterns, tremor	85–90%
Smartphone Usage	Random Forest, SVM	Behavioral patterns	App usage, call frequency	70–80%

## Data Availability

Not applicable.

## Supplementary Materials

The following supporting information can be downloaded at https://www.mdpi.com/article/10.3390/biomimetics10100640/s1, Table S1, Full Table of the Studies (*n* = 78) included in the Systematic Review. Table S2, Validation Characteristics and Generalizability Assessment. Table S3, Abbreviation list. Table S4, Comprehensive Framework Performance with Validation Quality Assessment.
