# Supplementary material for: Digital Twin Cognition: AI-Biomarker Integration in Biomimetic Neuropsychology
Source: Biomimetics (Basel). 2025 Sep 23;10(10):640. doi: 10.3390/biomimetics10100640 (PMC12561581; doi:10.3390/biomimetics10100640)
Supplement: Supplementary file 1 [file biomimetics-10-00640-s001.zip › Sup_Table_S1_Full_Table.pdf]

Authors	Key Findings	Study Objectives	Methodology	Main Findings	Outcome Measured	Algorithms	Neuropsychological Assessment & Tools
Adarsh et al. (2024) [175]	-Achieves 98.27% classification accuracy for AD/MCI diagnosis<br>-Outperforms existing models across multiple metrics<br>-Explainable AI provides transparency enhancing clinical applicability	To achieve 98.27% classification accuracy for AD/MCI	Classification accuracy study	98.27% accuracy achieved; Outperforms existing models; Explainable AI enhances applicability	Classification accuracy (98.27%), model performance comparison, clinical applicability	CNN with Multi-feature Kernel SCDDL	AD/MCI classification tools, explainable diagnostic systems
Alfalahi et al. (2023) [176]	-Digital technologies and AI create disease-specific phenotypic explanations for neurodegenerative diseases<br>-Digital biomarkers detect motor symptoms in PD and cognitive decline in AD with high accuracy<br>-Highlights association of apathy with brain health changes	To analyze and validate computational models in AI for deciphering phenotypic and temporal heterogeneity of neurodegenerative diseases	Scoping review of explainable digital phenotyping approaches	Digital technologies and AI create disease-specific phenotypic explanations; Digital biomarkers detect motor symptoms in PD and cognitive decline in AD with high accuracy; Association of apathy with brain health changes	Motor symptom detection accuracy, cognitive decline markers, apathy-brain health correlations	neuroQWERTY (ensemble regression), multivariate ML models, deep learning, Bayesian networks, Monte Carlo Dropout	Keystroke dynamics, speech analysis, gait assessment, apathy scales, motor symptom tracking
Alouthah et al. (2024) [177]	-AI integration enhances diagnosis and personalized care for cognitive impairments<br>-Identifies AI-driven interventions: personalized apps, VR treatments, social robots<br>-Challenges include technical limitations, data privacy, and need for	To evaluate AI integration for diagnosis and personalized care of cognitive impairments	Systematic review of AI-driven interventions	AI enhances diagnosis and personalized care; Identifies personalized apps, VR treatments, social robots; Challenges include technical limitations and data privacy	Diagnostic accuracy, intervention effectiveness, implementation barriers	Machine Learning (ML), Reinforcement Learning (RL)	Personalized cognitive apps, VR-based assessments, social robot interactions

	standardized frameworks						
Antonelli et al. (2020) [178]	-Prenatal stress increases susceptibility to neurodevelopmental impairments<br>-Physiological and epigenetic biomarkers predict neurodevelopmental outcomes<br>-Early family-centered interventions crucial for preventing neuropsychiatric problems	To examine prenatal stress impact on neurodevelopmental outcomes and biomarker prediction	Longitudinal cohort study with biomarker analysis	Prenatal stress increases susceptibility to neurodevelopmental impairments; Physiological and epigenetic biomarkers predict outcomes; Early interventions crucial	Neurodevelopmental outcomes, biomarker predictive validity	Machine Learning (ML)	Neurodevelopmental assessments, stress exposure measures
Arghavani et al. (2025) [179]	-Porous materials (MOFs, COFs, MXene) enable early neurodegenerative disease diagnosis<br>-Integration into biosensors provides non-invasive, sensitive detection platforms<br>-AI/ML enhance diagnostic accuracy and biomarker interpretation	To evaluate porous materials for early neurodegenerative disease diagnosis	Technical review of biosensor integration	MOFs, COFs, MXene enable early diagnosis; Integration into biosensors provides non-invasive detection; AI/ML enhance diagnostic accuracy	Biosensor sensitivity, diagnostic accuracy, biomarker detection limits	AI/ML	Biosensor-based cognitive biomarker detection
Arya et al. (2023) [180]	-Traditional and digital biomarker integration through AI improves healthcare decisions<br>-Real-time sensing and AI models enhance outcomes<br>-Facilitates development of point-of-care diagnostics	To assess traditional and digital biomarker integration through AI for healthcare decisions	Mixed-methods review of biomarker integration	AI improves healthcare decisions; Real-time sensing enhances outcomes; Facilitates point-of-care diagnostics	Decision accuracy, clinical outcomes, diagnostic speed	AI-assisted biosensing	Point-of-care cognitive diagnostics, real-time sensing tools
Ashraf et al. (2024) [181]	-Digital twins enable real-time therapy	To develop digital twin framework for real-time therapy and disease tracking	Digital twin implementation study	Enables real-time therapy mimicry and progression tracking;	Therapy effectiveness, disease	Digital twin algorithms	Real-time neurological monitoring,

	mimicry and disease progression tracking<br>-Improves understanding of neurological illnesses for efficient treatments<br>-Enhances patient data management and clinical decision-making			Improves understanding of neurological illnesses; Enhances patient data management	progression tracking, clinical decision accuracy		therapy tracking systems
Babu et al. (2024) [182]	-Deep learning shows outstanding accuracy in early AD identification before symptoms<br>-Multimodal approaches (neuroimaging, clinical, genetic) provide comprehensive insights<br>-Blood-based and genetic biomarkers critical for diagnosis with need for AI explainability	To evaluate deep learning for early AD identification before symptoms	Comparative analysis of ML approaches	Outstanding accuracy in early AD identification; Multimodal approaches provide comprehensive insights; Blood-based and genetic biomarkers critical	Diagnostic accuracy (>90%), early detection sensitivity, biomarker validity	Deep learning, machine learning classifiers, ensemble approaches	Neuroimaging (MRI, PET), clinical assessments, genetic testing, blood biomarkers
Banks et al. (2024) [183]	-Multimodal digital biomarkers for clinical classification of memory/cognitive impairment<br>-Focus on early detection to improve healthcare outcomes for aging adults<br>-Enable early intervention and potential prevention of decline	To develop multimodal digital biomarkers for memory/cognitive impairment classification	Cross-sectional validation study	Successfully classifies memory/cognitive impairment; Improves healthcare outcomes for aging adults; Enables early intervention	Classification accuracy, early detection rates, intervention timing	Multimodal classification algorithms	Memory assessments, cognitive impairment screening tools
Barbiero et al. (2021) [184]	-Digital twin model successfully tested on clinical cases, forecasting relevant endpoints	To test digital twin model on clinical cases using GAN technology	Clinical validation with synthetic data generation	Successfully forecasts relevant endpoints; GAN generates multi-tissue expression data; Improves data	Endpoint prediction accuracy, data integration quality, clinical applicability	Graph Neural Networks (GNNs), Generative Adversarial Networks	Clinical endpoint assessments, multi-tissue expression profiling

	-GAN generates multi-tissue expression data revealing molecular associations<br>-Improves data integration and predictability for precision medicine			integration for precision medicine		(GANs), Graph Network Blocks	
Bertolini et al. (2020) [185]	-CRBMs create Digital Twins simulating AD clinical records under standard care<br>-Trained on large datasets across AD spectrum<br>-Captures progression of key clinical trial endpoints	To create Digital Twins simulating AD clinical records using CRBMs	Machine learning model development	CRBMs successfully simulate AD progression; Trained on large datasets across AD spectrum; Captures progression of key trial endpoints	Disease progression accuracy, clinical trial endpoint prediction	Conditional Restricted Boltzmann Machines (CRBMs)	Clinical Dementia Rating Sum-of-Boxes (CDR-SB), AD clinical trial endpoints
Bertolini et al. (2021) [186]	-ML models forecast disease progression in MCI and AD<br>-Improves clinical trial design by reducing sample size or increasing power<br>-Covers broad baseline disease severity from MCI to mild-moderate AD	To forecast disease progression in MCI and AD using ML models	Longitudinal predictive modeling	ML forecasts MCI/AD progression; Improves clinical trial design; Covers broad baseline severity	Progression prediction accuracy, sample size optimization, statistical power	Machine learning models for progression forecasting	MCI/AD progression scales, clinical trial outcome measures
Bosl et al. (2024) [187]	-Proposes dynamical neuroelectric field as cognitive function substrate<br>-Time series from scalp sensors quantify field and derive digital biomarkers<br>-Provides robust framework for neuropsychiatric biomarker discovery	To propose dynamical neuroelectric field as cognitive function substrate	Theoretical framework with empirical validation	Time series from scalp sensors quantify field; Derives digital biomarkers; Provides robust framework for biomarker discovery	Neurophysiologic al biomarker validity, cognitive function correlation	Tensor factorization, dynamical systems framework	EEG/scalp sensors, neurophysiologic al measures, cognitive function mapping

Buegler et al. (2020) [188]	-Digital biomarker models classify dementia progression risk within 3 years<br>-Accurately identify amyloid neuropathology and cognitive decline rates<br>-External validation shows 91% ROC-AUC for dementia progression	To classify dementia progression risk using digital biomarker models	Prospective cohort with ML classification	Classifies dementia risk within 3 years; Identifies amyloid neuropathology; 91% ROC-AUC for progression	Dementia progression risk (91% AUC), amyloid detection, cognitive decline rates	XGBoost classification with SMOTE	Dementia risk assessment, amyloid biomarkers, cognitive decline measures
Calderone et al. (2024) [189]	-AI/ML revolutionize motor rehabilitation and diagnosis for stroke, SCI, PD<br>-Enhance clinical assessments through objective measurement and real-time feedback<br>-Integration of robotics with AI enables adaptive rehabilitation protocols	To revolutionize motor rehabilitation using AI/ML for stroke, SCI, PD	Systematic review of rehabilitation technologies	AI/ML enhance clinical assessments; Provides objective measurement and real-time feedback; Robotics enable adaptive protocols	Motor function improvement, rehabilitation effectiveness, adaptation metrics	AI/ML for rehabilitation	Motor function assessments, robotic rehabilitation tools, real-time feedback systems
Cavedoni et al. (2020) [190]	-Integrated approach for early MCI detection using gait kinematics, VR, and ML<br>-Provides continuous assessment compliant with DSM-5 guidelines<br>-Behavioral data in VR environments enables ecological validity	To integrate gait kinematics, VR, and ML for early MCI detection	Multimodal assessment protocol development	Integrated approach detects early MCI; Compliant with DSM-5 guidelines; VR enables ecological validity	MCI detection accuracy, ecological validity measures, DSM-5 compliance	Naïve Bayes, Support Vector Machines, decision trees, linear regression	Gait kinematic analysis, Virtual Reality assessments, DSM-5 criteria
Cellina et al. (2023) [191]	-DHTs improve organizational management and resource allocation in healthcare	To evaluate DHTs for organizational management and resource allocation	Healthcare system implementation study	DHTs improve management and allocation; Monitor disease through genetic-	Resource allocation efficiency, disease monitoring accuracy,	Deep learning models, artificial neural networks	Healthcare monitoring systems, disease progression tracking

	-Monitor disease progression by modeling genetic-environmental interactions<br>-Potential to revolutionize healthcare through personalized medicine			environmental modeling; Potential for personalized medicine	organizational outcomes		
Chen et al. (2024) [192]	-Tools for personalized cognitive health profiles and shared decision-making feasible<br>-Computational techniques enrich understanding of individual treatment needs<br>-Patient-centered approach improves engagement and adherence	To develop tools for personalized cognitive health profiles and shared decision-making	Tool development and feasibility study	Tools for personalized profiles feasible; Enriches understanding of treatment needs; Improves engagement and adherence	Decision-making quality, patient engagement, treatment adherence	Computational methods for personalization	Personalized cognitive health profiling tools, shared decision-making instruments
Chudzik et al. (2024) [193]	-Digital biomarkers and ML identify pre-symptomatic neurodegenerative indicators<br>-Eye tracking and facial analysis differentiate patients from controls<br>-Integration could revolutionize diagnosis and improve quality of life	To identify pre-symptomatic neurodegenerative indicators using digital biomarkers and ML	Cross-sectional diagnostic study	ML identifies pre-symptomatic indicators; Eye tracking and facial analysis differentiate patients; Could revolutionize diagnosis	Pre-symptomatic detection accuracy, diagnostic sensitivity/specificity	Convolutional Neural Networks (CNNs), Rough Set Theory (RST), Fuzzy Rough Set Theory (FRST)	Eye tracking, facial expression analysis, pre-symptomatic screening
Crețu et al. (2024) [194]	-AI enhances accuracy and efficiency in Alzheimer's diagnosis/prognosis<br>-Biomarkers crucial for early detection	To enhance accuracy and efficiency in Alzheimer's diagnosis/prognosis using AI	Meta-analysis of AI diagnostic approaches	AI enhances diagnostic accuracy/efficiency; Biomarkers crucial with AI improving analysis; Identifies subtle brain changes	Diagnostic accuracy improvement, early detection rates, prognostic validity	AI algorithms for biomarker analysis	AD diagnostic tools, biomarker panels, brain change detection

	with AI improving analysis<br>-AI identifies subtle brain changes before clinical symptoms						
Dagum (2018) [195]	-Smartphone passive measures serve as continuous neuropsychological assessment<br>-High classification accuracy for depression diagnosis<br>-Statistical models show high diagnostic accuracy for clinical outcomes	To validate smartphone passive measures as neuropsychological assessment	Validation study with clinical correlation	Passive measures serve as continuous assessment; High accuracy for depression diagnosis; Shows high diagnostic accuracy	Depression classification accuracy, continuous monitoring validity	Signal processing algorithms, logistic regression with stepwise variable selection, multiple linear regression	Smartphone passive data collection (call frequency, app usage), depression scales
d'Aloisio et al. (2024) [196]	-Novel DTMS integrates diverse data into unified predictive model<br>-Provides real-time insights into MS progression and treatment efficacy<br>-Uses federated learning and explainable AI for personalized recommendations	To develop novel DTMS integrating diverse data for MS management	Digital twin platform development	DTMS integrates data into unified model; Provides real-time MS insights; Uses federated learning and XAI	MS progression prediction, treatment efficacy, personalized recommendations	Federated learning, explainable AI (XAI)	MS clinical assessments, MRI imaging, patient-reported outcomes
de Kerckhove (2021) [197]	-Personal digital twin concept extends to individual behavior monitoring<br>-Technology converges to replicate human data and life history<br>-Cognitive functions externalized to digital systems	To explore personal digital twin concept for behavior monitoring	Conceptual framework development	Extends to individual behavior monitoring; Technology converges to replicate human data; Cognitive functions externalized	Behavioral monitoring capability, data replication fidelity, cognitive externalization	Digital simulation algorithms	Behavioral monitoring systems, cognitive function tracking
de la Paz Scribano Parada et al. (2024) [198]	-Verbal episodic memory is sensitive preclinical AD marker<br>-Executive functions and processing speed	To identify verbal episodic memory as preclinical AD marker	Neuropsychological assessment study	Verbal memory sensitive to preclinical AD; Executive functions significant; VR/AI promising but need validation	Memory assessment sensitivity, executive function markers, VR/AI validity	AI and VR algorithms (validation needed)	Verbal episodic memory tests, executive function assessments,

	significant in preclinical phase<br>-VR and AI show promise but require validation and regulation					processing speed measures
Dolciotti et al. (2025) [199]	-3D brain organoids offer transformative AD study platform<br>-Patient-specific iPSC-derived organoids model AD progression in real-time<br>-Integration with technology enhances personalized medicine approaches	To develop 3D brain organoids for AD study platform	Laboratory validation study	Organoids offer transformative platform; Patient-specific models track progression; Enhances personalized medicine	AD progression modeling, real-time monitoring capability, personalized treatment response	Patient-specific modeling algorithms	3D brain organoid models, iPSC-derived assessments
Domínguez-Fernández et al. (2023) [200]	-Reviews neuroimaging (MRI, PET) role in neurodegenerative diagnosis<br>-Peripheral biomarkers crucial for early detection and monitoring<br>-AI integration improves predictive models for early diagnosis	To review neuroimaging role in neurodegenerative diagnosis	Systematic review of imaging biomarkers	MRI, PET crucial for diagnosis; Peripheral biomarkers important; AI improves predictive models	Diagnostic accuracy, biomarker sensitivity, AI model performance	AI-enhanced neuroimaging analysis	MRI, PET scanning, peripheral biomarker assessments
Dorsey et al. (2017) [201]	-Current neurodegenerative measures are subjective and episodic<br>-Digital biomarkers provide objective, high-frequency data<br>-Can accelerate therapeutic development and evaluation	To address limitations of current neurodegenerative measures	Critical review and framework proposal	Current measures subjective/episodic; Digital biomarkers provide objective data; Accelerates therapeutic development	Measurement objectivity, data frequency, therapeutic development speed	Digital biomarker algorithms	Digital vs traditional neuropsychological measures
Etekochay et al. (2024) [202]	-Neuroimaging (PET, MRI) significantly	To evaluate neuroimaging improvements in AD understanding	Imaging technology review	PET, MRI significantly improve understanding; ML/AI enhance	Diagnostic enhancement, predictive accuracy,	ML/AI for neuroimaging	PET imaging, MRI protocols, cognitive

	improves AD understanding -ML/AI integration enhances predictive diagnostic capacity -Enables early detection and intervention for improved quality of life			predictive capacity; Enables early intervention	intervention timing		assessment correlation
Fabbrizzi et al. (2025) [203]	-FEDE pipeline generates anatomically accurate brain digital twins -Successfully created ASD toddler digital twin with high precision -Identified aberrant excitation/inhibition ratios in ASD	To generate anatomically accurate brain digital twins using FEDE pipeline	Technical validation study	FEDE creates accurate brain twins; Successfully modeled ASD toddler; Identified aberrant excitation/inhibition	Anatomical accuracy, ASD identification, excitation/inhibiti on ratios	FEDE pipeline algorithms	ASD assessment tools, brain imaging, excitation/inhibit ion balance measures
Fekonja et al. (2024) [204]	-Digital twins model brain functions and pathology insights -Help understand brain tumor impact for personalized therapy -Bridge theoretical concepts and practical neuroscience reality	To model brain functions and pathology using digital twins	Brain tumor impact modeling	Digital twins model functions/pathology; Understand tumor impact; Bridge theory and practice	Brain function modeling accuracy, tumor impact assessment, therapeutic personalization	Brain modeling algorithms	Brain function assessments, tumor impact on cognition
Gabrielli et al. (2023) [205]	-Framework for digital therapeutics using digital twins and virtual coaching -Combines digital twins with conversational AI for mental health -Addresses design optimization and effectiveness challenges	To develop framework for digital therapeutics using twins and coaching	Framework development study	Combines twins with conversational AI; Addresses design and effectiveness; Focus on mental health	Mental health outcomes, coaching effectiveness, design optimization	Conversation al AI algorithms	Mental health assessments, virtual coaching tools

Geraci et al. (2024) [206]	-Uses smartphones/wearables for AD characterization and digital biomarkers<br>-Creates comprehensive cross-domain datasets with privacy protection<br>-Deep molecular phenotyping enhances biological mechanism understanding	To use smartphones/wearables for AD characterization and biomarkers	Multi-device data collection study	Creates comprehensive datasets; Privacy protection maintained; Deep phenotyping enhances understanding	AD characterization accuracy, privacy compliance, biological mechanism insights	Deep molecular phenotyping algorithms	Smartphone sensors, wearable devices, AD phenotyping tools
Govindarajan et al. (2024) [207]	-Deep learning framework for early neurodegenerative detection<br>-Integrates MRI, PET, clinical data using CNNs and RNNs<br>-Achieves 92% accuracy rate in rigorous validation	To develop deep learning framework for early neurodegenerative detection	Framework validation study	Integrates MRI, PET, clinical data; Uses CNNs and RNNs; 92% accuracy rate	Detection accuracy (92%), multimodal integration effectiveness	Convolutional Neural Networks (CNNs), Recurrent Neural Networks (RNNs)	MRI, PET, clinical cognitive assessments
Gulia et al. (2024) [208]	-ML models create enhanced Digital Twins for personalized healthcare<br>-Enable predictive, preventive, and personalized care strategies<br>-Benefits include early prevention, optimized treatment, reduced costs	To create enhanced Digital Twins using ML for personalized healthcare	ML model development study	Enables predictive/preventive care; Benefits include early prevention; Optimized treatment, reduced costs	Prediction accuracy, prevention effectiveness, cost reduction metrics	Machine learning models for digital twins	Personalized health assessments, preventive screening tools
Guo et al. (2025) [209]	-DTB emerges as transformative paradigm integrating multimodal data<br>-Advances understanding of structure-function	To advance DTB as transformative paradigm integrating multimodal data	Paradigm development study	DTB integrates multimodal data; Advances structure-function understanding; Predicts treatment outcomes	Functional heterogeneity capture, treatment outcome prediction	Multimodal integration algorithms	Multimodal neuropsychological batteries, brain structure-function mapping

	relationships and disease mechanisms<br>-Captures functional heterogeneity and predicts treatment outcomes					
Gupta et al. (2024) [210]	-AI/ML gain attention for early-stage AD detection ability<br>-Addresses ethical considerations: privacy, consent, bias<br>-Explainable AI systems developed to avoid bias and unethical conduct	To address ethical considerations in AI/ML for early AD detection	Ethical framework analysis	AI/ML attention for early detection; Addresses privacy, consent, bias; Explainable AI developed	Ethical compliance, bias reduction, explainability metrics	Explainable AI systems	Early AD detection tools with ethical safeguards
Hashemi et al. (2024) [211]	-VBTs enhance understanding of brain mechanisms<br>-Provide predictive capabilities for personalized treatments<br>-Simulate various conditions to advance neurological disorder treatment	To enhance brain mechanism understanding using VBTs	Virtual brain twin development	VBTs enhance understanding; Provide predictive capabilities; Simulate various conditions	Brain mechanism accuracy, treatment personalization, condition simulation fidelity	Virtual brain simulation algorithms	Virtual brain assessment tools, neurological condition simulators
Huang et al. (2022) [212]	-Building research path for identifying cognition-related digital biomarkers<br>-Digital phenotyping as effective tool for early cognitive deficit identification<br>-Combination of hardware/software solutions to track cognition deficits	To build research path for cognition-related digital biomarkers	Research framework development	Digital phenotyping effective tool; Identifies early cognitive deficits; Hardware/software track deficits	Cognitive deficit identification, biomarker validity, tracking accuracy	Digital phenotyping algorithms	Digital phenotyping platforms, cognitive deficit tracking tools
Iaboni et al. (2022) [213]	-Wearable multimodal sensors develop personalized ML models	To develop personalized ML models using wearable sensors for BPSD	Personalized model development	Detects individual BPSD patterns; Wearable sensors enable ML models;	BPSD detection accuracy, pattern recognition, personalized monitoring	Personalized machine learning models	Wearable sensors for BPSD, behavioral monitoring devices

	-Detect individual patterns of Behavioral and Psychological Symptoms of Dementia<br>-Personalized approach to BPSD detection and monitoring			Personalized approach effective		
Imoh et al. (2024) [214]	-Multimodal neuroimaging with AI enhances early ASD detection<br>-Deep learning improves diagnostic precision<br>-Holds promise for forecasting individual developmental outcomes	To enhance early ASD detection using multimodal neuroimaging with AI	Diagnostic enhancement study	Deep learning improves precision; Multimodal approach enhances detection; Forecasts developmental outcomes	ASD detection accuracy, diagnostic precision, outcome prediction	Deep learning for multimodal neuroimaging	Multimodal neuroimaging, ASD diagnostic batteries, developmental assessments
Iturria-Medina et al. (2018) [215]	-Introduces personalized Therapeutic Intervention Fingerprint (pTIF)<br>-pTIF outperforms cognitive/clinical evaluations in predicting gene expression<br>-Links brain dynamics, treatment responses, and molecular alterations	To introduce personalized Therapeutic Intervention Fingerprint (pTIF)	pTIF development and validation	pTIF outperforms cognitive evaluations; Links brain dynamics to treatment; Predicts gene expression	Gene expression prediction, treatment response correlation, molecular alteration links	pTIF algorithm	Personalized cognitive evaluations, brain dynamics assessments
Kamel Boulos et al. (2021) [216]	-Digital twins transform EHRs enabling precision medicine<br>-Facilitate learning, hypothesis generation, and testing<br>-Act as social equalizer in public health with	To transform EHRs using digital twins for precision medicine	Healthcare transformation analysis	Digital twins enable precision medicine; Facilitate learning and testing; Act as social equalizer	Precision medicine capability, hypothesis testing, healthcare equity	Digital twin EHR algorithms	EHR-integrated cognitive assessments

	personalized treatments						
Kourtis et al. (2019) [217]	-Mobile/wearable technologies offer promising AD detection approach<br>-Provide continuous, objective data collection<br>-Multiple device metrics enhance AD progression forecasting	To evaluate mobile/wearable technologies for AD detection	Technology assessment study	Promising approach for AD detection; Continuous objective data collection; Multiple metrics enhance forecasting	AD detection accuracy, data continuity, progression forecasting	Mobile/wearable sensor algorithms	Mobile devices, wearable sensors, continuous cognitive monitoring
Libon et al. (2025) [218]	-Digital neuropsychological assessment enables early neurocognitive detection<br>-Latency measures distinguish groups when scores are normal<br>-Time-derived behaviors identify cognitive decline earlier than traditional methods	To enable early neurocognitive detection using digital assessment	Digital assessment validation	Latency measures distinguish groups; Time-derived behaviors identify decline; Earlier than traditional methods	Early detection sensitivity, latency discrimination, temporal behavior metrics	Time-based digital assessment algorithms	Digital neuropsychological assessment, latency measures, time-derived behaviors
Łukaniszyn et al. (2024) [219]	-Digital twins rapidly developed for patients using AI<br>-Predict future health outcomes with real-time updates<br>-Improve care through simulated trials, disease prediction, remote monitoring	To rapidly develop patient digital twins using AI	AI development methodology	AI enables rapid twin development; Predicts health outcomes real-time; Improves care through trials/monitoring	Health outcome prediction, real-time update capability, care improvement metrics	AI for rapid digital twin development	Rapid cognitive assessment protocols, real-time monitoring
Lyall et al. (2023) [220]	-Applied modeling in dementia has substantial public health benefits<br>-Digital health tools provide objective measurements<br>-Digital mobility markers complement	To apply modeling in dementia for public health benefits	Public health modeling study	Substantial public health benefits; Digital tools provide objective measures; Mobility markers complement assessments	Public health impact, measurement objectivity, mobility marker validity	Traditional machine learning (logistic regression, decision trees)	Digital mobility markers, objective measurement tools

	traditional assessments						
Mandal et al. (2018) [221]	-Comprehensive AI strategy for early predictive AD diagnosis<br>-Brain metabolic, structural, and behavioral pattern learning<br>-Integration of imaging readouts with neuropsychological outcomes	To develop comprehensive AI strategy for early AD diagnosis	AI strategy development	Brain metabolic/structural learning; Pattern recognition effective; Integrates imaging with outcomes	Predictive diagnosis accuracy, pattern learning effectiveness, outcome integration	AI pattern recognition algorithms	Brain metabolic assessments, structural imaging, neuropsychological outcomes
Milner et al. (2024) [222]	-Traditional MCI biomarkers limited in reliability and scalability<br>-Digital biomarkers create multidimensional profiles for different etiologies<br>-Advanced ML techniques required for development and validation	To address limitations of traditional MCI biomarkers	Biomarker comparison study	Traditional biomarkers limited; Digital create multidimensional profiles; Advanced ML required	Biomarker reliability, scalability metrics, ML performance requirements	Advanced ML techniques	Traditional vs digital MCI biomarkers, multidimensional profiling
Nisar et al. (2023) [223]	-Neuroimaging genetics significant for understanding ASD pathways<br>-Detect structural, functional, metabolic changes<br>-Integration with genomic data associates genetic markers with brain changes	To understand ASD pathways using neuroimaging genetics	Neuroimaging genetics study	Significant for ASD understanding; Detects structural/functional changes; Associates genetics with brain	ASD pathway detection, genetic-brain associations, structural change identification	Neuroimaging genetics algorithms	Neuroimaging protocols, genetic testing, ASD assessments
Pal et al. (2025) [224]	-AI enables fast, accurate neurodegenerative disease diagnosis<br>-AI-powered image analysis detects	To enable fast, accurate neurodegenerative diagnosis using AI	AI diagnostic speed study	AI enables fast/accurate diagnosis; Detects subtle brain alterations; Epigenetic changes as indicators	Diagnostic speed, accuracy metrics, early detection indicators	AI-powered image analysis	Rapid diagnostic tools, brain alteration detection, epigenetic markers

	subtle brain alterations<br>-Epigenetic and transcriptional changes as early detection indicators						
Papachristou et al. (2024) [225]	-Digital twins enhance disease understanding and treatment customization<br>-Support precision medicine through genetic-environmental modeling<br>-Revolutionize healthcare with comprehensive health data views	To enhance disease understanding using digital twins	Disease modeling study	Enhances understanding and customization; Supports precision medicine; Revolutionizes healthcare	Disease understanding metrics, treatment customization, healthcare transformation	Digital twin modeling algorithms	Comprehensive health data assessments, precision medicine tools
Petrova-Antonova et al. (2020) [226]	-Digital twin platform explores behavioral changes in cognitive disorders<br>-Uses Big Data and AI for medical data analysis<br>-Two components: diagnostics/rehabilitation and data aggregation/analytics	To explore behavioral changes using digital twin platform	Platform development study	Platform explores cognitive disorders; Uses Big Data and AI; Two-component system	Behavioral change detection, data analytics capability, system integration	Big Data and AI analytics	Behavioral change monitoring, cognitive disorder assessment platform
Raguraj (2025) [227]	-AI analyzes behavioral, linguistic, physiological data for cognitive decline<br>-Deep learning detects subtle MCI patterns before clinical manifestation<br>-Supports personalized monitoring and	To analyze behavioral/linguistic/physiological data for cognitive decline	Multi-data analysis study	Deep learning detects MCI patterns; Before clinical manifestation; Supports personalized monitoring	MCI pattern detection, pre-clinical identification, monitoring personalization	Deep learning algorithms	Behavioral analysis, linguistic assessments, physiological monitoring

	population-wide screening						
Rai et al. (2020) [228]	-Altoida ADPS app discriminates healthy controls from prodromal AD<br>-Differentiates MCI converters from non-converters<br>-Validates novel digital biomarker for cognitive decline	To validate Altoida ADPS app for prodromal AD discrimination	App validation study	Discriminates controls from AD; Differentiates converters; Validates novel biomarker	AD discrimination accuracy, MCI conversion prediction, biomarker validity	Altoida ADPS app algorithms	Altoida ADPS app, digital biomarker assessment
Ren et al. (2025) [229]	-Digital twins enhance AD drug discovery with real-time tracking<br>-Applications include progression prediction, biomarker discovery, new targets<br>-Promising approach for revolutionizing AD drug discovery	To enhance AD drug discovery using digital twins	Drug discovery enhancement	Real-time tracking capability; Applications in progression/biomarkers; Revolutionizes drug discovery	Drug discovery efficiency, biomarker discovery rate, target identification	Digital twin drug discovery algorithms	AD progression tracking, biomarker discovery tools
Rudroff et al. (2024) [230]	-AI/ML improve early AD diagnosis accuracy and reliability<br>-Multi-modality studies enhance prediction performance<br>-Longitudinal studies crucial but face standardization challenges	To improve early AD diagnosis using AI/ML	Diagnostic improvement study	Improves accuracy and reliability; Multi-modality enhances performance; Longitudinal studies crucial	Diagnostic accuracy improvement, multi-modality performance, standardization needs	AI/ML diagnostic algorithms	Multi-modality AD assessments, longitudinal cognitive testing
Rutkowski et al. (2021) [231]	-Achieved ~90% accuracy using RF and neural networks for dementia onset<br>-MFDFA patterns from EEG differ between normal and MCI stages	To achieve ~90% accuracy using RF/neural networks for dementia	ML accuracy study	~90% accuracy for dementia onset; MFDFA patterns differ MCI/normal; Simple yet effective method	Dementia onset prediction (90%), EEG pattern discrimination	Random Forest (RF), neural networks	EEG assessments, MFDFA pattern analysis

	-Simple yet effective method for early dementia detection						
Rutkowski et al. (2023) [232]	-ML analyzes EEG network topology for early dementia prognosis<br>-Significant differences in network features between healthy and MCI<br>-High accuracy suggests applications in progression prediction	To analyze EEG network topology for early dementia using ML	Network analysis study	Significant differences healthy/MCI; Network features discriminate; High accuracy for progression	Network topology differences, progression prediction accuracy	ML for EEG network analysis	EEG network topology analysis, dementia progression markers
Sabbagh et al. (2019) [233]	-Digital technologies offer new prospects for MCI diagnosis/management<br>-Developed consensus for large-scale MCI screening algorithms<br>-Early detection crucial for disease-modifying therapies	To develop consensus for large-scale MCI screening using digital tech	Consensus development study	New prospects for MCI management; Consensus algorithm developed; Early detection crucial	Screening algorithm effectiveness, early detection capability	Large-scale MCI screening algorithms	Large-scale MCI screening tools, consensus-based protocols
Shah et al. (2023) [234]	-Evaluates ML (SVMs, CNNs) in neuropsychological research<br>-SVMs robust for neuroimaging, CNNs excel in visual input<br>-ML revolutionizes diagnosis enabling early, precise interventions	To evaluate ML (SVMs, CNNs) in neuropsychological research	ML technique comparison	SVMs robust for neuroimaging; CNNs excel in visual input; Revolutionizes diagnosis	Algorithm performance comparison, diagnostic revolution metrics	Support Vector Machines (SVMs), Convolutional Neural Networks (CNNs)	Neuroimaging-based assessments, visual cognitive tasks
Sizemore et al. (2024) [235]	-Digital twin of infant microbiome forecasts ecosystem trajectories<br>-Identifies infants at risk of poor head	To forecast infant microbiome ecosystem trajectories	Microbiome modeling study	Forecasts ecosystem trajectories; Identifies at-risk infants; Early transplantation mitigates risk	Growth trajectory prediction, risk identification accuracy, intervention effectiveness	Microbiome trajectory algorithms	Head circumference growth, microbiome assessment

	circumference growth<br>-Early transplantation may mitigate risk for significant cohort portion						
Song et al. (2025) [236]	-Digital biomarkers offer noninvasive alternative to traditional biomarkers<br>-Derived from EEG, eye movement, gait, speech analysis<br>-Smartphones and wearables emerging as diagnostic tools	To evaluate digital biomarkers as noninvasive alternatives	Biomarker alternative study	Noninvasive alternative effective; Derived from EEG, eye, gait, speech; Smartphones as diagnostic tools	Biomarker effectiveness, diagnostic tool validation, accessibility metrics	Digital biomarker extraction algorithms	EEG, eye movement tracking, gait analysis, speech analysis
Souillard-Mandar et al. (2021) [237]	-DCTclock improves early cognitive impairment detection<br>-Higher AUC and sensitivity/specificit y than MMSE<br>-Differentiates various cognitive impairment stages including MCI subtypes	To improve early cognitive impairment detection using DCTclock	DCTclock validation study	Higher AUC than MMSE; Superior sensitivity/specificity; Differentiates impairment stages	Detection accuracy (AUC), sensitivity/specifi city, stage differentiation	DCTclock AI algorithms	DCTclock digital assessment, MMSE comparison
Sprint et al. (2024) [238]	-HDTwin achieves 0.81 peak accuracy for cognitive diagnoses<br>-Multiple information sources fusion superior to single sources<br>-Enhances accuracy, explainability, and early detection strategies	To achieve cognitive diagnosis accuracy using HDTwin	HDTwin accuracy study	0.81 peak accuracy achieved; Multiple source fusion superior; Enhances early detection	Diagnostic accuracy (0.81), fusion effectiveness, early detection enhancement	HDTwin (Large Language Models for cognitive diagnosis)	HDTwin cognitive assessment platform, multi-source health data
Tacchino et al. (2023) [239]	-Digital twins enhance MS cognitive phenotyping	To enhance MS cognitive phenotyping using digital twins	MS phenotyping enhancement	Improves accessibility/effective ness; Tele-rehabilitation	Cognitive phenotyping accuracy, rehabilitation	Digital twin cognitive phenotyping algorithms	MS cognitive assessment battery, tele-cognitive rehabilitation

	-Tele-cognitive-rehabilitation improves accessibility and effectiveness<br>-Integration improves outcomes and transfers to everyday activities			effective; Transfers to daily activities	effectiveness, daily transfer		
Tang et al. (2020) [240]	-Adaptive AI dialogue agent adjusts questioning for MCI screening<br>-Cost-effective and scalable for large-scale preclinical screening<br>-Achieves comparable or better performance than traditional assessments	To develop adaptive AI dialogue agent for MCI screening	AI agent development	Adjusts questioning for screening; Cost-effective and scalable; Comparable to traditional assessments	Screening accuracy, scalability metrics, cost-effectiveness	Adaptive AI dialogue algorithms	AI dialogue-based MCI screening, adaptive questionnaires
Tarnanas et al. (2018) [241]	-Predicts AD development 18-24 months before symptoms<br>-NMI tracks micro-errors with >94% diagnostic accuracy for MCI<br>-Detects subtle motor planning alterations preceding clinical symptoms	To predict AD development 18-24 months before symptoms	Early prediction study	Predicts 18-24 months early; >94% diagnostic accuracy for MCI; Detects subtle motor alterations	Early prediction window (18-24 months), diagnostic accuracy (>94%)	Error tracking algorithms, NMI (Neurocogniti ve Motion Index)	Motor planning assessments, micro-error tracking, complex task analysis
Tarnanas et al. (2021) [242]	-Common digital neuro signature identified for AD and PD<br>-Contains 20+ shared features capturing motor/non-motor symptoms<br>-Model identifies therapy-eligible patients using SHAP values	To identify common digital neuro signature for AD and PD	Signature identification study	Common signature identified; 20+ shared features; Identifies therapy-eligible patients	Signature accuracy, feature identification, patient selection accuracy	SHAP values, digital signature algorithms	Motor/non-motor symptom assessments, digital neuro signatures

Termine et al. (2021) [243]	-Deep learning enhances understanding of neurodegenerative diseases<br>-Identifies biomarkers using handwriting, speech, movement dynamics<br>-Crucial for precision medicine and personalized interventions	To enhance neurodegenerative understanding using deep learning	Deep learning enhancement study	Identifies biomarkers effectively; Uses handwriting, speech, movement; Crucial for precision medicine	Biomarker identification accuracy, precision medicine applicability	Deep neural networks	Handwriting analysis, speech assessment, movement dynamics
Thangaraj et al. (2024) [244]	-Digital twins advance cardiovascular clinical decision-making<br>-Evolving with new data modalities and AI advances<br>-Highlights future applications and ethical considerations	To advance cardiovascular decision-making using digital twins	Clinical decision advancement	Advances clinical decisions; Evolving with new modalities; Highlights future applications	Decision-making improvement, modality integration, ethical consideration	Digital twin clinical decision algorithms	Cardiovascular-cognitive interaction assessments
Tortora et al. (2025) [245]	-Digital twins increasing in health sector applications<br>-Big data and AI accelerate research and development<br>-Healthcare applications still in infancy, neurology as cutting-edge area	To assess digital twin growth in health sector applications	Health sector assessment	Increasing health applications; Big data/AI accelerate research; Neurology as cutting-edge area	Application growth rate, research acceleration, field advancement	Big data and AI algorithms	Neurological assessment integration with digital health
Tosun (2025) [246]	-Develops multi-disciplinary/modality biomarkers for early detection<br>-Detects subtle changes before clinical symptoms<br>-Aims to improve outcomes through early intervention	To develop multi-disciplinary biomarkers for early detection	Biomarker development study	Detects subtle pre-clinical changes; Before symptoms appear; Improves early intervention	Pre-clinical detection sensitivity, intervention timing improvement	Multi-disciplinary biomarker algorithms	Multi-disciplinary assessment battery, pre-clinical detection tools

Voigt et al. (2021) [247]	-Digital twins key advancement for individualized MS management<br>-Combined with tele-rehabilitation improves cognitive outcomes<br>-Delivers more effective and accessible tailored interventions	To advance individualized MS management using digital twins	MS management advancement	Key advancement for MS; Combined with tele-rehabilitation; Delivers tailored interventions	MS management improvement, cognitive outcome enhancement, intervention effectiveness	Digital twin MS management algorithms	MS cognitive rehabilitation, individualized assessment protocols
Wang et al. (2024) [248]	-Virtual brain twins are personalized, generative, adaptive models<br>-Personalization involves brain assembly, connectivity mapping, ML parameters<br>-Applied to healthy aging and five clinical diseases	To create personalized, generative, adaptive virtual brain twins	Virtual brain twin creation	Personalized brain assembly; Connectivity mapping with ML; Applied to aging and diseases	Personalization accuracy, generative capability, adaptation metrics	ML for brain connectivity mapping	Brain connectivity assessments, personalized cognitive mapping
Wang et al. (2024) [249]	-TWIN-GPT enhances clinical trial outcome prediction accuracy<br>-Demonstrates exceptional fidelity, utility, and privacy performance<br>-Provides practical evidence for digital twin healthcare applications	To enhance clinical trial prediction using TWIN-GPT	Trial prediction enhancement	Exceptional fidelity and utility; Privacy performance demonstrated; Practical healthcare evidence	Prediction accuracy enhancement, privacy compliance, utility metrics	TWIN-GPT (Large Language Model)	Clinical trial cognitive endpoints, synthetic patient data
Wickramasinghe et al. (2022) [250]	-Digital twins improve dementia care precision and personalization<br>-Developed as clinical decision support tool<br>-Aligns with value-based healthcare	To improve dementia care precision using digital twins	Dementia care improvement	Improves precision/personalization; Clinical decision support tool; Aligns with value-based care	Care precision improvement, personalization metrics, value alignment	Digital twin dementia care algorithms	Dementia care assessment tools, clinical decision support

	addressing growing needs						
Xiong et al. (2023) [251]	-DTB offers insights into intelligence and neurological disorders<br>-Provides new paradigm for brain function understanding<br>-Applications in neuromodulation and drug development	To provide insights into intelligence and neurological disorders using DTB	DTB insight study	Offers intelligence insights; New paradigm for brain function; Applications in neuromodulation	Intelligence understanding, neurological disorder insights, neuromodulation applications	Digital Twin Brain (DTB) algorithms	Intelligence assessment, brain function evaluation tools
Yousefi et al. (2024) [252]	-ML algorithms show promise surpassing traditional diagnostics<br>-Identified 12 algorithms for dementia, 18 for PD, 14 for MCI<br>-Integration with conventional practice crucial for future advances	To demonstrate ML algorithms surpassing traditional diagnostics	ML superiority demonstration	ML surpasses traditional methods; 12 algorithms for dementia, 18 PD, 14 MCI; Integration crucial	Algorithm performance superiority, diagnostic improvement metrics, integration effectiveness	Multiple ML algorithms (12 for dementia, 18 for PD, 14 for MCI)	Traditional vs ML-based cognitive assessments
