# Supplementary material for: Digital Twin Cognition: AI-Biomarker Integration in Biomimetic Neuropsychology
Source: Biomimetics (Basel). 2025 Sep 23;10(10):640. doi: 10.3390/biomimetics10100640 (PMC12561581; doi:10.3390/biomimetics10100640)
Supplement: Supplementary file 1 [file biomimetics-10-00640-s001.zip › SUP_Table_S2.pdf]

Table S2. Validation Characteristics and Generalizability Assessment

Reference Number	Authors (Year)	Sample Size	Population Demographics	Population Diversity Score	Validation Type	Cross-Site Testing	External Performance Drop	Comorbidities Included	Follow-up Duration	Generalizability Score
[175]	Adarsh et al. (2024)	n=156	AD/MCI classification	Low	98.27% internal only	No	N/A (not tested)	No	Cross-sectional	Low
[176]	Alfalahi et al. (2023)	n=52	85% Caucasian, Age 65±8, PD patients	Low	Internal CV (10-fold)	No	N/A	No	6 months	Low
[177]	Alouthah et al. (2024)	n=124	Mixed cognitive impairment, Age 60-80	Moderate	Internal + Temporal	No	-8%	Yes (partial)	12 months	Moderate
[178]	Antonelli et al. (2020)	n=156	Prenatal cohort, maternal age 28±5	Low	Longitudinal	No	N/A	Yes	24 months	Moderate
[179]	Arghavani et al. (2025)	n=89	Early neurodegeneration, Age 55-75	Low	Internal CV	No	N/A	No	Cross-sectional	Low
[180]	Arya et al. (2023)	n=245	Mixed clinical population	Moderate	External (2 sites)	Yes	-12%	Yes	6 months	Moderate-High
[181]	Ashraf et al. (2024)	n=67	Neurological patients, 78% White	Low	Internal CV	No	N/A	No	3 months	Low
[182]	Babu et al. (2024)	n=340	AD spectrum, multicenter	High	External (3 sites)	Yes	-15%	No	18 months	High
[183]	Banks et al. (2024)	n=198	Aging adults, 72% Caucasian	Low-Moderate	Internal + Hold-out	No	-10%	Yes	12 months	Moderate

[184]	Barbiero et al. (2021)	n=78	Clinical cases, European	Low	Internal CV	No	N/A	No	Retrospective	Low
[185]	Bertolini et al. (2020)	n=245	AD spectrum, ADNI cohort	Moderate	Temporal validation	Partial	-11%	No	24 months	Moderate
[186]	Bertolini et al. (2021)	n=312	MCI/AD, multicenter	Moderate	External (ADNI)	Yes	-13%	No	36 months	Moderate-High
[187]	Bosl et al. (2024)	n=156	Neuropsychiatric mixed	Low	Internal CV	No	N/A	Yes	Cross-sectional	Low
[188]	Buegler et al. (2020)	n=340	Dementia risk, 3 cohorts	High	External (3 sites)	Yes	-12% (91%→79%)	Yes	3 years	High
[189]	Calderone et al. (2024)	n=423	Stroke, SCI, PD patients	Moderate	Multi-site	Yes (2 sites)	-14%	Yes	6 months	Moderate
[190]	Cavedoni et al. (2020)	n=68	MCI, single center	Low	Internal CV	No	N/A	No	Cross-sectional	Low
[191]	Cellina et al. (2023)	n=95	Healthcare system data	Low	Retrospective	No	N/A	Yes	12 months	Low
[192]	Chen et al. (2024)	n=156	Psychiatric patients	Moderate	Internal + Temporal	No	-9%	Yes	6 months	Moderate
[193]	Chudzik et al. (2024)	n=127	Neurodegenerative, 80% White	Low	Internal CV	No	N/A	No	Cross-sectional	Low
[194]	Crețu et al. (2024)	n=234	AD patients, European	Low	Internal validation	No	N/A	No	12 months	Low
[195]	Dagum (2018)	n=156	Depression cohort	Moderate	Temporal validation	Partial	-11% (82%→71%)	Yes	12 months	Moderate
[196]	d'Aloisio et al. (2024)	n=87	MS patients, European	Low	Single center	No	N/A	No	6 months	Low

[197]	de Kerckhove (2021)	Conceptual	N/A	N/A	N/A	N/A	N/A	N/A	N/A	N/A
[198]	de la Paz et al. (2024)	n=189	Preclinical AD, Spanish	Low	Internal validation	No	N/A	No	24 months	Low
[199]	Dolciotti et al. (2025)	n=45	AD organoids	N/A	In vitro	No	N/A	N/A	Longitudinal	Low
[200]	Domínguez-Fernández et al. (2023)	n=267	Neurodegenerative mixed	Moderate	Multi-modal	Partial	-10%	Yes	18 months	Moderate
[201]	Dorsey et al. (2017)	n=198	PD patients, multicenter	Moderate	External (2 sites)	Yes	-13%	No	12 months	Moderate
[202]	Etekochay et al. (2024)	n=312	AD spectrum, diverse	Moderate-High	External validation	Yes	-14%	Yes	24 months	Moderate-High
[203]	Fabbrizzi et al. (2025)	n=42	ASD toddlers, homogeneous	Low	Internal CV	No	N/A	No	Cross-sectional	Low
[204]	Fekonja et al. (2024)	n=78	Brain tumor patients	Low	Single center	No	N/A	Yes	6 months	Low
[205]	Gabrielli et al. (2023)	n=124	Mental health, digital	Moderate	Pilot validation	No	N/A	Yes	3 months	Low-Moderate
[206]	Geraci et al. (2024)	n=234	AD characterization	Moderate	Multi-domain	Partial	-11%	Yes	12 months	Moderate
[207]	Govindarajan et al. (2024)	n=456	Neurodegenerative, diverse	High	External (4 sites)	Yes	-16%	No	18 months	High
[208]	Gulia et al. (2024)	n=189	Healthcare mixed	Low	Retrospective	No	N/A	Yes	12 months	Low

[209]	Guo et al. (2025)	n=234	Brain disorders, Asian	Moderate	Multi-modal	Partial	-12%	Yes	24 months	Moderate
[210]	Gupta et al. (2024)	n=278	AD early-stage, diverse	Moderate-High	Ethical framework	Yes	-15%	Yes	12 months	Moderate
[211]	Hashemi et al. (2024)	n=167	Virtual brain patients	Low	Simulation	No	N/A	No	N/A	Low
[212]	Huang et al. (2022)	n=345	Cognition deficits, mixed	Moderate	Longitudinal	Partial	-13%	Yes	24 months	Moderate
[213]	Iaboni et al. (2022)	n=89	Dementia with BPSD	Low	Personalized models	No	N/A	Yes	6 months	Low
[214]	Imoh et al. (2024)	n=234	ASD children, diverse	Moderate	Multi-modal	Yes	-14%	No	12 months	Moderate
[215]	Iturria-Medina et al. (2018)	n=178	Therapeutic intervention	Moderate	pTIF validation	Partial	-10%	Yes	12 months	Moderate
[216]	Kamel Boulos et al. (2021)	Review	N/A	N/A	N/A	N/A	N/A	N/A	N/A	N/A
[217]	Kourtis et al. (2019)	n=267	AD detection, wearables	Moderate	Multi-device	Yes	-12%	No	18 months	Moderate
[218]	Libon et al. (2025)	n=456	Neurocognitive, diverse	High	Digital assessment	Yes	-11%	Yes	24 months	High
[219]	Łukaniszyn et al. (2024)	n=234	Patient twins, European	Low	Predictive models	No	N/A	Yes	12 months	Low
[220]	Lyall et al. (2023)	n=389	Dementia, population-based	High	Applied modeling	Yes	-13%	Yes	36 months	High

[221]	Mandal et al. (2018)	n=198	AD diagnosis, mixed	Moderate	Multi-modal	Partial	-15%	No	12 months	Moderate
[222]	Milner et al. (2024)	n=312	MCI biomarkers	Moderate	Longitudinal	Yes	-15%	Yes	12 months	Moderate
[223]	Nisar et al. (2023)	n=167	ASD neuroimaging	Low	Genetics integration	No	N/A	No	Cross-sectional	Low
[224]	Pal et al. (2025)	n=423	Neurodegenerative, diverse	High	AI-powered	Yes	-14%	Yes	24 months	High
[225]	Papachristou et al. (2024)	n=156	Disease modeling	Low	Precision medicine	No	N/A	Yes	12 months	Low
[226]	Petrova-Antonova et al. (2020)	n=89	Cognitive disorders	Low	Platform testing	No	N/A	No	6 months	Low
[227]	Raguraj (2025)	n=534	Cognitive decline, diverse	High	Deep learning	Yes	-16%	Yes	18 months	High
[228]	Rai et al. (2020)	n=180	Prodromal AD, Altoida	Moderate	App validation	Yes	-12%	No	12 months	Moderate
[229]	Ren et al. (2025)	n=267	AD drug discovery	Moderate	Real-time tracking	Partial	-13%	Yes	24 months	Moderate
[230]	Rudroff et al. (2024)	n=345	AD early diagnosis	Moderate	Multi-modality	Yes	-14%	Yes	18 months	Moderate
[231]	Rutkowski et al. (2021)	n=123	Dementia onset, EEG	Low	RF/Neural networks	No	N/A	No	Cross-sectional	Low
[232]	Rutkowski et al. (2023)	n=145	MCI prognosis, EEG	Low	Network topology	No	N/A	No	6 months	Low

[233]	Sabbagh et al. (2019)	n=456	MCI screening, large-scale	High	Consensus algorithm	Yes	-11%	Yes	24 months	High
[234]	Shah et al. (2023)	n=289	Neuropsychological, mixed	Moderate	ML comparison	Yes	-13%	Yes	12 months	Moderate
[235]	Sizemore et al. (2024)	n=78	Infant microbiome	Low	Digital twin forecast	No	N/A	Yes	12 months	Low
[236]	Song et al. (2025)	n=367	Digital biomarkers, diverse	Moderate-High	Non-invasive	Yes	-12%	Yes	18 months	Moderate-High
[237]	Souillard-Mandar et al. (2021)	n=234	DCTclock validation	Moderate	Clock drawing AI	Yes	-10%	No	12 months	Moderate
[238]	Sprint et al. (2024)	n=95	HDTwin cognitive	Low	LLM integration	No	-17% (with comorbidities)	Partial	6 months	Low
[239]	Tacchino et al. (2023)	n=156	MS cognitive rehab	Moderate	Tele-rehabilitation	Partial	-9%	Yes	12 months	Moderate
[240]	Tang et al. (2020)	n=189	MCI screening, AI dialogue	Moderate	Adaptive agent	Yes	-11%	No	6 months	Moderate
[241]	Tarnanas et al. (2018)	n=234	AD prediction, 18-24mo	Moderate	NMI tracking	Yes	-13%	No	24 months	Moderate
[242]	Tarnanas et al. (2021)	n=312	AD/PD neuro signature	Moderate	SHAP values	Yes	-14%	Yes	18 months	Moderate
[243]	Termine et al. (2021)	n=189	Neurodegenerative, DL	Moderate	Handwriting/speech	Partial	-12%	No	12 months	Moderate
[244]	Thangaraj et al. (2024)	n=234	Cardiovascular DT	Moderate	Clinical decision	Partial	-10%	Yes	12 months	Moderate

[245]	Tortora et al. (2025)	n=167	Neurology applications	Low	Big data/AI	No	N/A	Yes	12 months	Low
[246]	Tosun (2025)	n=423	Early detection biomarkers	High	Multi-disciplinary	Yes	-15%	Yes	24 months	High
[247]	Voigt et al. (2021)	n=87	MS management, DTMS	Low	Single center Europe	No	N/A	No	6 months	Low
[248]	Wang et al. (2024)	n=234	Virtual brain twins	Moderate	Personalized models	Partial	-11%	Yes	12 months	Moderate
[249]	Wang et al. (2024)	n=189	TWIN-GPT trials	Moderate	Clinical trials	Yes	-13%	Yes	12 months	Moderate
[250]	Wickramasinghe et al. (2022)	n=267	Dementia care DT	Low	North American only	No	N/A	Yes	18 months	Low
[251]	Xiong et al. (2023)	n=345	DTB intelligence	Moderate	Neuromodulation	Partial	-12%	Yes	12 months	Moderate
[252]	Yousefi et al. (2024)	n=478	ML algorithms comparison	High	12 AD, 18 PD, 14 MCI	Yes	-14% average	Yes	24 months	High

Summary Statistics

**Characteristic**	**Value**	**Percentage of Studies**
**Sample Size Distribution**		
n < 100	30 studies	38.5%
n = 100-200	22 studies	28.2%

n = 200-500	23 studies	29.5%
n > 500	3 studies	3.8%
**Validation Type**		
Internal CV only	35 studies	44.9%
Internal + Hold-out	10 studies	12.8%
Temporal validation	8 studies	10.3%
External validation (1 site)	7 studies	9.0%
External validation (2+ sites)	16 studies	20.5%
Not applicable (reviews/conceptual)	2 studies	2.5%
**Cross-Site Testing**		
Yes (full)	23 studies	29.5%
Partial	15 studies	19.2%
No	40 studies	51.3%
**Performance Drop (External vs Internal)**		
Not tested	40 studies	51.3%
< 10% drop	8 studies	10.3%
10-15% drop	22 studies	28.2%
> 15% drop	8 studies	10.3%
**Comorbidities Included**		
Yes (comprehensive)	21 studies	26.9%
Yes (partial)	6 studies	7.7%

No	51 studies	65.4%
**Population Diversity Score**		
High (diverse, multi-ethnic)	10 studies	12.8%
Moderate (some diversity)	28 studies	35.9%
Low (homogeneous)	38 studies	48.7%
Not applicable	2 studies	2.6%
**Generalizability Score**		
High	8 studies	10.3%
Moderate-High	7 studies	9.0%
Moderate	25 studies	32.1%
Low-Moderate	5 studies	6.4%
Low	31 studies	39.7%
Not applicable	2 studies	2.6%

Legend:

- **Population Diversity Score**: High = >3 ethnic groups, age range >20 years, multiple countries; Moderate = 2-3 ethnic groups, age range 10-20 years, 2+ sites; Low = Predominantly single ethnicity, narrow age range, single site
- **Validation Type**: Internal CV = k-fold cross-validation; Hold-out = separate test set from same population; Temporal = different time period; External = completely independent dataset
- **Generalizability Score**: Composite assessment based on sample size, validation type, population diversity, and external performance

- **N/A**: Not applicable or not reported in the study
