# Supplementary material for: Digital Twin Cognition: AI-Biomarker Integration in Biomimetic Neuropsychology
Source: Biomimetics (Basel). 2025 Sep 23;10(10):640. doi: 10.3390/biomimetics10100640 (PMC12561581; doi:10.3390/biomimetics10100640)
Supplement: Supplementary file 1 [file biomimetics-10-00640-s001.zip › Sup_Table_S3_Abbreviation List.pdf]

Table S3. Abbreviations List

Clinical and Medical Terms

BPSD: Behavioral and Psychological Symptoms of Dementia
CDR-SB: Clinical Dementia Rating Sum-of-Boxes
DSM-5: Diagnostic and Statistical Manual of Mental Disorders, 5th Edition
EHR: Electronic Health Record
MMSE: Mini-Mental State Examination
QoL: Quality of Life

Diseases and Conditions

AD: Alzheimer's Disease
ASD: Autism Spectrum Disorder
MCI: Mild Cognitive Impairment
MS: Multiple Sclerosis
PD: Parkinson's Disease
SCI: Spinal Cord Injury

Artificial Intelligence and Computing

AI: Artificial Intelligence
API: Application Programming Interface
CNN: Convolutional Neural Network
CPU: Central Processing Unit
CRBM: Conditional Restricted Boltzmann Machine
DL: Deep Learning
DT: Digital Twin
DTB: Digital Twin Brain
DTMS: Digital Twin for Multiple Sclerosis
GAN: Generative Adversarial Network
GNN: Graph Neural Network
GPU: Graphics Processing Unit
HDT: Human Digital Twin
LLM: Large Language Model
LSTM: Long Short-Term Memory
ML: Machine Learning
NLP: Natural Language Processing
RL: Reinforcement Learning
RNN: Recurrent Neural Network
TWIN-GPT: Twin Generative Pre-trained Transformer
VBT: Virtual Brain Twin
XAI: Explainable Artificial Intelligence

Neuroimaging and Biomarkers

DTI: Diffusion Tensor Imaging
EEG: Electroencephalography
fMRI: Functional Magnetic Resonance Imaging

HT: Hold Time
MRI: Magnetic Resonance Imaging
nFT: Normalized Flight Time
nP: Normalized Pressure
PET: Positron Emission Tomography

Statistical and Mathematical Methods

AUC: Area Under Curve
CI: Confidence Interval
FRST: Fuzzy Rough Set Theory
ICC: Intraclass Correlation Coefficient
LR: Logistic Regression
MFDFA: Multifractal Detrended Fluctuation Analysis
MKSCDDL: Multi-feature Kernel Supervised within-class-similar Discrimination
NMI: Normalized Mutual Information
RF: Random Forest
ROC: Receiver Operating Characteristic
RST: Rough Set Theory
SHAP: SHapley Additive exPlanations
SMOTE: Synthetic Minority Over-sampling Technique
SVM: Support Vector Machine

Treatment and Intervention Technologies

ADPS: Altoida Digital Predictive System
AR: Augmented Reality
DCT: Digital Clock Test
tDCS: Transcranial Direct Current Stimulation
TMS: Transcranial Magnetic Stimulation
VR: Virtual Reality

Materials and Methods

COF: Covalent Organic Framework
FEDE: Finite Element-based Digital Encephalon
iPSC: Induced Pluripotent Stem Cell
MOF: Metal-Organic Framework
MXene: 2D Transition Metal Carbides/Carbonitrides/Nitrides
pTIF: Personalized Therapeutic Intervention Fingerprint

Standards and Organizations

EMA: European Medicines Agency
FDA: Food and Drug Administration
FHIR: Fast Healthcare Interoperability Resources
GDPR: General Data Protection Regulation
MeSH: Medical Subject Headings
PRISMA: Preferred Reporting Items for Systematic Reviews and Meta-Analyses
QUADAS-2: Quality Assessment of Diagnostic Accuracy Studies, Version 2
