# Supplementary material for: Digital Twin Cognition: AI-Biomarker Integration in Biomimetic Neuropsychology
Source: Biomimetics (Basel). 2025 Sep 23;10(10):640. doi: 10.3390/biomimetics10100640 (PMC12561581; doi:10.3390/biomimetics10100640)
Supplement: Supplementary file 1 [file biomimetics-10-00640-s001.zip › SUP_Table_S4.pdf]

**Table S4.** Comprehensive Framework Performance with Validation Quality Assessment

| Framework/Biomarker Integration | Biomarker Types | Algorithms Used | Performance Metrics | Clinical Impact | Validation Quality | Sample Size | External Validation | Population Diversity | Implementation Status |
|---|---|---|---|---|---|---|---|---|---|
| Digital Twin for MS (DTMS) [247] | Neuroimaging + Physiological + Behavioral | Unified predictive model, Federated learning | 22 relevance matches, 89% symptom detection | Precision MS management (+35% treatment outcomes)* | Moderate | n=87 (single center) | No | Low (European only) | Clinical deployment |
| Explainable Digital Phenotyping [176] | Keystroke dynamics, Motor symptoms, Social markers | Ensemble regression (neuroQWERTY), Bayesian networks | 89% early PD detection, 90% dementia prediction (internal) | 25% faster diagnosis | Low | n=52 | No | Low (85% Caucasian) | Research validation |
| Multimodal Early Detection [182, 188, 207] | EEG + fMRI + DTI + Digital phenotyping | XGBoost, CNN, GNN, Deep learning ensembles | Internal: 85-95% accuracy; External: 78-87% accuracy | 40% improvement in early detection sensitivity | Low-Moderate | n=125-340 | 2/5 studies | Moderate | Multi-site trials |
| Personalized Cognitive Models [190, 192] | Speech, VR-based assessments, Smartphone data | SVM, Naïve Bayes, Decision trees | r = 0.82 stability over 6 months; 75-85% accuracy | 15-20% reduction in disease progression | Moderate | n=156-220 | 1/3 studies | Low (single site) | Pilot studies |
| Brain Structure Digital Twins (FEDE) [203] | Anatomical MRI + Connectivity maps | FEDE pipeline, Graph Neural Networks, GANs | 95% structural fidelity (internal), 88-92% prediction accuracy | Real-time monitoring capability | Low | n=42 (ASD toddlers) | No | Low (homogeneous) | Proof of concept |

| | | | | | | | | | |
|---|---|---|---|---|---|---|---|---|---|
| Continuous Monitoring Systems [195, 228] | Circadian rhythms, Sleep patterns, Daily activities | CRBMs, Signal processing, Tensor factorization | 82-90% longitudinal accuracy; 71% prospective | Timely intervention adjustments | Moderate-High | n=180-320 | Yes (2 sites) | Moderate | Development phase |
| HDTwin Cognitive Diagnosis [238] | Clinical notes + Neuroimaging + Behavioral | Large Language Models, Multi-source fusion | 0.81 peak accuracy (internal); 0.64 with comorbidities | Enhanced explainability | Low | n=95 | No | Low | Research phase |
| Digital Biomarker Prognostic [188] | Clinical + Wearable + Cognitive tests | XGBoost with SMOTE | 91% internal; 79% external validation | 3-year dementia risk stratification | High | n=340 | Yes (3 sites) | Moderate | Clinical trials |
| Behavioral Digital Twin [195] | Smartphone usage + Call patterns + App data | Logistic regression, Signal processing | 82% retrospective; 71% prospective | Continuous ecological assessment | Moderate | n=156 | Partial (temporal) | Low | Pilot deployment |
| Graph-based Patient Models [184] | Multi-organ data + Clinical history | GNN, GAN, Graph Network Blocks | 88% internal validation | Panoramic patient view | Low | n=78 | No | Low | Concept validation |
| CRBM Disease Progression [185, 186] | Clinical scales + Biomarkers | Conditional Restricted Boltzmann Machines | 89% (6-month); 76% (24-month) | MCI to AD forecasting | Moderate | n=245 | Partial | Moderate | Research validation |
| VR-Integrated Assessment [190] | Gait kinematics + VR metrics + ML | Supervised learning ensemble | 85% MCI detection | Ecological validity | Low | n=68 | No | Low | Pilot phase |

**Table S4a.** Validation Quality Assessment Summary

| Quality Level | Number of Studies (%) | Criteria | Typical Performance Drop |
|---|---|---|---|
| **High Quality** | 8 studies (10.3%) | • External validation in 3+ sites<br>• n>300 with diverse demographics<br>• Prospective design, 12+ months<br>• Includes comorbidities | <10% degradation |
| **Moderate Quality** | 25 studies (32%) | • External/temporal validation 1-2 sites<br>• n=150-300, some diversity<br>• Mixed retrospective/prospective<br>• Some clinical complexity | 10-15% degradation |
| **Low Quality** | 45 studies (57.7%) | • Internal validation only (k-fold CV)<br>• n<150, homogeneous population<br>• Retrospective only<br>• Excludes comorbidities | 15-25% degradation |

**Table S4b.** Key Performance Insights

| Validation Factor | Impact on Performance | Studies Affected |
|---|---|---|
| Internal vs External Validation | 10-18% accuracy inflation with internal only | 70.5% of studies |
| Small Sample Size (n<100) | 15-25% performance drop when validated externally | 38% of studies |
| Population Homogeneity | Limited to specific demographics (>70% Caucasian) | 78% of studies |
| Temporal Stability | 8-12% annual decline without retraining | 14 studies tracked |
| Comorbidity Inclusion | 15-22% accuracy reduction with real-world complexity | 73% exclude comorbidities |
